# The ecology of immune state in a wild mammal, *Mus musculus domesticus*

**DOI:** 10.1371/journal.pbio.2003538

**Published:** 2018-04-13

**Authors:** Stephen Abolins, Luke Lazarou, Laura Weldon, Louise Hughes, Elizabeth C. King, Paul Drescher, Michael J. O. Pocock, Julius C. R. Hafalla, Eleanor M. Riley, Mark Viney

**Affiliations:** 1 School of Biological Sciences, University of Bristol, Bristol, United Kingdom; 2 Department of Immunology and Infection, London School of Hygiene and Tropical Medicine, London, United Kingdom; 3 Centre for Ecology & Hydrology, Wallingford, Oxfordshire, United Kingdom; 4 The Roslin Institute and Royal (Dick) School of Veterinary Studies, University of Edinburgh, Easter Bush, Midlothian, United Kingdom; Stanford University, United States of America

## Abstract

The immune state of wild animals is largely unknown. Knowing this and what affects it is important in understanding how infection and disease affects wild animals. The immune state of wild animals is also important in understanding the biology of their pathogens, which is directly relevant to explaining pathogen spillover among species, including to humans. The paucity of knowledge about wild animals' immune state is in stark contrast to our exquisitely detailed understanding of the immunobiology of laboratory animals. Making an immune response is costly, and many factors (such as age, sex, infection status, and body condition) have individually been shown to constrain or promote immune responses. But, whether or not these factors affect immune responses and immune state in wild animals, their relative importance, and how they interact (or do not) are unknown. Here, we have investigated the immune ecology of wild house mice—the same species as the laboratory mouse—as an example of a wild mammal, characterising their adaptive humoral, adaptive cellular, and innate immune state. Firstly, we show how immune variation is structured among mouse populations, finding that there can be extensive immune discordance among neighbouring populations. Secondly, we identify the principal factors that underlie the immunological differences among mice, showing that body condition promotes and age constrains individuals’ immune state, while factors such as microparasite infection and season are comparatively unimportant. By applying a multifactorial analysis to an immune system-wide analysis, our results bring a new and unified understanding of the immunobiology of a wild mammal.

## Author summary

The immune state of wild animals—and the factors that affect this—is largely unknown. Knowing this is important in understanding how infection and disease affects wild animals, and the biology of their pathogens. The paucity of knowledge about wild animals' immune state is in stark contrast to our exquisitely detailed understanding of the immunobiology of laboratory animals. Here, we have investigated the immune ecology of wild house mice—the same species as the laboratory mouse—characterising their adaptive humoral, adaptive cellular, and innate immune state. Firstly, we investigated how immune variation is structured among mouse populations, finding that there can be extensive immune discordance among neighbouring populations. Secondly, we identified the principal factors that underlie the immunological differences among mice, showing that body condition promotes and age constrains individuals’ immune state. By applying a multifactorial analysis to an immune system-wide analysis, our results bring a new and unified understanding of the immunobiology of a wild mammal.

## Introduction

Immune systems protect animals from infection and disease. The mammalian immune system reacts to antigenic exposure with a multitude of cellular and humoral responses, including cytotoxicity, phagocytosis, and the production of cytokines and antibodies. The ultimate result of these responses—which we call immune function—is to reduce the effects of infection on an individual. While it is possible to measure many aspects of the immune response (such as the number and state of cells and the concentrations of antibodies and cytokines, both as observed in an animal and after experimental perturbation), the relationship between these measures, their effect on infections, and the consequences for an animal’s health, and ultimately its fitness, is much less clear. Although the terminology is debatable, for clarity we will refer to the observed state of an animal’s immune system as its immune state and so differentiate this from immune response, which we reserve to refer to specific responses to a known antigenic challenge. A wild animal’s immune state is therefore a consequence of its immune responses to the myriad, largely unknown, antigenic challenges that it receives during its daily life.

The immune state and immune responses of laboratory animals, particularly mice (*Mus musculus domesticus*), are extremely well known, which contrasts starkly with what is known about the immune state of wild animals [1]. Because the behaviour of the immune system is highly context dependent, the a priori expectation is that the immune state of wild animals will differ from that of laboratory animals. This assumption is supported by the relatively few studies that have compared wild and laboratory mice [1–7]. For example, 2 studies have shown that wild mice respond more strongly to immunization than laboratory mice [1,4]; comparison of wild and laboratory mice has shown that the wild mice have higher proportions of activated CD4^+^ T cells [2]. A detailed analysis of the adaptive humoral, adaptive cellular, and innate immune state of wild mice found that, compared with laboratory mice, the immune systems of wild mice were in a highly activated state, seen as elevated serum antibody and acute phase protein concentrations; proportionately greater CD4^+^ and CD8^+^ effector T cell populations; highly activated natural killer (NK) cells; and a myeloid cell population hitherto unknown from laboratory mice [1]. This highly activated state is likely due to the many infections that wild mice have experienced (and the consequent intense antigenic challenge to which they are subject) compared to laboratory mice [1]. In contrast to this comparatively activated state, wild mouse cytokine responses to pathogen-associated molecular patterns (PAMPs) were similar to, or lower than, those of laboratory mice, possibly representing the impact of homeostatic mechanisms among wild mice to avoid immunopathology in the face of sustained antigenic challenge. These results also highlighted the very extensive interindividual variation among wild mice in their immune state [1]. The importance of the environment in driving differences between wild and laboratory animals' immune systems has also been shown by experiments that cohoused wild and laboratory mice (thus exposing the laboratory mice to a range of infections), resulting in the laboratory mice acquiring more effector memory cells as well as more tissue-resident memory T cells, compared with control laboratory mice [2]. These trends also appear to be consistent in other wild rodents, though there are probably interspecific differences too [7].

These immunological differences between wild and laboratory mice therefore imply that laboratory-based models cannot fully inform us about the immune state or function of wild animal populations and, rather, that direct study of wild populations is required. Understanding the immune function of wild populations is necessary to understand their biology, but also necessary to understand the biology of the populations of pathogens with which they are naturally infected. Animals' immune responses are the environment in which pathogens live and evolve, and this immunological environment exerts a strong selective force on these pathogens. The zoonotic spillover of infections, particularly from wild animals to humans, is currently of enormous international importance [8,9]. An important, understudied aspect of this is the immunological state of wild animals that likely modulates the zoonotic transmission risk.

Analysis of wild animal populations also emphasizes that individuals differ in their immune state [1,3,10]. Such interindividual heterogeneity could be due to genetic and/or environmental effects and/or their interaction, but the nature of the specific environmental factors that drive and constrain wild animals' immune systems is not known. Identifying these factors and how they modulate the immune responses (and so the immune state) of wild mice is the purpose of the work we present here.

Among wild animals (principally mammals and birds) there are well-described seasonal effects on measures of individuals' immune state, but these patterns differ among studies. For example, some measures of immune state increase in winter, whereas others decline [11–13]. Notwithstanding this variation, these seasonal patterns may be driven by a number of different (and potentially interacting) factors, including hormonal differences (for example, due to photoperiod-driven effects per se, and/or seasonal reproduction), resource availability, and the force of infections [11–14]. Animals' resource availability has been shown to affect immune responses in both laboratory and wild animals, consistent with the idea that these responses are costly and become compromised when resources are limiting [15,16]. Age has a significant deleterious effect on immune responses and immune state (described as immunosenescence) in laboratory animals and in humans [17,18], though for wild mammals this has only been shown in wild Soay sheep [13,19–21]. For wild mammals more generally, both the difficulty of determining wild animals' age and that wild animals may lead shorter lives than laboratory animals [1] may make detecting age-related effects challenging. Collectively, these studies point to the effects that a range of different factors can have on animals’ immune responses and on immune state. However, what these previous studies have not considered is the relative magnitude, importance, and interaction of these different factors acting on wild animals’ immune state. Moreover, our immune system-wide analysis of laboratory and wild mice demonstrated that the various compartments of the immune system differ in very different ways [1], meaning that analysis of just one or a few immune parameters will underrepresent an individual animal’s immune state and underestimate differences among animals or between populations. By necessity, many previous studies of wild animals have used just one, or a few, immune measures, with these sampled in different ways (for example, peripherally or systemically); the disparate conclusions drawn from different studies of wild animal immune systems is likely due, at least in part, to the use of different immune parameters.

Here we analyse in detail the immune state of populations of wild *M*. *musculus domesticus* in the southern United Kingdom. We have studied *M*. *musculus domesticus* as an example of a wild mammal population, and one that is ideal for this because it is the same subspecies as the laboratory mouse [22], making the plethora of laboratory mouse immunological tools available. *M*. *musculus domesticus* live commensally, typically in farm outbuildings, within which there is deme-based breeding centred around a dominant male [23,24]. These populations are regulated by the same top-down and bottom-up ecological processes that regulate populations of fully free-living species, and so, critically, the immune state of these commensal animals will be subject to similar drivers and constraints as are other wild species. Commensal mice have limited migration between sites, with dispersal typically undertaken by young males, together making populations relatively stable, which facilitates sampling [25]. We find that local *M*. *musculus domesticus* populations differ immunologically and that these populations are genetically distinct, but that genetic differences among populations do not contribute to the populations' immunological differences. We then identify the principal factors that underlie the immunological differences among mice, showing that body condition promotes and age constrains individuals’ immune state. In female mice, these factors interact such that although age constrains immune state, improving body condition with age leads to an overall improvement in immune state with increasing age. In male mice, these 2 parameters act independently. Thus, this work demonstrates that detailed immunological analyses of wild populations can reveal extensive heterogeneity of immune state among wild animals and can identify the key factors affecting this. Importantly, our study reveals that host intrinsic factors such as body condition and age are much more important drivers of immune state than genetic background, microparasite infection, or extrinsic factors such as season. These results emphasise that understanding wild animals' immune systems is necessary to fully understand the biology of wild animal populations, as well as that of their pathogens.

## Results

### Wild mouse populations differ immunologically

**Fig 1:**
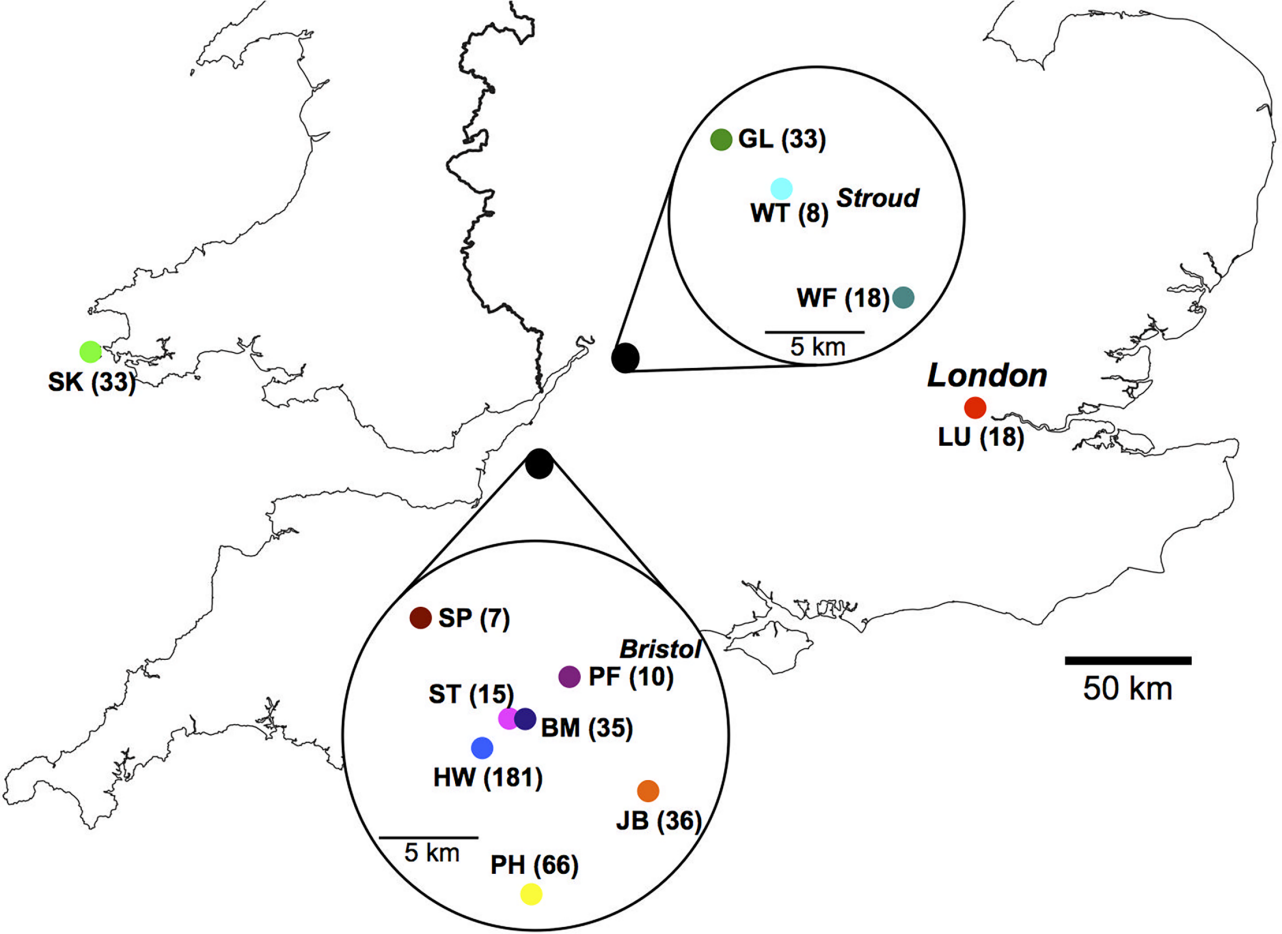
Wild mice were sampled from across the southern UK. The 12 sampling sites are shown by 2-letter designations, with the number of animals obtained at each site shown in parentheses.

We sampled 460 house mice, *M*. *musculus domesticus*, from 12 sites in the southern UK (Fig 1; S1 Table). The sex ratio was 1 female to 1.18 male, and the median age was 7.4 weeks, with 75% of mice being 12 weeks of age or younger (S1 Fig). The mice had a limited macroparasite fauna, consisting primarily of *Syphacia* sp. pinworm nematodes (prevalence 79%) and *Myocoptes musculinus* mites (prevalence 67%; S1 Data). We tested mice for seropositivity to 7 microbial infections (Noro, Minute, Parvo, Sendai, Corona, and Mouse Hepatitis viruses, and *Mycoplasma pulmonis*), finding a high seroprevalence (95%) of one or more of these microparasite infections, consistent with previous reports [26,27]. We detected antibodies to Sendai virus in these populations, which, to our knowledge, is its first report in wild mice (S1 Data). The number of these microbial infections accumulated as mice aged (number of infections and age *r* = 0.27 and 0.3, *p* < 0.0001, *n* = 214 and 209 for males and females, respectively, S2 Data).

For each mouse, we made a large number of immune measures, using a cross-sectional study design (S1 Text) [1]; here we focus on the (1) serum concentration of immunoglobulin (Ig) G and IgE and acute phase proteins (serum amyloid P and haptoglobin), (2) faecal concentrations of IgA, and (3) numbers, proportions, and ex vivo activation status of splenic T and B cells, regulatory T cells (T_regs_), NK cells, dendritic cells (DCs), and myeloid cells. Although there may be short-term perturbations to individuals’ immune state that would not be captured by this cross-sectional approach, studies in voles, *Microtus agrestis*, and humans have shown that over the longer term, immune parameters are largely temporally stable [28,29], suggesting that our data are generally representative of an individual’s immune state.

Because we sampled mice from 12 sites, many of which were geographically clustered (Fig 1), we first wanted to understand the extent of immunological diversity among these sites. We did this by first using a principal component analysis of our immunological data, revealing 3 components: component 1 (accounting for 53% of the variation) comprised 7 cell populations (scaled number of CD4^+^ and CD8^+^ T cells, B cells, NK cells, neutrophils, DCs, and macrophages), component 2 (13% of the variation) comprised the serum concentration of IgG and IgE, and component 3 (10% of the variation) was the faecal concentration of IgA (S3 Data). We then calculated the immunological distance among all individual mice [28] as the pairwise distance among mice of these 3 principal components, and then compared these distances within sample sites and among sample sites.

**Fig 2:**
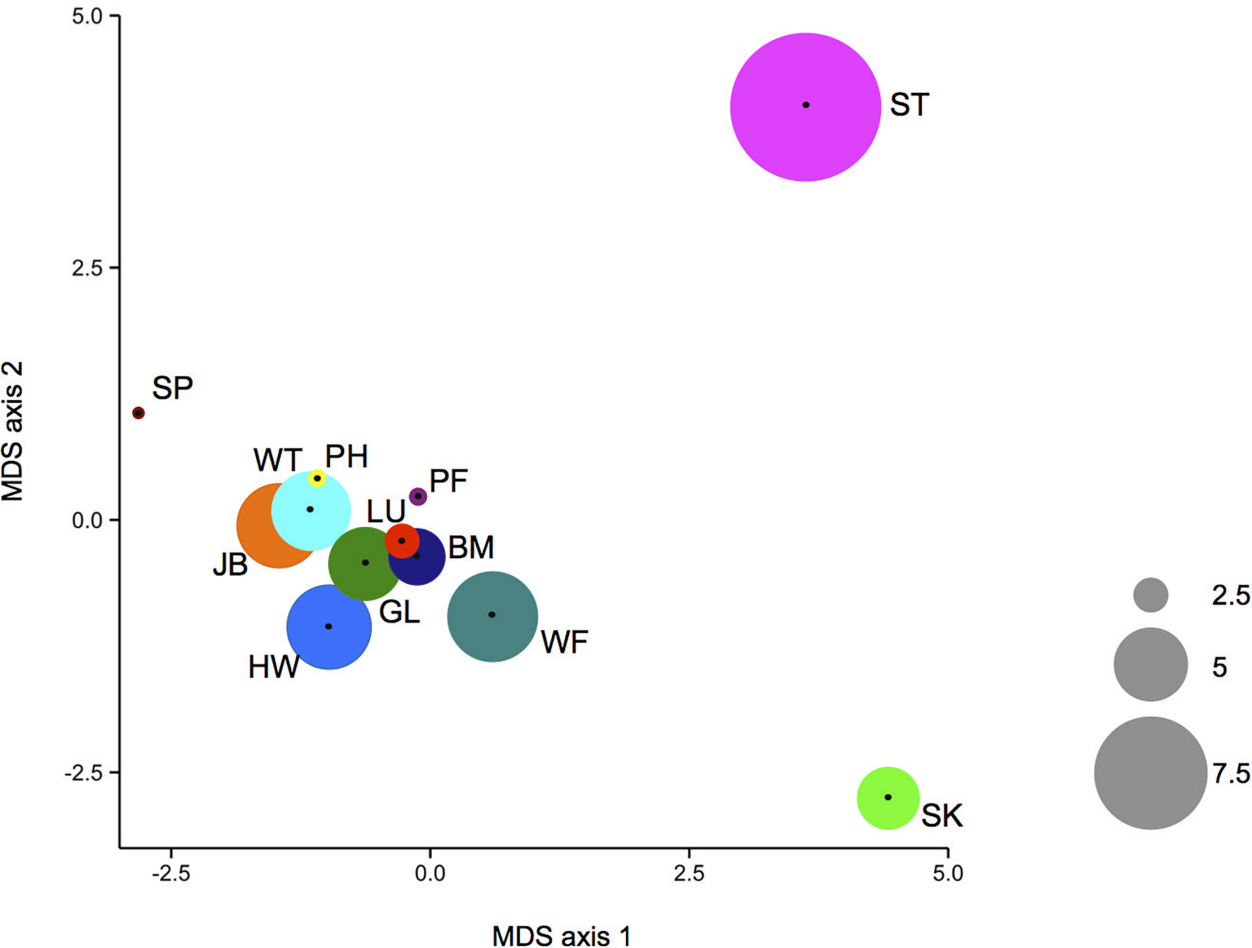
Mice vary in their immunological distance. The immunological distance among mice (i) within each sample site is shown as the size of the coloured circles, and the immune distance among sites is shown as the 2-dimensional immunological distance following multidimensional scaling (MDS). Within sample site and among sample sites are therefore shown on different scales. Sample sites are colour coded, and 2-letter designations are as shown in Fig 1.

The results showed variation in immune state of mice both within and among sites. Overall, the mean among-site immunological distance was greater than the mean within-site distance (4.7 ± 0.197 versus 3.8 ± 0.096, mean ± SE; Fig 2, S2 Table), meaning that mice at one site are immunologically more similar to each other than they are to mice from other sites. The immune similarity of mice within each site differs among sites. Within some sites, mice are immunologically very similar, whereas at other sites they are comparatively diverse. For example, mice within sites PF and PH are very homogeneous (immune distance 1.21 ± 0.14, 1.43 ± 0.019, mean ± SE), while mice within sites ST and HW are more diverse (immune distance 9.64 ± 2.3 and 5.68 ± 0.21, respectively). The immunological differences among sites can operate on a very local geographical scale. For example, mice at site ST are comparatively highly immunologically diverse (immune distance 9.67 ± 2.33), and consistently distinct from mice at 5 neighbouring sites (BM, HW, PF, SP, and JB; Fig 1, S1 and S2 Tables) that were 0.2–8 (mean 4.3) km distant. While there have been some observations of differences in specific immunological parameters among rodent populations (e.g. [29,30]), this is the first demonstration, of which we are aware, of the structure of immune variation within and among different wild animal populations.

### Wild mice are genetically diverse

**Fig 3:**
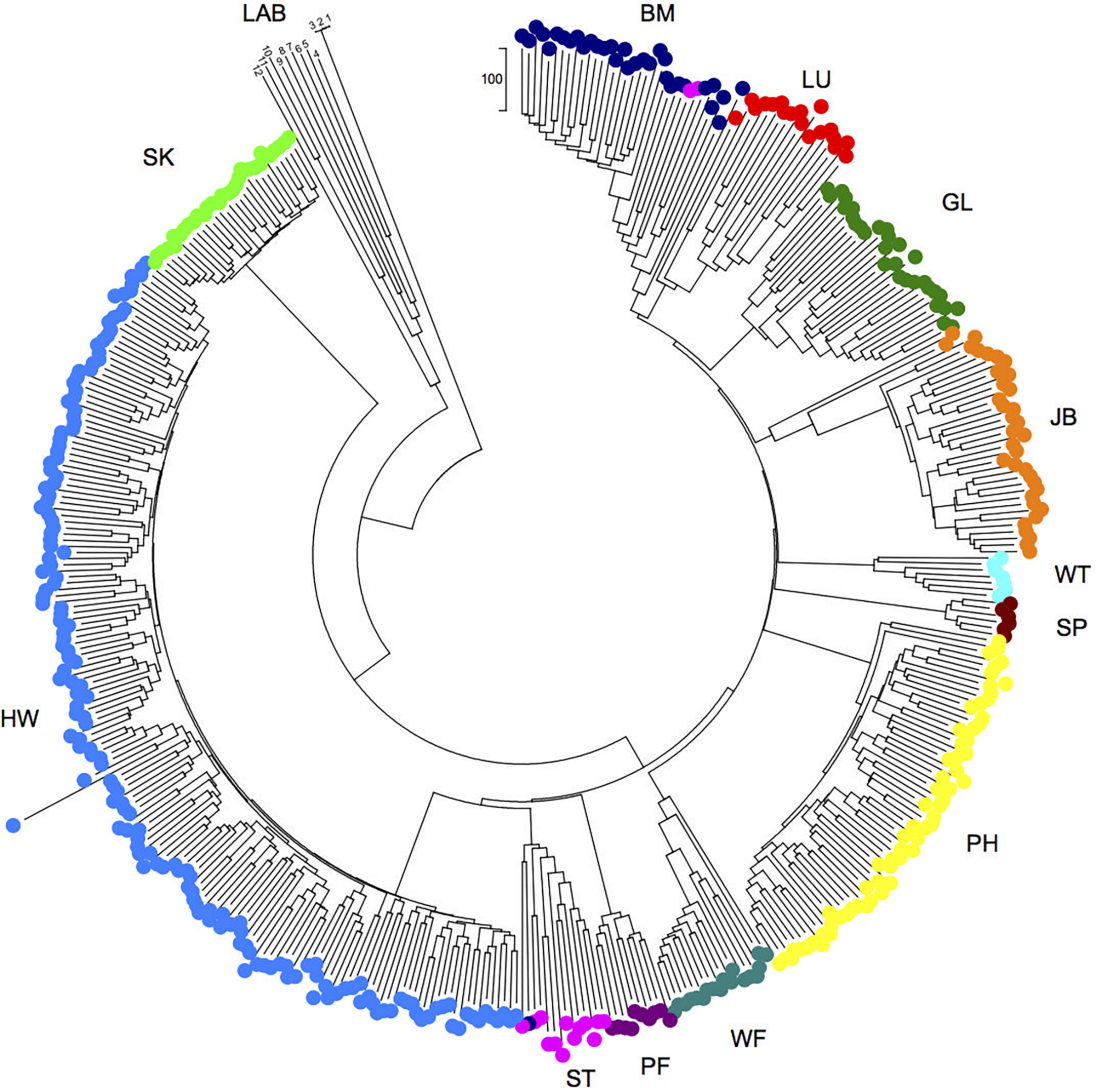
Mice have strongly genetically structured populations. A neighbour-joining tree showing the relationship among mice. The site colour coding and 2-letter site designations are as Fig 1. The scale is the number of nucleotide differences among individuals. Mice numbers 1–12 are control laboratory mice (LAB), where 1 and 2 are L88 and L90 C57BL/6 mice as in [1], 3 is C57/BL6J, 4 is SJL/J, 5 is FVB/NJ, 6 is NOD/LJ, 7 is BALB/cJ, 8 is AKR/J, 9 is DBA/J, 10 is C3H/HeJ, 11 is CBA/J, and 12 is 129S1/SvlmJ and where 3–12, inclusive, are data obtained from http://support.illumina.com/array/array_kits/mouse_md_linkage/downloads.html.

In view of the immunological structure of these wild mouse populations, we next investigated their genetic structure. To do this, we genotyped the mice at 1,183 neutral autosomal loci and used this to calculate the genetic distance among individuals (Fig 3, S4 Data), the fixation index, F_ST_, and the inbreeding coefficient, F_IS_. This showed that there was strong genetic differentiation among populations from the 12 sites, seen as mice from each site being genetically more similar to each other than to mice from other sites (Fig 3). This was confirmed by STRUCTURE analyses showing that the most likely number of genetic clusters was 9, due to mice at some of the sites being genetically more closely related (S2 Fig). The F_ST_ values for the wild mice are among the highest described for a wild mammal, with the average F_ST_ = 0.48, while the average F_IS_ = 0.00052 (Fig 3, S3 Table, S2 Fig), suggesting that there is limited effective movement of individuals among the sites.

**Fig 4:**
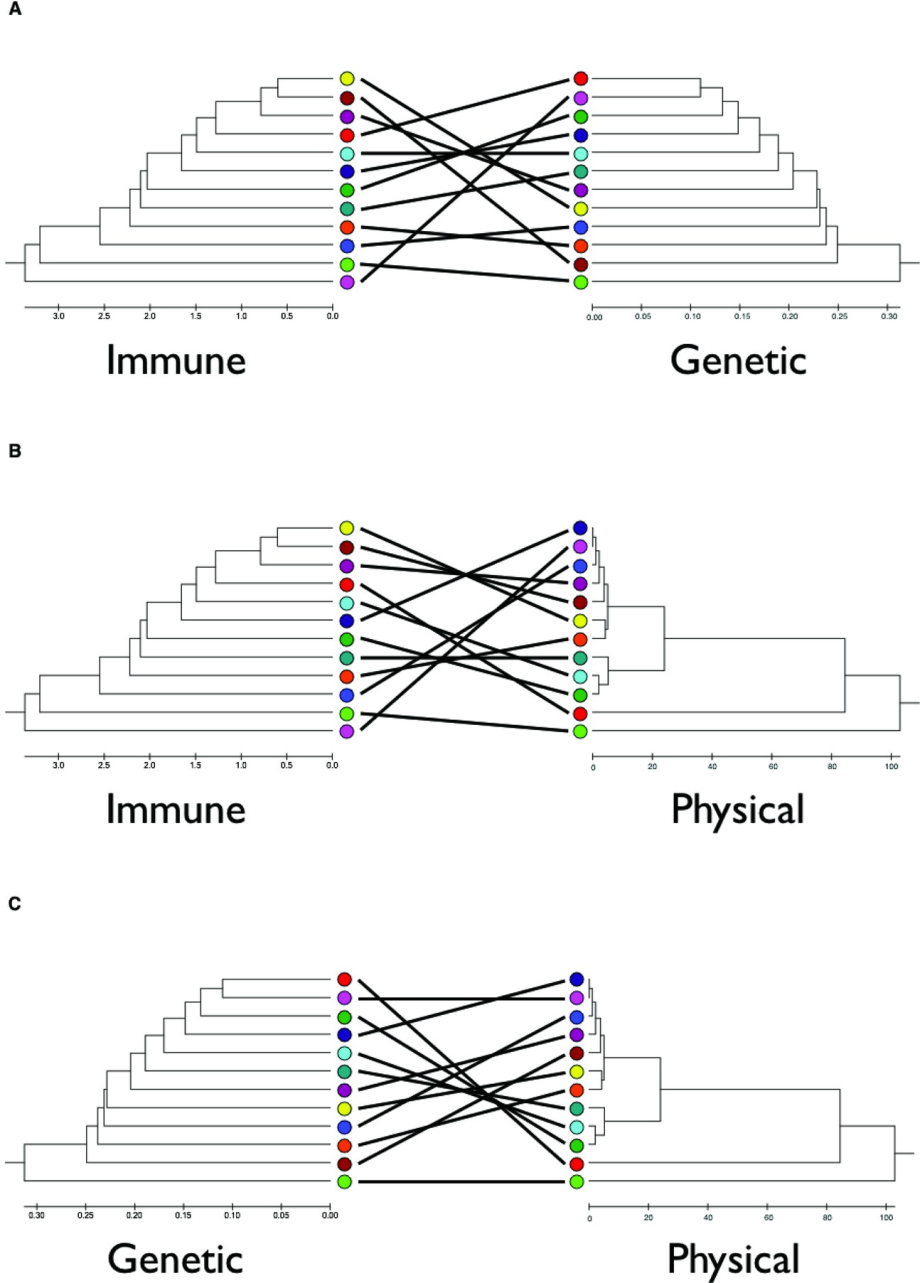
Immunological distance is not explained by genetic or physical distance. Tanglegrams based on unweighted pair group method with arithmetic mean (UPGMA) trees of immunological distance, genetic distance, as F_st_, and geographical distance among sites, where the scales are the relevant measures (S1, S2 and S3 Tables) of (A) immunological and genetic, (B) immunological and geographical, and (C) genetic and geographical distance among mice from the different sites. The site colour coding is as in Fig 1.

The local population genetic structure is not driven by geographical distance (F_ST_ and geographical distance among sites do not correlate; Fig 4, S1 and S3 Tables), as previously seen for this species [23]. For example, sites separated by only 2.3 km (sites BM and HW) have F_ST_ values equal to sites separated by more than 50 km (sites GL and HW) (F_ST_ = 0.47 and 0.45, respectively). Because it is an island, mice on Skokholm are, presumably, a relatively isolated population. Commensal mice have limited migration [25], and not all individuals breed [24,25], probably contributing to the population genetic structure we observe [23]. These effects are likely exacerbated by episodes of pest control resulting in bottlenecks or local extinction, which is followed by recolonization (often anthropogenically [25]), with consequent genetic founder effects. Together, these processes will contribute to the generation of these highly genetically structured populations of *M*. *musculus domesticus*.

Importantly, there is no relationship between immunological distance among sites and genetic (Fig 4, *r* = 0.087, *p* = 0.382) or geographical distance (Fig 4, *r* = 0.192, *p* = 0.264 and *r* = 0.082, *p* = 0.339 for distance and log(distance + 1), respectively) (S2 and S3 Tables). This means that immunological differences among populations must be driven by other factors. While genotyping at this number of loci provides good coverage of the mouse genome, not all genetic variation will be captured and, for example, effects of loci of strong immunological effect that are unlinked to any of these markers will not be accounted for. With this caveat, these results strongly suggest that the immunological structure of wild mouse populations is driven by factors other than the genetic background or geographical structure of the mouse populations.

### Condition promotes, and age constrains, wild mouse immune state

To identify the other factors that drive, or constrain, immune state in these populations and to explain the differences among individual mice in their immune state, we used structural equation modelling (SEM), in which causal relationships among potential factors are specified and then tested against empirical data to generate quantitative, causal conclusions. We explored a range of different models containing a wide range of factors (S1 Text, S4 and S5 Tables, S3 and S4 Figs). Here we present the model that was the most parsimonious, inclusive, and robust among those that we tested. This model assigns causal relationships among a number of factors acting on the latent variable of Immune State (S5 Fig). We considered factors previously implicated in affecting immune state in wild populations [11,12,13,16–18,31–33], focusing particularly on Season (measured as day length), Age (calculated from eye lens mass [34]), Body Condition (measured as the scaled mass index [SMI] [35]), and microparasite Infection (measured as the number of microbial infections). Each of these factors have been shown individually to affect the immune systems of animals, but how they act and interact in wild, free-living populations is unknown.

Body condition has repeatedly been implicated in affecting immune responses and state, but it can be measured in various ways [33]. We used SMI that calculates the scaling exponent of body mass and body size for the population and then used this to scale each animal’s body mass to that of the average body length of the population [35]. In wild mice, SMI correlates strongly with body mass index (BMI) (males *r* = 0.88, *p <* 0.0001, *n* = 247; females *r* = 0.83, *p <* 0.0001, *n* = 211, respectively, at all sites; S2 Data) and also correlates with abdominal fat mass (males *r* = 0.13, *p* = 0.038, *n* = 244; females *r* = 0.25, *p* < 0.0001, *n* = 188; S2 Data), although correlations with serum leptin concentration are only significant in females (*r* = 0.24, *p =* 0.01, *n* = 117; S2 Data). This is consistent with studies in laboratory mice and humans, finding a relationship between BMI and the concentration of leptin, but a poor correlation between leptin and the mass of fat [36]. Despite these correlations, we find that different measures of body condition are not all always equivalent in their relationship to various immune parameters (S2 and S5 Data), emphasizing the importance of comprehensively assessing and understanding animals' body condition [33,35].

In the SEM analysis, because of the heterogeneity in immune state among the different sites, we focused on the HW site, which was deeply sampled and where the results were broadly representative of all sampled mice (Fig 2, S1 Text); SEM results for the full data set are shown in S6 Fig and S6 Table. The SEM analyses considered different immune components in turn, because in preliminary analyses conglomeration of all these immune measures was not tractable within the structural equation models (S1 Text). The 3 immune compartments we used were the latent variables (1) adaptive cellular immune state (scaled numbers of CD4^+^ and CD8^+^ T cells, and CD19^+^ B cells), (2) innate cellular immune state (scaled numbers of NKp46^+^ NK cells, Ly6G^+^ neutrophils, CD11c^+^ DCs, and F4/80^+^ macrophages), and (3) humoral immune state (serum IgG and IgE, and faecal IgA concentration). Importantly, these 3 immune compartments were each immunologically coherent.

**Fig 5:**
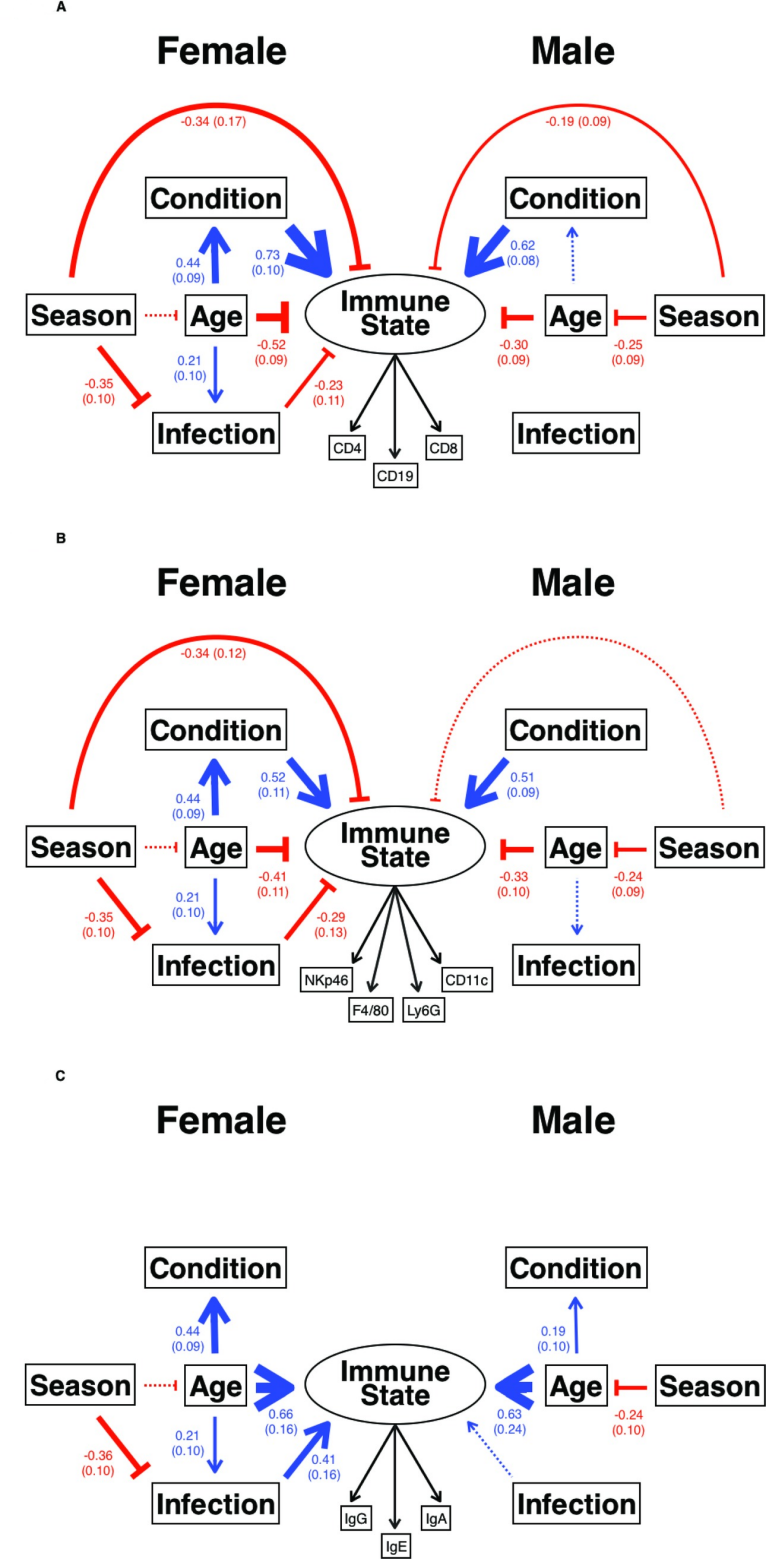
The principal drivers of immune state in wild mice. How (A) adaptive cellular, (B) innate cellular, and (C) adaptive humoral Immune State are affected by Season (measured as day length), Body Condition (measured as the scaled mass index), Age in weeks, and Infection with up to 7 microbial infections, with latent variables shown as circles and observed variables shown as boxes, and where blue arrows show positive effects, red blunt-ended lines show negative effects, and line thickness indicates the size of the covariance, which is shown (with the SE in parentheses) for mice from site HW; marginally nonsignificant results are shown by thin dotted lines. All estimates, SE, and *p*-values are shown in S7 Table. In (A), for females root mean square error of approximation (RMSEA) = 0.0 (0.0–0.126), comparative fit index (CFI) = 1.0, standardized root mean square residual (SRMR) = 0.03, χ^2^ = 7.64, df = 8, *p =* 0.469, for males RMSEA = 0.058 (0.0–0.139), CFI = 0.987, SRMR = 0.031, χ^2^ = 10.65, df = 8, *p =* 0.22; (B) for females RMSEA = 0.0 (0.0–0.08), CFI = 1.0, SRMR = 0.045, χ^2^ = 10.39, df = 14, *p =* 0.732, for males RMSEA = 0.137 (0.089–0.188), CFI = 0.891, SRMR = 0.082, χ^2^ = 40.46, df = 14, *p =* 0.0002, which is not a significantly good fit; (C) for females RMSEA = 0.093 (0.0–0.176), CFI = 0.926, SRMR = 0.09, χ^2^ = 13.64, df = 8, *p =* 0.0917, with warnings concerning the latent variable Immune State, for males RMSEA = 0.0 (0.0–0.093), CFI = 1.0, SRMR = 0.058, χ^2^ = 5.74, df = 8, *p =* 0.675.

The structural equation models revealed that the adaptive and innate cellular compartments of the immune system are principally affected by body condition (Fig 5, S7 Table; and correlation of SMI and 7 cell populations in males, *r* = 0.26–0.64, *p* ≤ 0.035, *n* ≥ 65; correlation of SMI and 6 cell populations in females, *r* = 0.30–0.49, *p* ≤ 0.033, *n* > 49; S5 Data) and are constrained by age (Fig 5, S7 Table). In females, these effects are linked because although age has a direct negative effect on the immune system (Fig 5, S7 Table; and correlation of age and 4 cellular populations *r* = −0.28 to −0.32, *p* ≤ 0.049, *n* ≥ 48; S5 Data), as females age their body condition improves, which enhances their overall immune state (Fig 5; and correlation of SMI and age in females *r* = 0.46, *p* < 0.001, *n* = 79; S5 Data). In contrast, in males, age and body condition operate more independently on adaptive and innate cellular compartments (Fig 5), since the correlation of SMI and age is weaker in males than in females (correlation of SMI and age in males *r* = 0.24, *p* = 0.019, *n* = 99, S5 Data). In males, for adaptive cellular immune state there is a marginally nonsignificant effect of age on condition (0.19, 0.099 [Estimate, SE], *p* = 0.051; S7 Table). The age-related decline in immune state is suggestive of immunosenescence [17,18], and this is particularly notable given the short lives (median age 7.4 weeks, S1 Fig) of these wild animals. Adaptive cellular state and innate cellular state were negatively affected by season in both male and female mice, with the effects stronger in females than in males (Fig 5). For innate cellular state in males, this effect is marginally nonsignificant (−0.19, 0.1 [Estimate, SE], *p* = 0.064; S7 Table). Although the immune state of these wild mice is very likely driven—at least in part—by immune responses to microparasite infection [1], the infection parameters assessed here explained very little of the difference among individuals in their immune state. This may be because our primary measure of microparasite infection was serological and thus reflective of historical infection, which would tend to minimize effects on current cellular immune state or, for example, an animal’s condition.

Interestingly, the humoral immune compartment is affected very differently by these factors. Antibody concentrations increase with age in both sexes (Fig 5, S7 Table; and correlation of age and IgG, E, and A in females *r* = 0.35–0.49, *p* ≤ 0.002, *n* ≥ 44; correlation of age and IgE in males *r* = 0.32, *p* = 0.002, *n* = 95, S5 Data). Because antibodies persist, their increasing concentration with age is likely to reflect age-associated accumulated exposure to microparasite infection. Body condition has no effect on the humoral immune compartment, but microparasite infection does (Fig 5); microparasite infection is significant in females and shows a similar trend in males (0.39, 0.21 [Estimate, SE], *p* = 0.055, S7 Table).

Comparing the results for the HW site alone with the results for all sites together shows some coherence in the results, but not identity. Thus, for both innate and adaptive cellular immune compartments, the positive effects of body condition and the negative effects of age are qualitatively consistent; effects of season on immune state are largely consistent (but differ between these compartments), while effects of microparasite infection are more variable. For humoral immune responses, the effects of age and microparasite infection are also qualitatively consistent between the HW site and all sites together. Differences among our sample sites in how these extrinsic and intrinsic factors can affect the immune state of wild mice are, in principle, consistent with our observations of immune variation among different populations (Fig 2).

## Discussion

We have undertaken an in-depth, immune system-wide analysis of the immune state of wild house mice and used a multifactorial analysis of factors potentially affecting this to understand what actually drives and constrains immune state in the wild, and so why animals differ in their immune state. We have, firstly, shown how immune variation is structured among different populations of wild mice, which is the first time that the structure of immune variation in a wild mammal has been described. We find that mice within a sample site are more immunologically similar to each other than they are to mice from other sites, which is broadly equivalent to the recently reported situation in humans where cohabitation of unrelated individuals explains a very significant amount of the interindividual immune variation [28]. Individual wild field voles, *M*. *agrestis*, have been shown to consistently differ in the expression of genes whose products have immunological function, suggesting that there are long-term, individual-specific immune responses [29] and that such effects may contribute to population-level patterns such as we observe for *M*. *musculus domesticus*. If this pattern of local, population-specific immune differences is generalised to other wild animal populations this could have very important consequences. For example, for pathogens that are subject to immune-dependent selection, this immune heterogeneity may drive different evolutionary trajectories of the pathogens in different populations of hosts. The potential heterogeneity of the immune landscape in which pathogens live and evolve is not usually considered in studies of pathogen evolution. However, the temporal stability of the immunological patterns we observe, both within and among sites, remains to be investigated in longitudinal studies. The possibility of rapid changes in the immune landscape could bring further complexity to the environment and the selection pressures that pathogens experience. More broadly, our results suggest that the health and disease status of wild animals, as well as their potential resistance to new infections, need to be considered at a local population scale. The causes of this immunological structure—which are likely to be multifactorial—are not yet known, and so investigating this is a priority. Work with human populations has shown that carefully designed studies for this specific purpose are required to unpick these effects [37].

Our second key finding is that cellular immune state (both innate and adaptive) is principally affected (positively) by animals' body condition and (negatively) by their age. In females these factors interact so that as females age, their body condition improves, improving their immune state. In males these 2 factors are less interconnected. Together, this means that the highly activated state of wild mouse immune systems [1] is underpinned by good body condition, but this is also a state that can rapidly senesce. Body condition is also likely to underpin many other important life history traits, such as reproductive success, meaning that there will be competing demands for resources among these different traits. The concept of body condition is widely used, aiming to capture an idea of an animal’s ‘plumpness’ [38]. However, there are different definitions and measures of body condition, and these different measures do not always correlate [39]. We used SMI as our principal measure of body condition and found that it correlated with BMI and with the mass of abdominal fat (but not with the serum concentration of leptin), thus suggesting that in this study at least, body condition represents an animal’s overall body reserves. Given that wild animals are likely to have temporally heterogeneous access to food, it will be important to understand the temporal variability in individuals’ body condition and its relationship to the dynamics with which fat deposits can be converted into available energy. Season and microparasite infection explained rather little of the variation in immune state among individual animals. The absence of any substantial effect of season may be due, at least in part, to the commensal lifestyle of these mice, which might buffer them from typical natural environmental fluctuations. Additionally, complex effects of season may not be adequately captured by day length, the measure of season that we used. Similarly, while infection likely explains the highly activated immune state of wild mice compared with laboratory mice [1], our measures of infection do not explain the immunological heterogeneity among wild mice. This may be because our measures of microparasite infection were mostly serological and therefore represent historical infection; this is also why our SEM did not allow an interaction from immune state to infection. In undertaking these SEM analyses we explored a large range of potential models, which included diverse immunological and other physiological parameters. Our modelling approach also considered data obtained from site HW alone and from all sample sites combined. While the results from the 2 approaches were broadly equivalent, they are not identical. This discordance likely points to different local ecologies, population demographies, and infections at the different sites, all of which will affect the qualitative and quantitative nature of the factors that both drive and constrain animals’ immune state. Our observation of how immune variation is structured among the populations that we have sampled is also consistent with this view. We explored many ways in which measures of animals’ immune state could be incorporated into our models. The approach we favoured (modelling 3 immune compartments separately) is probably not the only approach that could be used. However, our extended analyses of alternative approaches points to the difficulty of capturing ‘immune state’ in a meaningful way in the face of multiple humoral and cellular measures. Overall, the results of our SEM analyses show the differing effects of intrinsic and extrinsic factors on the different immune compartments, suggesting the likely diversity of underlying selective pressures acting on these animals in optimizing their immune responses [40].

The analyses of the effects of these extrinsic and intrinsic factors also emphasize that it is critical to give careful thought to which immune compartments are sampled and how individual parameters are assayed in any given situation to properly and fully understand immune state. While we have made very extensive measurements of the immune state of splenocytes, together with analysis of antibodies and other serum proteins [1], there are numerous additional measurements that could be made, including from other immune compartments (in particular from mucosal sites that are major portals of pathogen entry), which may be particularly relevant to wild animals. Nevertheless, by using a multifactorial analysis we have been able to understand how different factors act and interact in affecting immune state, and for the first time been able to provide a unified understanding of what drives and constrains this in wild animals. While the mice we studied live commensally in farm buildings, they are subject to the same range of ecological processes as entirely wild animals living independently of humans—for example, top-down and bottom-up regulation, due to density-dependent limitation of resources and predation, and disease-related processes—and we therefore expect that the immune systems of such free-living species will be affected similarly. The commensal mice in this study had a very much more limited macroparasite fauna than has been reported for other species of wild rodents, indicating that the dominant pathogen challenge of wild *M*. *musculus domesticus* is provided by bacteria and viruses. However, bacteria and viruses probably provide the bulk of the antigenic challenge in most animals, irrespective of their macroparasite fauna. Overall, therefore, we believe that house mice represent both a valid and a tractable example of wild mammal populations and reveal important, likely generalizable, interactions between environment, physiology, and immunology. However, the key next step is to extend this approach to other species, to understand the common factors that support and constrain immune systems and those that differ among species. Trapping, handling, and sampling wild animals is unavoidably highly stressful for them, and will undoubtedly affect some (possibly many) of the immune parameters that are measured; this needs to be borne in mind when interpreting such data and poses considerable challenges for the prosecution of longitudinal studies. Technological developments that allow remote monitoring of key physiological parameters in free-living animals may help to overcome this constraint. Nevertheless, understanding the extrinsic and intrinsic factors that affect wild animals' immune systems gives us a rational way to understand how such perturbations may affect their immune responses and immune state and thus their health and disease, and also what can be done to ameliorate these effects.

This study shows the feasibility of analysing in detail the immune state of wild animals (if suitable immunological tools are available) so as to begin to understand how the immune system contributes to individuals’ fitness in the wild. The immune heterogeneity that we have observed among different populations begs the question of the relative roles of host biology, antigenic challenge, and other environmental factors in supporting or constraining immune state at the local level.

## Methods

### Ethics statement

This work was reviewed and approved by the University of Bristol's Animal Welfare and Ethical Review Board as UB/15/055. Trapping and killing nonprotected wild animals is not encompassed by the Animals (Scientific Procedures) Act 1986.

### Mice

The mice used in this study were trapped between March 2012 and April 2014 at 12 locations (S1 Table, S6 Data), principally within a 30-mile radius of Bristol, UK; the 2 exceptions were the London Underground system and Skokholm island, southwest Wales. We surveyed many sites for the presence of mice and trapped at each site where mice were found. We visited each site, in succession, every season, although our trapping success was dependent both on site access on the day and the continuing presence of mice, which was variable. Inevitably, therefore, dates when mice were trapped are confounded with site. Mice were trapped with Longworth traps (Pennon, UK) baited with oats, raw carrot, and hay bedding and checked at least once every 24 hours. Once caught, mice were transferred to a conventional animal house, where they were weighed before being individually housed and fed on a commercially available rodent diet ad libitum (EURodent diet 22%; PMI Nutrition International, Brentwood, Missouri, United States) for an average of 5.5 (SD = 3.8) days before they were killed (S6 Data) [41]. This allowed us to process mice in batches with relevant internal controls, which were C57/BL6 laboratory mice [1]. There were no correlations between the number of days a mouse was housed in the lab and the immune measures, the concentration of IgG, IgA, IgE, the proportion of splenocytes that were CD3^+^ T cells, CD4^+^ T helper cells, CD49^+^CD4^+^ activated T helper cells, CD8^+^ T cytotoxic cells, CD49^+^CD8^+^ activated T cytotoxic cells, KLRG1^+^CD8^+^ terminally differentiated T cytotoxic cells, NKp46^+^ NK cells, CD19^+^ B cells, mean fluorescence intensity of PNA^+^CD19^+^ B cells, CD11c^+^ DCs, and F4/80^+^ macrophage cells. Mice caught on the London Underground were taken to the lab, where they were killed immediately and processed as described below. A total of 460 mice were captured and processed (S6 Data).

### Dissection

Mice were killed by intraperitoneal injection with an overdose of sodium pentobarbital, followed by cervical dislocation. Mice were weighed after death; female mice that were later found to be pregnant had the mass of the foetuses subtracted from their final mass, and these values were used in all subsequent analyses. Blood samples were taken by cardiac puncture, and the blood was stored in heparinized containers for no more than 1 hour prior to being centrifuged at 13,000 *g* at 18°C for 10 minutes. The resulting plasma was divided into aliquots and stored at −20°C. Spleens were removed aseptically, weighed, and transferred to 5 mL of cell culture media for further processing as previously described [3] to determine a viable spleen cell number.

For each mouse, the gut was removed and stored at −20°C, while the kidneys, liver, heart, and lungs were all removed and weighed prior to storage in 5 mL of formalin (10% v/v formal saline). The eyes were removed using forceps and stored in 2 mL of formalin. The eye lenses were later removed, dried, and weighed as previously described [33] and used to calculate the age of the mice. Mouse carcasses were then stored at −20°C prior to further measurement and dissection. After defrosting, body length was measured from the tip of the snout to the base of the tail. Fat from within the abdominal cavity was removed and weighed.

Skull length and width measurements were taken after the soft tissue had been mechanically removed. Skull length was measured from the tip of the nasal cavity to the base of the skull (the longest point), and the width was taken at the widest point perpendicular to this line, using callipers.

### Serology

The blood haemoglobin concentration was measured using a HemoCue Hb 201 analyser (HemoCue AB, Ängelholm, Sweden). The serum concentration of leptin was measured using a commercially available ELISA kit, following the manufacturer’s instructions (Insight Biotechnology, UK). Mice were examined for evidence of microbial infection using 2 immunocomb kits (Biogal Galed Labs, Israel), which detect antibodies to the Corona, Mouse Hepatitis, Sendai, Minute, Noro, and Parvo viruses and to *M*. *pulmonis*. These tests were scored on a 0 to 4 scale, where 0 is the absence of a response, and 1, 2, 3, and 4 are all seropositive results in increasing degrees of positivity. These microbial infections are likely to only represent a subset of all the microbial infections to which wild mice are exposed, many of which may be unknown infections.

### Parasitology

A visual inspection of each mouse was made as they were removed from the trap to detect large ectoparasites such as ticks and fleas; smaller ectoparasites, such as mites, were counted by visual inspection of the dead mice. The trap contents were also inspected. For each mouse, the total pelt (skin and fur) was examined under a dissecting microscope. Ectoparasite samples were removed for identification, and the number of mites observed was classified into the categories: 0–10 recorded as the actual number, 11–20, 21–30, 31–40, 41–50, 51–100, 101–200, 201–300, 301–400, 401–500, 501–1,000, 1,001–1,500, and 1,501–2,000. A sample of mites were mounted on glass slides using Hoyer’s medium and identified as *M*. *musculinus* by morphological examination.

To determine the intestinal nematode fauna, stored intestines (see above) were defrosted and slit longitudinally, the gut then examined using a dissecting microscope, and worms counted as described previously [41]. Detailed morphological examination of a sample of the worms identified them as nematode pinworms, *Syphacia* sp.

### Indices of condition

The SMI was calculated using the equation previously described [35], which determines the exponent of the relationship between body mass and body length in the population under study, and then uses this value to calculate the mass of each individual animal if it was average size. In this way, this identifies animals that have disproportionately large or small body masses. The BMI of each mouse was calculated as body mass in kg / (body length in m)^2^. For female mice that were found to be pregnant, the mass of their foetuses was subtracted from their final body mass.

### Immune cell populations and scaling

Counts of immune cell type were obtained using methods previously described [1], and these counts then scaled analogously as for the calculation of SMI (above), but replacing mouse mass with the count for each cell type measured. The relevant scaling component was calculated separately for each of the relevant cell types. This results in the count of each immune cell for each mouse scaled to the length of the average mouse. We used this scaling method for the following cell types: CD4^+^ T cells, CD8^+^ T cells, CD19^+^ B cells, NKp46^+^ NK cells, Ly6G^+^ neutrophils, CD11c^+^ DCs, and F4/80^+^ macrophages [1]. Scaling the immune cells in this manner, rather than using a proportion of each cell type, allowed us to use count data in our modelling while still controlling for effects of mouse body size.

### Genotyping and population genetic analyses

Mice were genotyped at 1,183 autosomal loci as described in [1]. In total, 445 mice were genotyped: 443 wild mice and 2 laboratory C57BL/6 mice (L88 female and L90 male), as in [1]. The neutrality of the loci was tested using the Bayescan program [42], which detected 15 loci that were unlikely to be neutral and these were removed from further analyses, leaving data for 1,168 loci.

We used Arlequin to calculate Hardy-Weinberg expected measures of heterozygosity and tested for deviations from Hardy-Weinberg equilibrium using the Markov chain exact test [43] with 1 million iterations. Initial analysis identified many loci as significantly deviating from Hardy-Weinberg equilibrium, suggesting the existence of the Whalund effect, and therefore, a repeat of these analyses on a per sample-site basis was conducted, resulting in far fewer loci being found to deviate from Hardy-Weinberg equilibrium. We further tested for genetic subdivision among the mice using Wright’s F_ST_, calculated in Arlequin, using a pairwise method, where F_ST_ significance was determined through 100 repeated measures; F_IS_ was calculated similarly. We calculated the genetic distance among individual mice as the number of nucleotide differences and used this to construct a nearest-neighbour joining tree using 1,000 bootstraps, as in [1].

We also used STRUCTURE [44] to further investigate the population structure of the mice, which we did using the admixture model with correlated allele frequencies. This procedure assigns proportions of an individual’s genome to defined genetic clusters, K, and we used K values from 1–12 (with 12 being the number of sample sites, Fig 1), with 15 iterations (with 100,000 Markov Chain Monte Carlo iterations and a burn-in length of 50,000). Because of the unequal sample sizes among sites, we repeated these analyses using 6 randomly selected mice from each site, with 15 iterations, as above. We favoured the admixture model, recognizing that individual mice likely had some mixed ancestry. We also used Structure Harvester [45] to determine the posterior probability for the true value of K, and CLUMPPAK [46] was used to compile consensus results and to generate figures.

### Immunological distance

This was calculated as described by [28]. Briefly, we performed a principal component analysis of 10 immune measures: the scaled number of CD4^+^ T cells, CD8^+^ T cells, CD19^+^ B cells, NKp46^+^ NK cells, Ly6G^+^ neutrophils, CD11c^+^ DCs, F4/80^+^ macrophages, the serum concentration of IgG and IgE, and the faecal concentration of IgA. This generated 3 principal components representing all 7 cell populations; serum IgG, serum IgE; and faecal IgA (accounting for 53%, 13%, and 10% of the total variance, respectively) and from these we calculated the 3-principal component Euclidean pairwise distance among mice, which is the immunological distance [28], and multidimensional scaling of these data was used to plot them using R 3.2.2.

### Tanglegrams and Mantel tests

We constructed tanglegrams [47] of the immunological, genetic, and geographical distances (S1, S2, and S3 Tables) among sample sites (Fig 1) from UPGMA trees of each measure, which were calculated using MEGA6 [48], where the crossovers between trees were minimized. We used Mantel tests to test whether there were significant correlations between immunological distance and geographical distance and between immunological distance and genetic distance, using 999 randomisations to assess significance. Because geographical distance is skewed, we used both the actual geographical distance and log(distance + 1).

### SEM

We used an SEM approach to seek to understand how a range of extrinsic and intrinsic factors constrained and drove the immune state of wild mice. We used an iterative approach with respect both to the factors included in the models and to the model structures. This is explained more fully in S1 Text, S4 and S5 Tables, and S3 and S4 Figs. Based on these preliminary analyses, we divided our data by sex, and we also analysed all mice from all sites together, and for site HW only (Fig 1). The available sample size prevented random division of the data (were it possible, one-half of the data would be used for model construction while the other half of the data would be used for model validation). The results of these preliminary analyses led to the causal diagram shown in S5 Fig. In this way, we were able to assign directionality (and thus causality) to each pathway among the factors. This led to the structural equation model of the observed variables of Season, Age, Condition, and microparasite Infection, all in relation to the latent variable of Immune State. The model included all possible directional pathways (though because infection was measured serologically, thus representing historical infection, whereas condition was a contemporary measure, we only considered microparasite Infection affecting Body Condition and not vice versa). Season was the number of minutes of daylight, between sunrise and sunset, on the day that the mouse was caught, with these data obtained from the UK Meteorological Office (www.metoffice.gov.uk); we note that this minutes-of-daylight measure may not fully capture all seasonal aspects, for example, because days of equal length can occur in different seasons. Age was calculated from the eye lens mass as in [34]. Body condition was the SMI, calculated as in [35]. Infection was the number of microbial infections that were serologically detected. We used separate models for the latent variable of Immune State as (1) the adaptive cellular (scaled number of CD4^+^ and CD8^+^ T cells, and CD19^+^ B cells), (2) the innate cellular (scaled number of NKp46^+^ NK cells, Ly6G^+^ neutrophils, CD11c^+^ DCs, and F4/80^+^ macrophages), and (3) the humoral immune (concentration of serum IgG and IgE, and faecal IgA) state.

All variables were scaled by factors of 10 so that the majority of data were within the range of 1–10, as required for SEM analysis. Data do not need to be normally distributed for SEM analyses, but they do need to be linear, as our data were. Each model was run separately for female and male mice. The goodness of fit to the data for each model was assessed by the chi-square test of model fit (χ^2^), the root mean square error of approximation (RMSEA), the standardized root mean square residual (SRMR), and the comparative fit index (CFI). The results shown are of the standardized covariances since most variables were measured using different scales.

Nonsignificance (*p* > 0.05) for the χ^2^ goodness-of-fit test was used to indicate an acceptable fit of the model to the data, but our sample sizes make χ^2^ unreliable as a measure of goodness of fit. RMSEA and SRMR values less than 0.05 were accepted as a good fit of the model to the data; however, RMSEA values greater than 0.05 were accepted where the lower 90% confidence limit was 0.000. CFI values greater than 0.95 were accepted as a very good fit, and anything greater than 0.80 was considered as acceptable [49,50]. Occasionally, models ran, but with warnings about the fit of the model or the appropriateness of the data used. If these problems could not be resolved, the models were either discarded as unreliable or have been presented with the relevant warning message. All structural equation models were constructed using Mplus version 7.2 [49–51].

## Supporting information

S1 Data Infection data for all 12 sample sites, (A) for each mouse, where column A is the mouse ID number; column B is the site designation as in Fig 1; columns C–I, inclusive, are serological evidence of microbial infection on a 0 to 4 scale, where 0 is the absence of a response, and 1, 2, 3, and 4 are all seropositive results in increasing degrees of positivity; column J is the total number of microbial infections; and columns K and L are the intensity of mite (*M*. *pulmonis*) and nematode (*Syphacia* sp.) infections, respectively; ND, not done; (B) summarized for each sample site where columns B–K, inclusive, show the prevalence of the 9 infections at each site (column A); and columns L and M show the mean intensity of infection with mites and worms, respectively.
(XLSX)

S2 Data For (A) all 12 sample sites combined, and sample sites (B) GL, (C) PH, and (D) SK, correlations (2-tailed) among measures of infection, condition, and immune measures, with males above the diagonal and females below the diagonal. In all, cells are colour coded as red for *p* < 0.01 and green for 0.01 < *p* < 0.05. Daylight is a measure of the Season when mice were caught, specifically the number of minutes of daylight between sunrise and sunset; Age is mouse age in weeks; Mites is the number of mites, Microbe is the number of microbial infections; Worms is the number of *Syphacia* sp.; BMI and SMI are the body mass index and the scaled mass index, respectively; Ab Fat is the mass of abdominal fat; Leptin is its serum concentration; Haemoglobin is its concentration; immunoglobulin (Ig) G, IgE and IgA are the concentration of these antibodies as described in [1]; and the numbers of CD4^+^ T cells, CD8^+^ T cells, CD19^+^ B cells, NKp46^+^ natural killer (NK) cells, Ly6G^+^ neutrophils, CD11c^+^ dendritic cells (DCs), and F4/80^+^ macrophages, which have all been scaled (prefix ‘s’) as for SMI, all as described in the Methods. Note, in (D) sample site SK, the mice were not infected with worms (see S1 Data), and there was a too small sample size for measurement of IgA concentrations, shown as not done (ND).
(XLSX)

S3 Data (A) PCA analysis of cellular and humoral immune measures showing the first 3 principal components, where PC1 is the scaled number of 7 cell types (CD4^+^ T cells, CD8^+^ T cells, B cells, natural killer (NK) cells, neutrophils, dendritic cells (DCs), and macrophages, all scaled as for scaled mass index [SMI]), PC2 is the serum concentration of immunoglobulin (Ig) G and IgE, and PC3 is the faecal concentration of IgA. ID is mouse number, and site is where each mouse was caught, where 1 is BM, 2 is HW, 3 is LU, 4 is WF, 7 is GL, 8 is WT, 9 is PF, 10 is ST, 11 is JB, 12 is PH, 13 is SP, and 14 is SK, as in Fig 1. (B) The Eigenvalues and the percentage variance explained, and (C) the loadings of scaled (prefix ‘s’) number of CD19^+^ B cells, NKp46^+^ NK cells, CD8^+^ T cells, CD4^+^ T cells, F4/80^+^ macrophages, Ly6G^+^ neutrophils, CD11c^+^ DCs, and the concentration of IgE, IgG, and IgA.
(XLSX)

S4 Data Wild mouse multilocus genotypes showing the 1,168 locus genotypes of mice, where column A is the locus name, column B is the chromosome the locus is on, and column C is the position of the locus on the chromosome. Row 1 shows the source of the mice as S1 Table, and row 2 is the individual number of the wild mouse. The number of genotyped mice at each site is: HW 167, PH 63, JB 36, BM 33, GL 30, SK 30, WF 18, LU 18, ST 15, PF 10, WT 7, and SP 6. The genotypes of control laboratory mice are in [1].
(XLSX)

S5 Data For sample site HW, correlations (2-tailed) among measures of infection, condition, and immune responses, with males above the diagonal and females below the diagonal. Cells are colour coded as red for *p* < 0.01 and green for 0.01 < *p* < 0.05. Daylight is a measure of the Season when mice were caught, specifically the number of minutes of daylight between sunrise and sunset; Age is mouse age in weeks; Mites is the number of mites; Microbe is the number of microbial infections; Worms is the number of *Syphacia* sp.; BMI and SMI are the body mass index and the scaled mass index, respectively; Ab Fat is the mass of abdominal fat; Leptin is its serum concentration; Haemoglobin is its concentration; immunoglobulin (Ig) G, IgE and IgA are the concentration of these antibodies as described in [1]; and the numbers of CD4^+^ T cells, CD8^+^ T cells, CD19^+^ B cells, NKp46^+^ natural killer (NK) cells, Ly6G^+^ neutrophils, CD11c^+^ dendritic cells (DCs), and F4/80^+^ macrophages, which have all been scaled (prefix ‘s’) as for SMI, all as described in the Methods.
(XLSX)

S6 Data Physical characteristics of all mice, where column A is the mouse ID number; column B is the date the mouse was captured; and column C the date it was killed, with the days in captivity shown in column D; column E is the site where it was caught, where 1 is BM, 2 is HW, 3 is LU, 4 is WF, 7 is GL, 8 is WT, 9 is PF, 10 is ST, 11 is JB, 12 is PH, 13 is SP, and 14 is SK, as in Fig 1; column F is the number of minutes of daylight, between sunrise and sunset, on the day that mouse was caught; column G is its sex, where 1 is female and 2 is male; columns E–K, inclusive, are these sizes in mm; columns L–N, inclusive are these masses in g; column O are the number of these cells determined as described before [1]; columns P–S, inclusive, are these masses in g; column T is in mg; column U is in weeks calculated as described in the Methods; columns V and W are in g/L and ng/mL, respectively; and column X and Y are body mass index and scaled mass index, respectively, as described in the Methods. Blank cells are where these data are not available.
(XLSX)

S1 Fig The age distribution of mice from all sites. The median age is 7.4 weeks, the mean (±SD) is 10 (±8), and 75% of mice are ≤ 12 weeks old; *n* = 460 mice. Mice at the different sample sites differ by age (*H* = 35.882, *p* < 0.001), which post hoc tests show is due to site BM having older mice than sites HW (*p* = 0.017) and PF (*p* = 0.047).
(TIFF)

S2 Fig STRUCTURE analysis of mice for 1–12 clusters, K, (A) for all mice and (B) for a random selection of 6 mice from each sample site. In both, there were 15 iterations, and representative figures are shown. The sample site codes for the mice are: 1 = BM, 2 = GL, 3 = HW, 4 = JB, 5 = LU, 6 = PF, 7 = PH, 8 = SK, 9 = SP, 10 = ST, 11 = WF, 12 = WT. The colour order is the same in (A) and (B), where K1 = light blue, K2 = orange, K3 = purple, K4 = green, K5 = maroon, K6 = light pink, K7 = fuchsia, K8 = light green, K9 = dark yellow, K10 = khaki, K11 = brown, and K12 = light yellow. In (A), STRUCTURE harvester shows that K = 9 is the most likely value of K, and cluster resolution largely followed the order of decreasing sample size. In (B), the order in which clusters resolved was closer to the cluster F_ST_ values.
(PDF)

S3 Fig Class 1–3 structural equation models, where latent variables are shown as circles and observed variables are shown as boxes. Individual numbered models refer to S4 Table.
(PDF)

S4 Fig Class 4 structural equation models, where latent variables are shown as circles and observed variables are shown as boxes. Individual numbered models refer to S5 Table.
(PDF)

S5 Fig Full structural equation modelling (SEM) causal diagram, where the latent variable immune state can be adaptive cellular, innate cellular, or adaptive humoral immune state with season (measured as day length), body condition (measured as the scaled mass index), age in weeks, and infection with 7 microbial infections.
(TIFF)

S6 Fig The principal drivers of immune state in wild mice. How (A) adaptive cellular, (B) innate cellular, and (C) adaptive humoral immune state is affected by Season (measured as day length), Body Condition (measured as the scaled mass index), Age in weeks, and Infection with 7 microbial infections, where blue arrows show positive effects, red blunt-ended lines show negative effects, and line thickness indicates the size of the covariance, which is shown (with the SE in parentheses) for mice from all sites; marginally nonsignificant results are shown by thin dotted lines. All estimates, SE, and *p*-values are shown in S6 Table. In (A), for females root mean square error of approximation (RMSEA) = 0.075 (0.026–0.124), comparative fit index (CFI) = 0.98, standardized root mean square residual (SRMR) = 0.017, χ^2^ = 17.61, df = 8, *p =* 0.024, for males RMSEA = 0.015 (0.0–0.077), CFI = 0.999, SRMR = 0.017, χ^2^ = 8.42, df = 8, *p =* 0.39; (B) for females RMSEA = 0.112 (0.08–0.146), CFI = 0.92, SRMR = 0.047, χ^2^ = 50.98, df = 14, *p <* 0.0001, which is not a significantly good fit, for males RMSEA = 0.067 (0.032–0.10), CFI = 0.972, SRMR = 0.028, χ^2^ = 24.43, df = 14, *p =* 0.009, which is not a significantly good fit; (C) for females RMSEA = 0.103 (0.06–0.149), CFI = 0.83, SRMR = 0.059, χ^2^ = 25.89, df = 8, *p =* 0.0011, with warnings concerning the latent variable immune state, for males RMSEA = 0.052 (0.0–0.099), CFI = 0.93, SRMR = 0.036, χ^2^ = 13.41, df = 8, *p =* 0.098; for immunoglobulin (Ig) A and the latent variable of immune state, the dotted line indicates that this is significant in females but nonsignificant in males, as shown in S6 Table.
(PDF)

S1 Table (A) Mouse sample sites and (B) the intersite distances in km.
(DOCX)

S2 Table Immunological distance shown both within and among sample sites, shown as the mean ± 1 SE. There is no correlation between the within-site immunological distance and the number of mice at that site (*r* = −0.043, *p* = 0.89, *n* = 12).
(DOCX)

S3 Table F_st_ values shown among all sample sites. The local population genetic structure is not driven by geographical distance (S1 Table). Mantel test for correlation between immunological distance and distance and log(distance + 1), respectively: *r* = 0.192, *p* = 0.264, and *r* = 0.082, *p* = 0.339.
(DOCX)

S4 Table A summary of selected class 1–3 structural equation models showing the latent variables and observed variables used. *L* is a latent variable, and *O* is an observed variable. ^1^Previous Infection is the number of microbial infections and so an observed variable. Relevant structural equation modelling (SEM) diagrams are shown in S3 Fig.
(DOCX)

S5 Table A summary of the class 4 structural equation models presented in the sequence in which they were tested, with intervening rows showing the model modifications that preceded each new model. In all models, Age was an observed variable of either eye lens mass (Lens mass) or Age in weeks calculated from eye lens mass as described in the main text; Size was a latent variable consisting of a range of different measures, as shown; Condition was an observed variable of scaled mass index (SMI), the concentration of leptin, or the mass of abdominal fat / body length ratio; Immune State was either a latent variable consisting of the shown immunological parameters, as absolute numbers or percentages of the relevant cells, and concentrations of the different immunoglobulin classes, or a single observed variable as shown. In all models, Season was an observed variable of day length but is not shown in the table. Models were used with data from male and female mice separately. Relevant structural equation modelling (SEM) diagrams are shown in S4 Fig. The goodness of fit to the data for each model was assessed by the root mean square error of approximation (RMSEA), the comparative fit index (CFI), the standardized root mean square residual (SRMR), and the chi-square test of model fit (χ^2^), where RMSEA and SRMR values less than 0.05 were accepted as a good fit of the model to the data; however, RMSEA values greater than 0.05 were accepted where the lower 90% confidence limit was 0.000. CFI values greater than 0.95 were accepted as a very good fit, and anything greater than 0.80 was considered as acceptable. Nonsignificance (*p* > 0.05) for the χ^2^ goodness-of-fit test was used to indicate an acceptable fit of the model to the data. The software also generated warnings concerning component data of the model, and this information was used to refine the model design.
(DOCX)

S6 Table The estimated standardised covariances (Estimate), their standard error (SE), and 2-tailed *p*-values (with *p* < 0.05 shown in bold) for structural equation models of adaptive cellular, innate cellular, and adaptive humoral immune state for female and male mice from all sites, as shown in S6 Fig. Marginally nonsignificant results are marked with *.
(DOCX)

S7 Table The estimated standardised covariances (Estimate), their standard error (SE), and 2-tailed *p*-values (with *p* < 0.05 shown in bold) for structural equation models of adaptive cellular, innate cellular, and adaptive humoral immune state for female and male mice from site HW, as shown in Fig 5. Marginally nonsignificant results are marked with *.
(DOCX)

S1 Text Extended methods for structural equation modelling.
(DOCX)

## Abbreviations

BMI: body mass index
CFI: comparative fit index
DC: dendritic cell
Ig: immunoglobulin
NK: natural killer
PAMP: pathogen-associated molecular pattern
RMSEA: root mean square error of approximation
SEM: structural equation modelling
SMI: scaled mass index
SRMR: standardized root mean square residual
T_reg_: regulatory T cell

## References

[1] Abolins S, Lazarou L, Weldon L, Hughes L, King EC, Drescher P, et al The comparative immunology of wild and laboratory mice *Mus musculus domesticus*. Nat. Comm. 2017;8: 14811. 10.1038/ncomms14811 PMC5418598 28466840

[2] Beura LK, Hamilton SE, Bi K, Schenkel JM, Odumade OA, Casey KA, et al Normalizing the environment recapitulates adult human immune traits in laboratory mice. Nature 2016;532: 512–516. doi: 10.1038/nature17655 27096360 10.1038/nature17655 PMC4871315

[3] Abolins SR, Pocock MJO, Hafalla JCR, Riley EM, Viney ME. Measures of immune function of wild mice, *Mus musculus*. Molec. Ecol. 2011;20: 881–892. 21073587 10.1111/j.1365-294X.2010.04910.x

[4] Lochmiller RL, Vestey MR, McMurray ST. Primary immune responses of selected small mammal species to heterologous erythrocytes. Comp. Biochem. Physiol. A Comp. Physiol. 1991;100: 139–143. 1682088 10.1016/0300-9629(91)90196-j

[5] Boysen P, Eide DM, Storset AK. Natural killer cells in free-living *Mus musculus* have a primed phenotype. Mol. Ecol. 2011;20: 5103–5110. doi: 10.1111/j.1365-294X.2011.05269.x 21895821 10.1111/j.1365-294X.2011.05269.x

[6] Devalapalli AP, Lesher A, Shieh K, Solow JS, Everett ML, Edala AS, et al. Increased levels of IgE and autoreactive, polyreactive IgG in wild rodents: implications for the hygiene hypothesis. Scand. J. Immunol. 2006;64: 125–136. doi: 10.1111/j.1365-3083.2006.01785.x 16867157 10.1111/j.1365-3083.2006.01785.x

[7] Viney ME, Riley ME. The immunology of wild rodents: current status and future prospects. Front. Immunol. 2017;8: 1481 doi: 10.3389/fimmu.2017.01481 29184549 10.3389/fimmu.2017.01481 PMC5694458

[8] Han BA, Kramer AM, Drake JM. Global patterns of zoonotic disease in mammals. Trend Parasitol. 2016;32: 565–577 10.1016/j.pt.2016.04.007 PMC4921293 27316904

[9] Plowright RK, Parrish CR, McCallum H, Hudson PJ, Ko AI, Graham AL, et al Pathways to zoonotic spillover. Nat Rev Microbiol. 2017;15: 502–510. doi: 10.1038/nrmicro.2017.45 28555073 10.1038/nrmicro.2017.45 PMC5791534

[10] Schmid-Hempel P. Variation in immune defence as a question of evolutionary ecology. Proc. Roy. Soc. B 2003;270: 357–366. 10.1098/rspb.2002.2265 PMC1691258 12639314

[11] Martin LB, Weil ZM, Nelson RJ. Seasonal changes in vertebrate immune activity: mediation by physiological trade-offs. Phil. Trans. R. Soc. B 2008;363: 321–339. doi: 10.1098/rstb.2007.2142 17638690 10.1098/rstb.2007.2142 PMC2606753

[12] Nelson RT, Demas GE. Seasonal changes in immune function. Q. Rev. Biol. 1996;71: 511–548. 8987173 10.1086/419555

[13] Nelson RJ, Demas GE, Klein SA. (2002). Seasonal patterns of stress, immune function and disease CUP.

[14] Altizer S, Dobson A, Hosseini P, Hudson P, Pascual M, Rohani P. Seasonality and the dynamics of infectious diseases. Ecol. Lett. 2009;9: 467–484. 10.1111/j.1461-0248.2005.00879.x 16623732

[15] Viney ME, Riley EM. (2014). From immunology to eco-immunology: more than a new name, pp 1–19 In: *Eco-immunology*: *evolutive aspects and future perspectives* (eds Malagoli D. and Ottaviani E.). Springer, UK.

[16] Beldomenico PM, Telfer S, Gebert S, Lukomski L, Bennett M, Begon M. Poor condition and infection: a vicious circle in natural populations. Proc. Roy. Soc. B 2008;275: 1753–1759. 10.1098/rspb.2008.0147 PMC2453294 18448414

[17] Maue AC, Yager EJ, Swain SL, Woodland DL, Blackman MA, Haynes L. T-cell immunosenescence: lessons learned from mouse models of aging. Trends Immunol. 2009;30: 301–305. doi: 10.1016/j.it.2009.04.007 19541537 10.1016/j.it.2009.04.007 PMC3755270

[18] Lynch HE, Goldberg GL, Chidgey A, van den Brink MR, Boyd R, Sempowski GD. Thymic involution and immune reconstitution. Trends Immunol. 2009;30: 366–373. doi: 10.1016/j.it.2009.04.003 19540807 10.1016/j.it.2009.04.003 PMC2750859

[19] Martin LB, Weil ZM, Nelson RJ. Refining approaches and diversifying directions in ecoimmunology. Integr. Comp. Biol. 2006;46: 1030–1039. doi: 10.1093/icb/icl039 21672805 10.1093/icb/icl039

[20] Nussey DH, Watt K, Pilkington JG, Zamoyska R, McNeilly TN. Age-related variation in immunity in a wild mammal population. Aging Cell 2012;11: 178–180. doi: 10.1111/j.1474-9726.2011.00771.x 22107028 10.1111/j.1474-9726.2011.00771.x PMC3397677

[21] Watson RL, McNeilly TN, Watt KA, Pemberton JM, Pilkington JG, Waterfall M, et al Cellular and humoral immunity in a wild mammal: variation with age and sex and association with overwinter survival. Ecol. Evol. 2016;6: 8695–8705. doi: 10.1002/ece3.2584 28035261 10.1002/ece3.2584 PMC5192870

[22] Yang H, Wang JR, Didion JP, Buus RJ, Bell TA Welsh CE, et al Subspecific origin and haplotype diversity in the laboratory mouse. Nature 2011;43: 648–655. 10.1038/ng.847 PMC3125408 21623374

[23] Laurie CC, Nickerson DA, Anderson AD, Weir BS, Livingston RJ, Dean MD, et al Linkage disequilibrium in wild mice. PLoS Genet. 2007;3: e144 doi: 10.1371/journal.pgen.0030144 17722986 10.1371/journal.pgen.0030144 PMC1950958

[24] Latham N, Mason G. From house mouse to mouse house: the behavioural biology of free-living *Mus musculus* and its implications in the laboratory. Appl. Anim. Behav. Sci. 2004;86: 261–289.

[25] Pocock MJO, Hauffe HC, Searle JB. Dispersal in house mice. Biol. J. Linnean Soc. 2005;84: 565–583.

[26] Becker SD, Bennett M, Stewart JP, Hurst JL. Serological survey of virus infection among wild house mice (*Mus domesticus*) in the UK. Lab. Anim. 2007;41: 229–238. doi: 10.1258/002367707780378203 17430622 10.1258/002367707780378203

[27] Singelton GR, Smith AL, Krebs CJ. The prevalence of viral antibodies during a large population fluctuation of house mice in Australia. Epidemiol. Infect. 2000;125: 719–727. 11218223 10.1017/s0950268800004945 PMC2869656

[28] Carr EJ, Dooley J, Garcia-Perez JE, Lagou V, Lee C, Wouters C, et al The cellular composition of the human immune system is shaped by age and cohabitation. Nat. Immunol. 2016;17: 461–468. doi: 10.1038/ni.3371 26878114 10.1038/ni.3371 PMC4890679

[29] Arriero E, Wanelik KM, Birtles RJ, Bradley JE, Jackson JA, Paterson S, et al From the animal house to the field: Are there consistent individual differences in immunological profiles in wild populations of field voles (*Microtus agrestis)*? PLoS ONE. 2017;12: e0183450 doi: 10.1371/journal.pone.0183450 28817724 10.1371/journal.pone.0183450 PMC5560671

[30] Andrianaivoarimanana V, Telfer S, Rajerison M, Ranjalahy MA, Andriamiarimanana F, Rahaingosoamamitiana C, et al Immune responses to plague infection in wild *Rattus rattus*, in Madagascar: a role in foci persistence? PLoS ONE. 2012;7: e38630 doi: 10.1371/journal.pone.0038630 22719908 10.1371/journal.pone.0038630 PMC3377696

[31] Schuurs AHWM Verheul HAM. Effects of gender and sex steroids on the immune response. J. Steroid Biochem. 1990;35: 157–172. 2407902 10.1016/0022-4731(90)90270-3

[32] Nussey DH, Watt KA, Clark A, Pilkington JG, Pemerton JM, Graham AL, et al Multivariate immune defences and fitness in the wild: complex but ecologically important associations among plasma antibodies, health and survival. Proc. Roy. Soc. B 2014;281: 20132931. 10.1098/rspb.2013.2931 PMC3924079 24500168

[33] Krebs CJ, Singleton GR. Indexes of condition for small mammals. Aust. J. Zool. 1993;41: 317–323.

[34] Rowe FP, Bradfield A, Quy RJ, Swinney T. Relationship between eye lens weight and age in the wild mouse (*Mus musculus*). J. App. Ecol. 1985;22: 55–61.

[35] Peig J, Green AJ. New perspectives for estimating body condition from mass/length data: the scaled mass index as an alternative method. Oikos 2009;118: 1883–1891.

[36] Maffei M, Halaas J, Ravussin E, Pratley RE, Lee GH, Zhang Y, et al Leptin levels in human and rodent: Measurement of plasma leptin and *ob* RNA in obese and weight-reduced subjects. Nat. Med. 1995;1: 1155–1161. 7584987 10.1038/nm1195-1155

[37] Idaghdour Y, Czika W, Shianna KV, Lee SH, Visscher PM, Martin HM, et al Geographical genomics of human leukocyte gene expression variation in southern Morocco Nat. Genet. 2010;42: 62–67. 10.1038/ng.495 PMC2798927 19966804

[38] Wilder SM, Raubenheimer D, Simpson SJ. Moving beyond body condition indices as an estimate of fitness in ecological and evolutionary studies. Funct. Ecol. 2016;30: 108–115.

[39] Labocha MK, Schutz H, Hayes JP. Which body condition index is best? Oikos 2014;123: 111–119.

[40] Viney ME, Riley EM, Buchanan KL. Optimal immune responses: immunocompetence revisited. Trend. Ecol. Evol. 2015;20: 665–669. 10.1016/j.tree.2005.10.003 16701455

[41] Weldon LW, Abolins S, Lenzi L, Bourne C, Riley EM, Viney ME. The gut microbiota of wild mice. PLoS ONE. 2015;10: e0134643 doi: 10.1371/journal.pone.0134643 26258484 10.1371/journal.pone.0134643 PMC4530874

[42] Foll M, Gaggiotti O. A genome-scan method to identify selected loci appropriate for both dominant and codominant markers: a Bayesian perspective. Genetics 2008;180: 977–993. doi: 10.1534/genetics.108.092221 18780740 10.1534/genetics.108.092221 PMC2567396

[43] Excoffier L, Lischer HEL. Arlequin suite ver 3.5: a new series of programs to perform population genetics analyses under Linux and Windows. Molec. Ecol. Res. 2010;10: 564–567. 10.1111/j.1755-0998.2010.02847.x 21565059

[44] Pritchard JK, Stephens M, Donnelly P. Inference of population structure using multilocus genotype data. Genetics 2000;155: 945–959. 10835412 10.1093/genetics/155.2.945 PMC1461096

[45] Earl DA, vonHoldt BM. STRUCTURE HARVESTER: a website and program for visualizing STRUCTURE output and implementing the Evanno method. Conserv. Genet. Resour. 2012;4: 359–361.

[46] Kopelman NM, Mayzel J, Jakobson M, Rosenberg NA, Mayrose I. Clumpak: a program for identifying clustering modes and packaging population structure inferences across *K*. Molec. Ecol. 2015;15: 1179–1191. 10.1111/1755-0998.12387 PMC4534335 25684545

[47] Scornavacca C, Linz S, Albrecht B. A first step toward computing all hybridization networks for two rooted binary phylogenetic trees. Bioinformatics 2011;27: i248–i256. doi: 10.1093/bioinformatics/btr210 23134319 10.1089/cmb.2012.0192

[48] Tamura K, Stecher G, Peterson D, Filipski A, Kumar S. MEGA6: Molecular Evolutionary Genetics Analysis Version 6.0. Mol. Biol. Evol. 2013;30: 2725–2729. doi: 10.1093/molbev/mst197 24132122 10.1093/molbev/mst197 PMC3840312

[49] Grace JB. (2006). Structural Equation Modelling and Natural Systems CUP.

[50] Stride C. (2010). Structural Equation Modelling using Mplus, www.figureitout.org.uk

[51] Muthén LK, Muthén B.O. (1998–2011). Mplus User’s Guide. Sixth Edition Los Angeles, CA Muthén & Muthén.

