# Supplementary figures and images for: The ecology of immune state in a wild mammal, Mus musculus domesticus
Source: PLoS Biol. 2018 Apr 13;16(4):e2003538. doi: 10.1371/journal.pbio.2003538 (PMC5919074; doi:10.1371/journal.pbio.2003538)

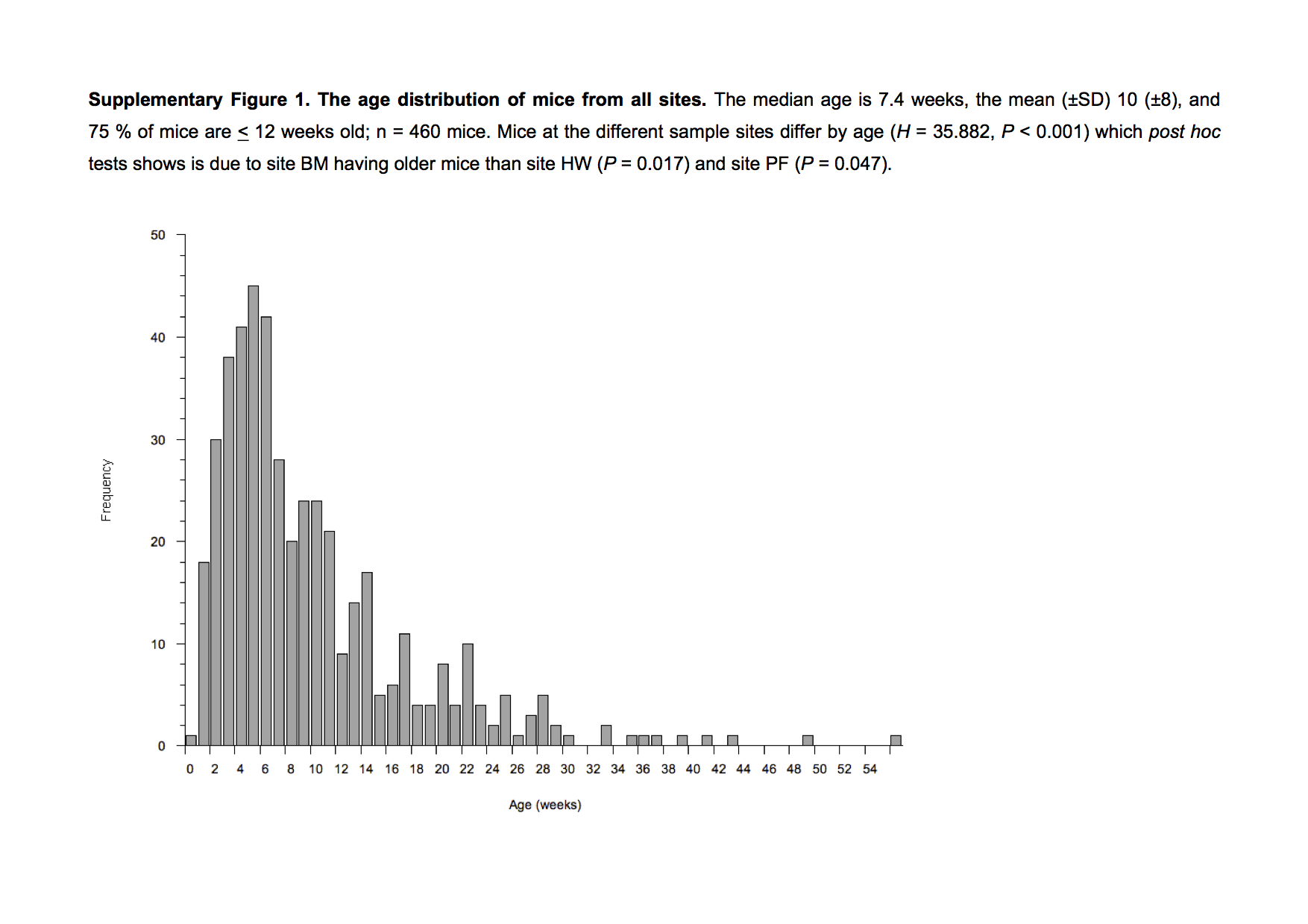

Supplement: S1 Fig — The age distribution of mice from all sites. The median age is 7.4 weeks, the mean (±SD) is 10 (±8), and 75% of mice are ≤ 12 weeks old; n = 460 mice. Mice at the different sample sites differ by age (H = 35.882, p < 0.001), which post hoc tests show is due to site BM having older mice than sites HW (p = 0.017) and PF (p = 0.047). (TIFF) [file pbio.2003538.s007.tiff]

Supplementary Figure 2A

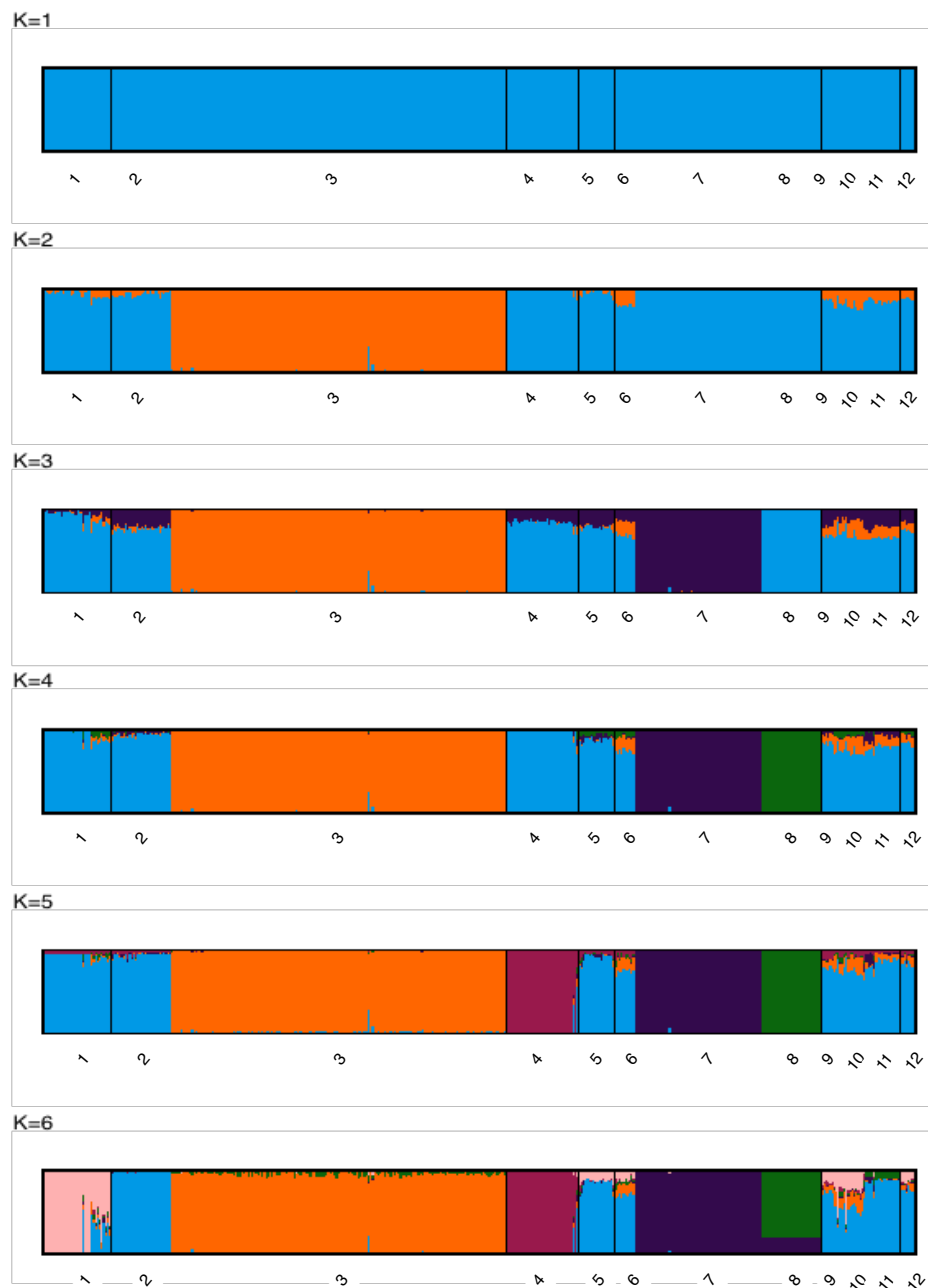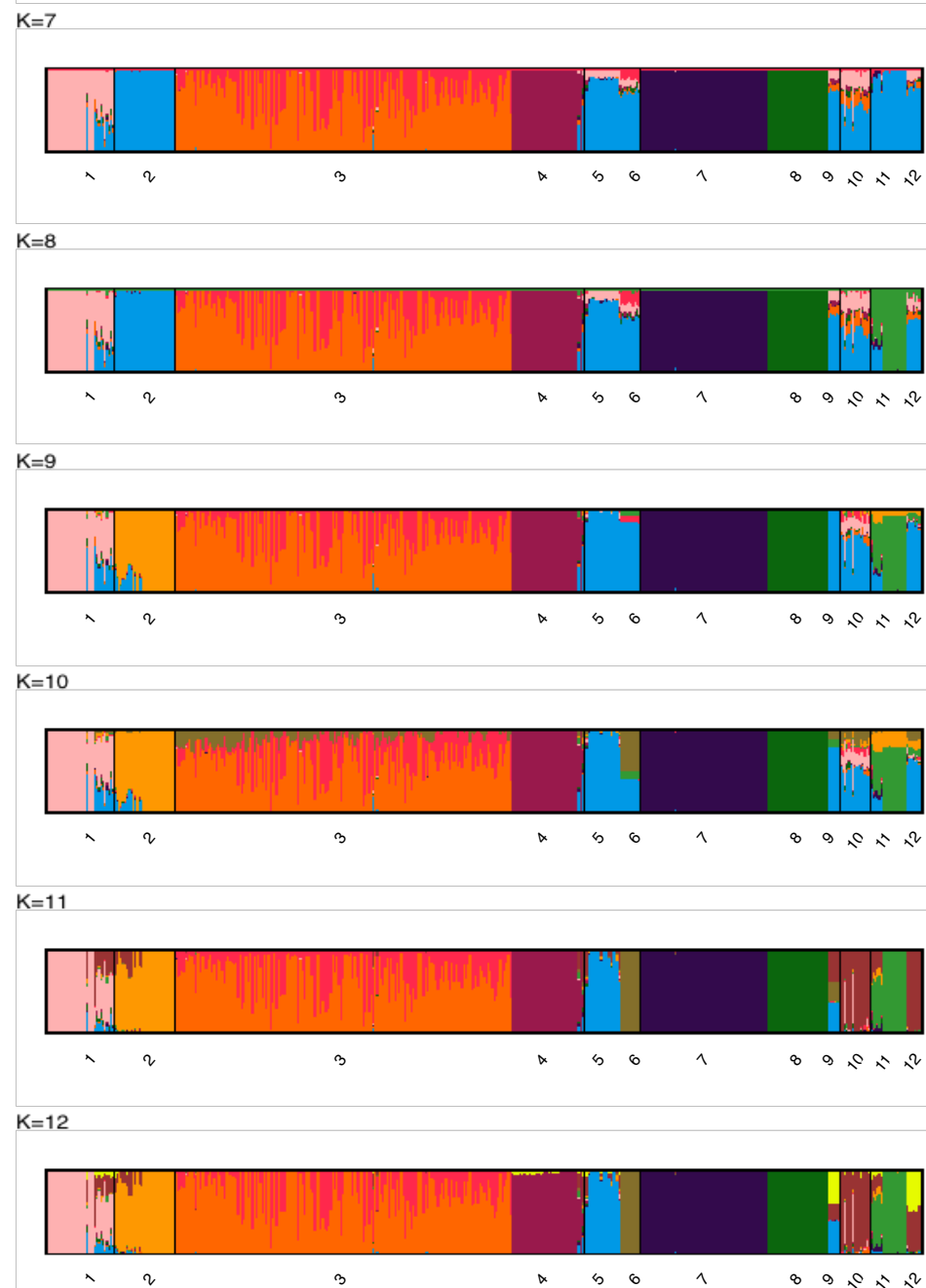

Supplementary Figure 2B

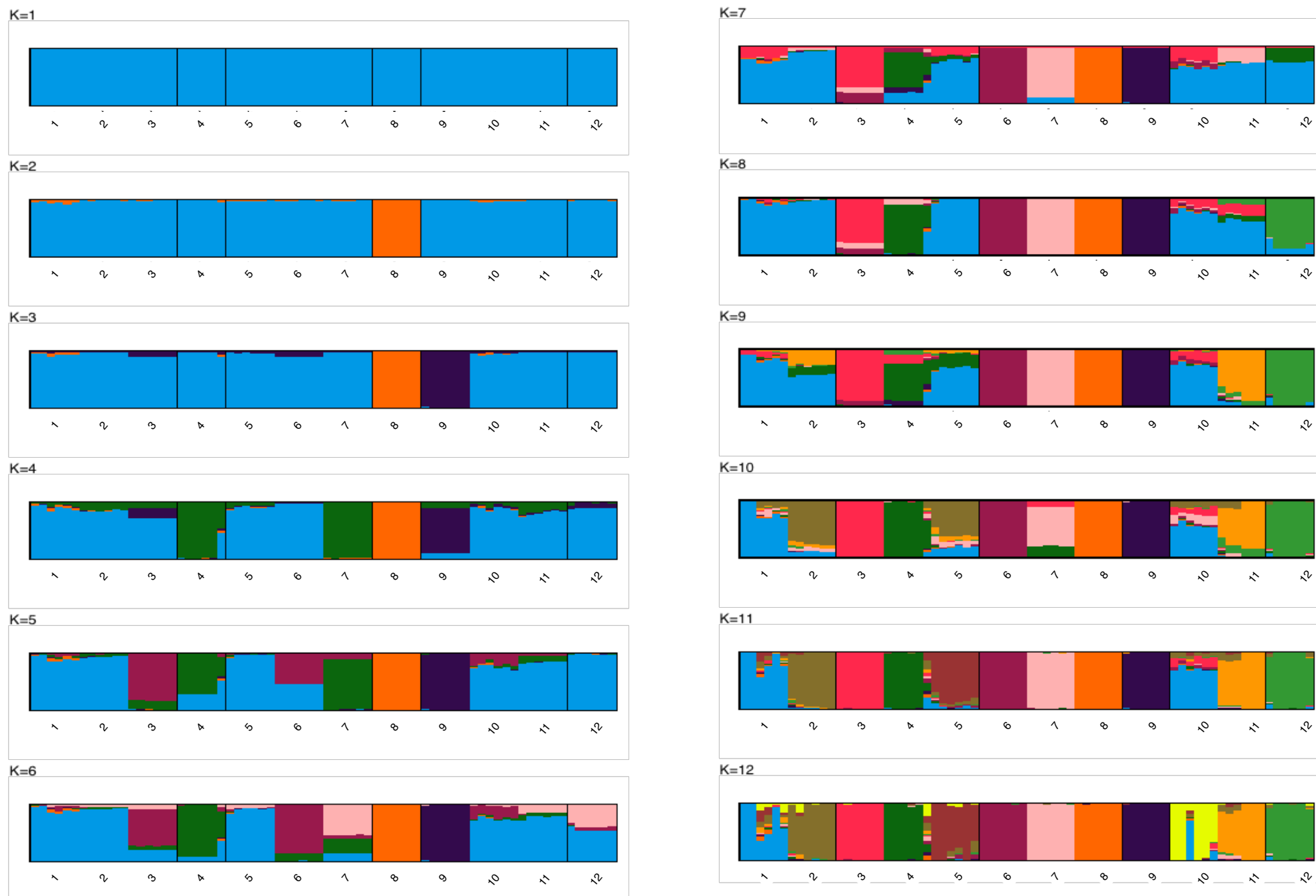

Supplement: S2 Fig — STRUCTURE analysis of mice for 1–12 clusters, K, (A) for all mice and (B) for a random selection of 6 mice from each sample site. In both, there were 15 iterations, and representative figures are shown. The sample site codes for the mice are: 1 = BM, 2 = GL, 3 = HW, 4 = JB, 5 = LU, 6 = PF, 7 = PH, 8 = SK, 9 = SP, 10 = ST, 11 = WF, 12 = WT. The colour order is the same in (A) and (B), where K1 = light blue, K2 = orange, K3 = purple, K4 = green, K5 = maroon, K6 = light pink, K7 = fuchsia, K8 = light green, K9 = dark yellow, K10 = khaki, K11 = brown, and K12 = light yellow. In (A), STRUCTURE harvester shows that K = 9 is the most likely value of K, and cluster resolution largely followed the order of decreasing sample size. In (B), the order in which clusters resolved was closer to the cluster FST values. (PDF) [file pbio.2003538.s008.pdf]

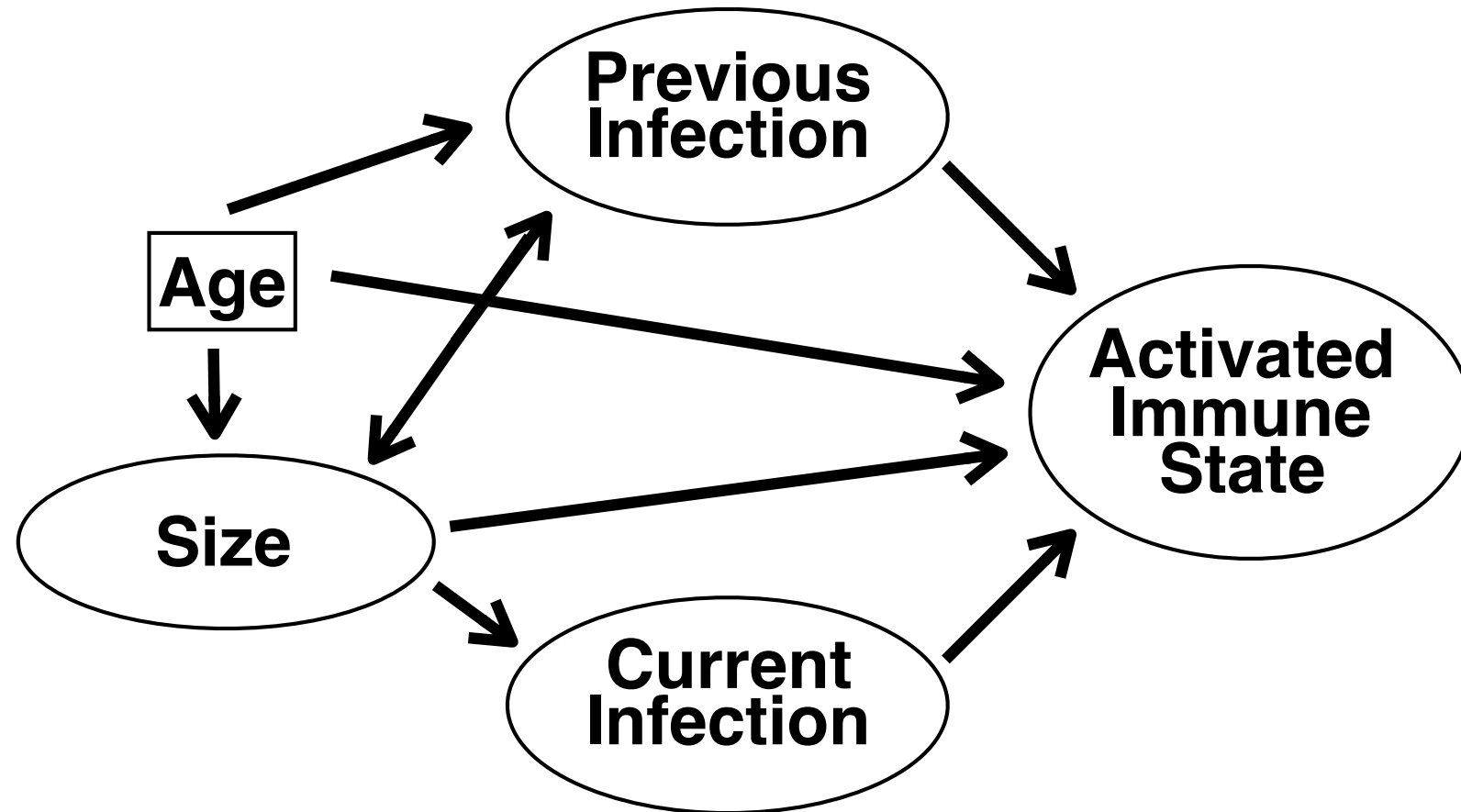

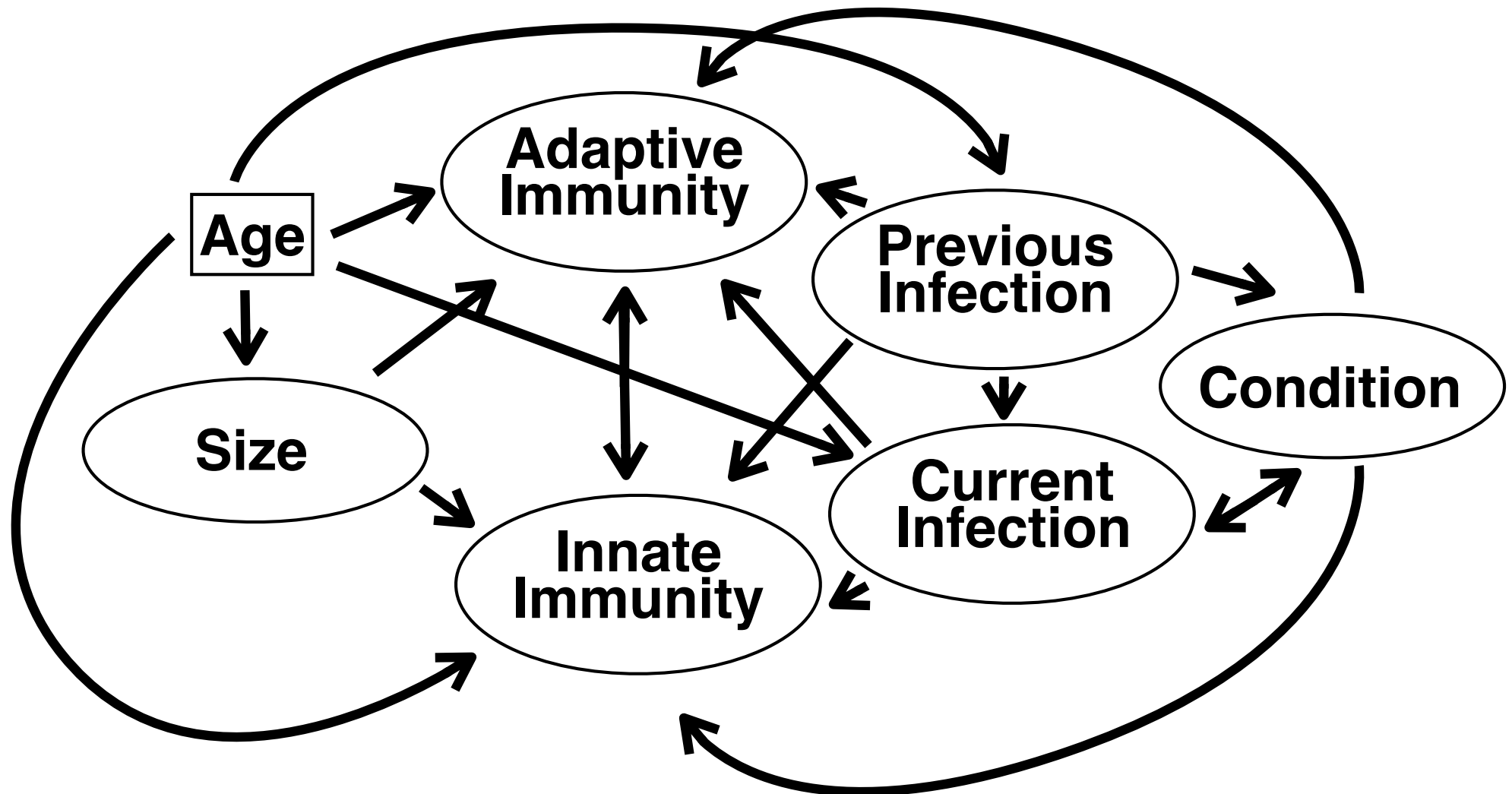

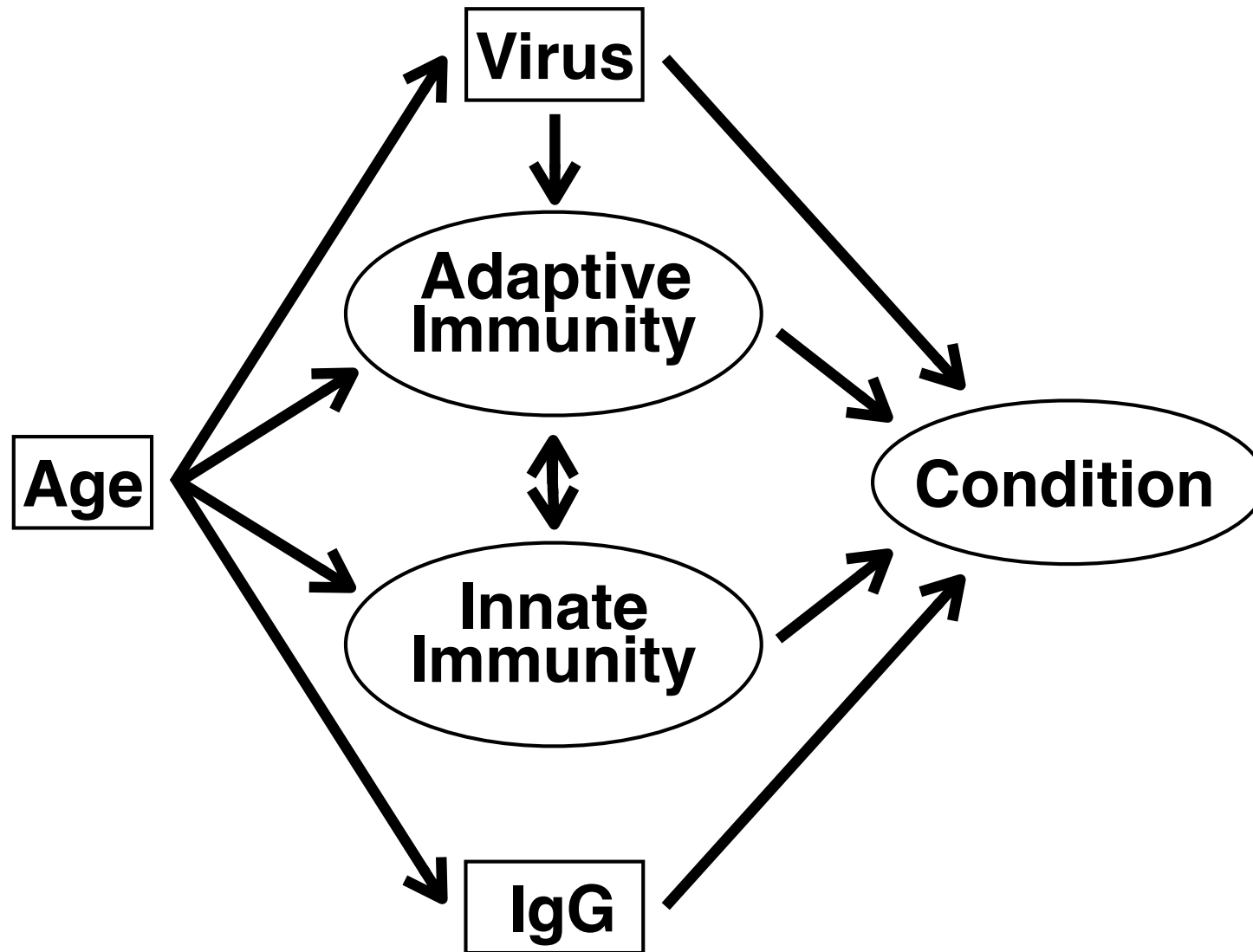

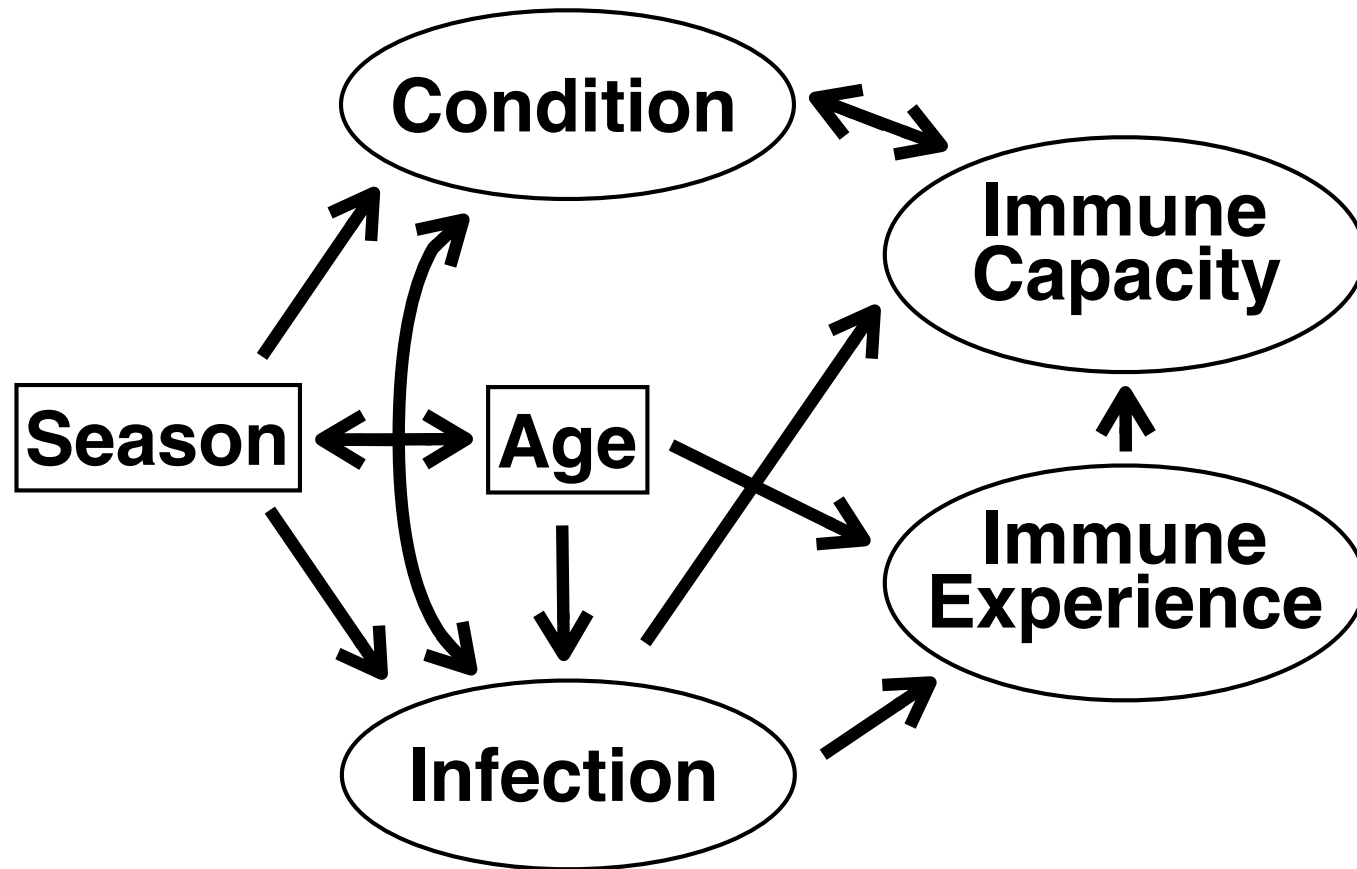

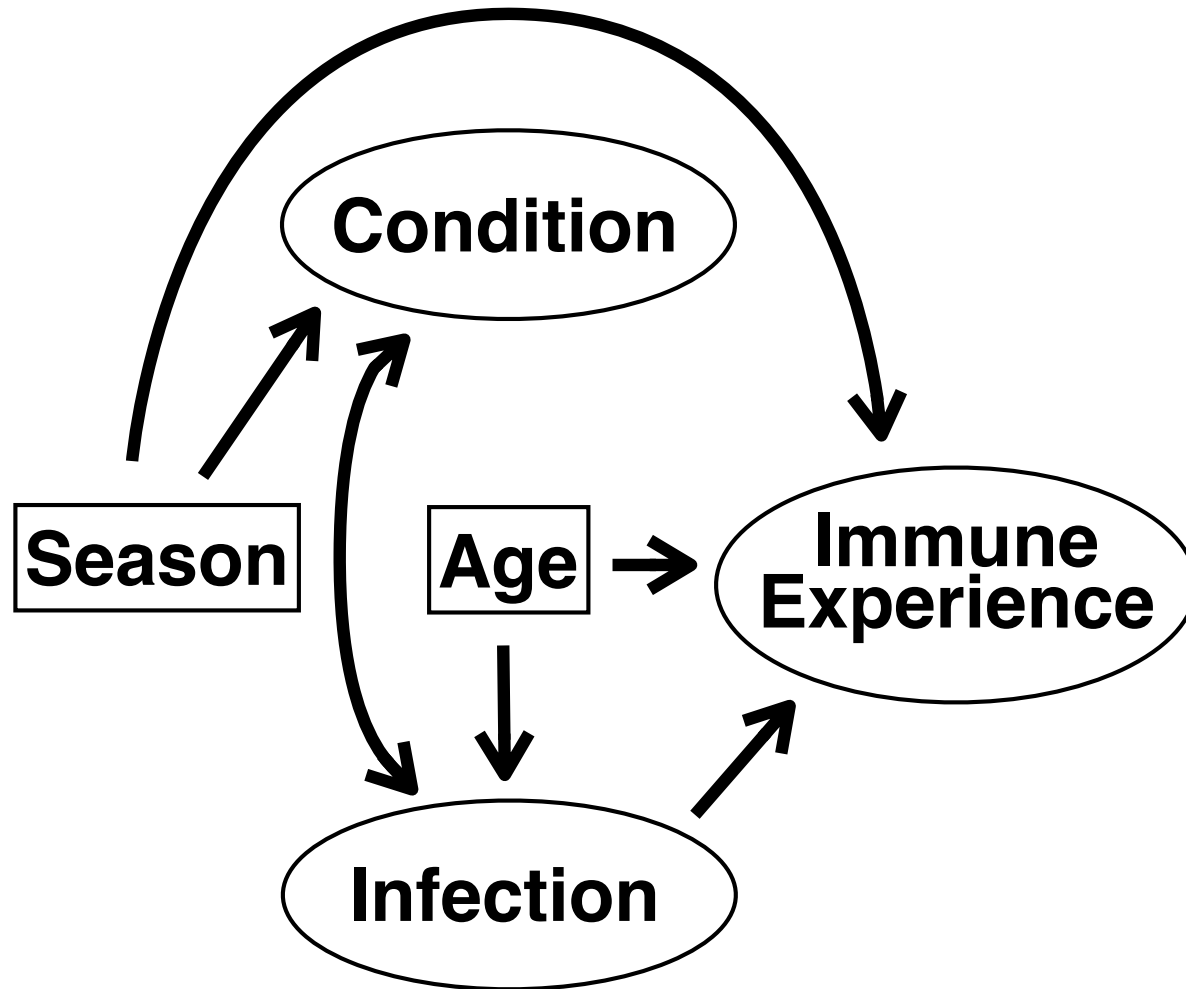

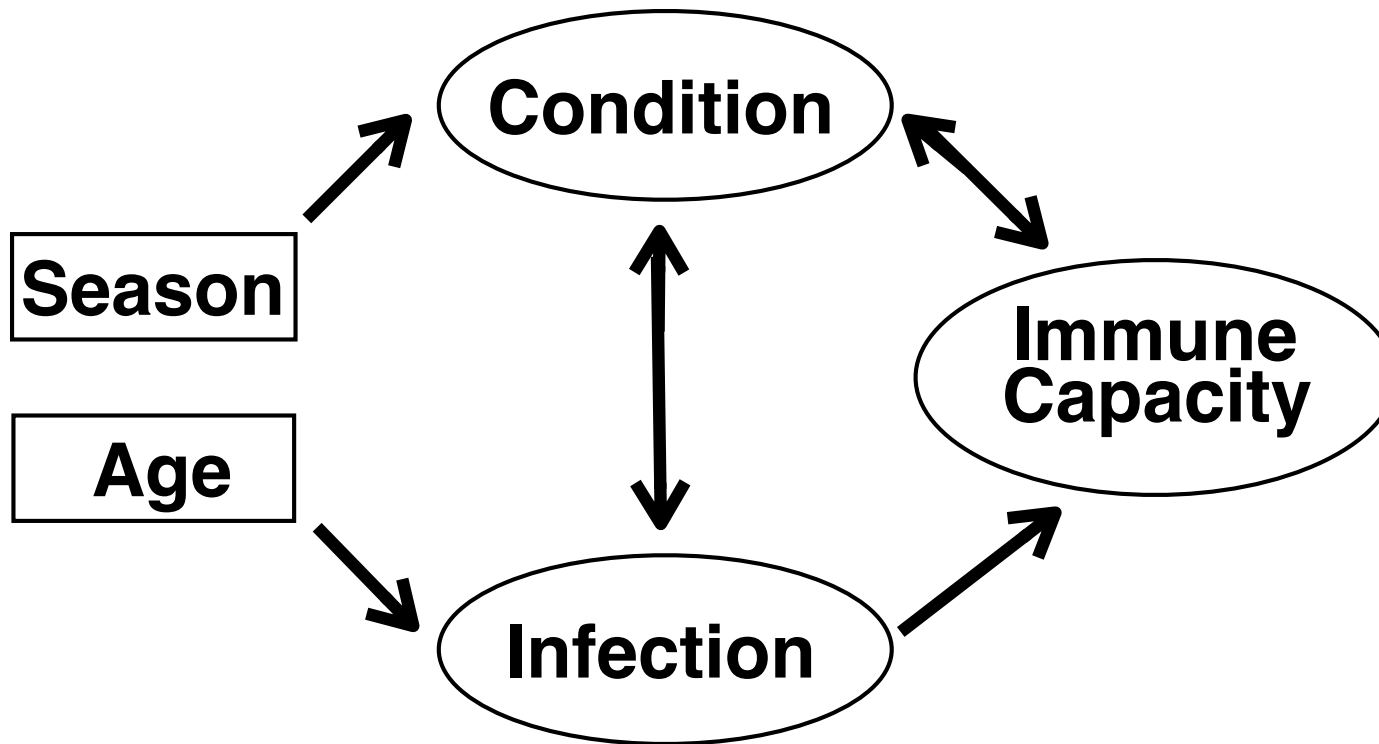

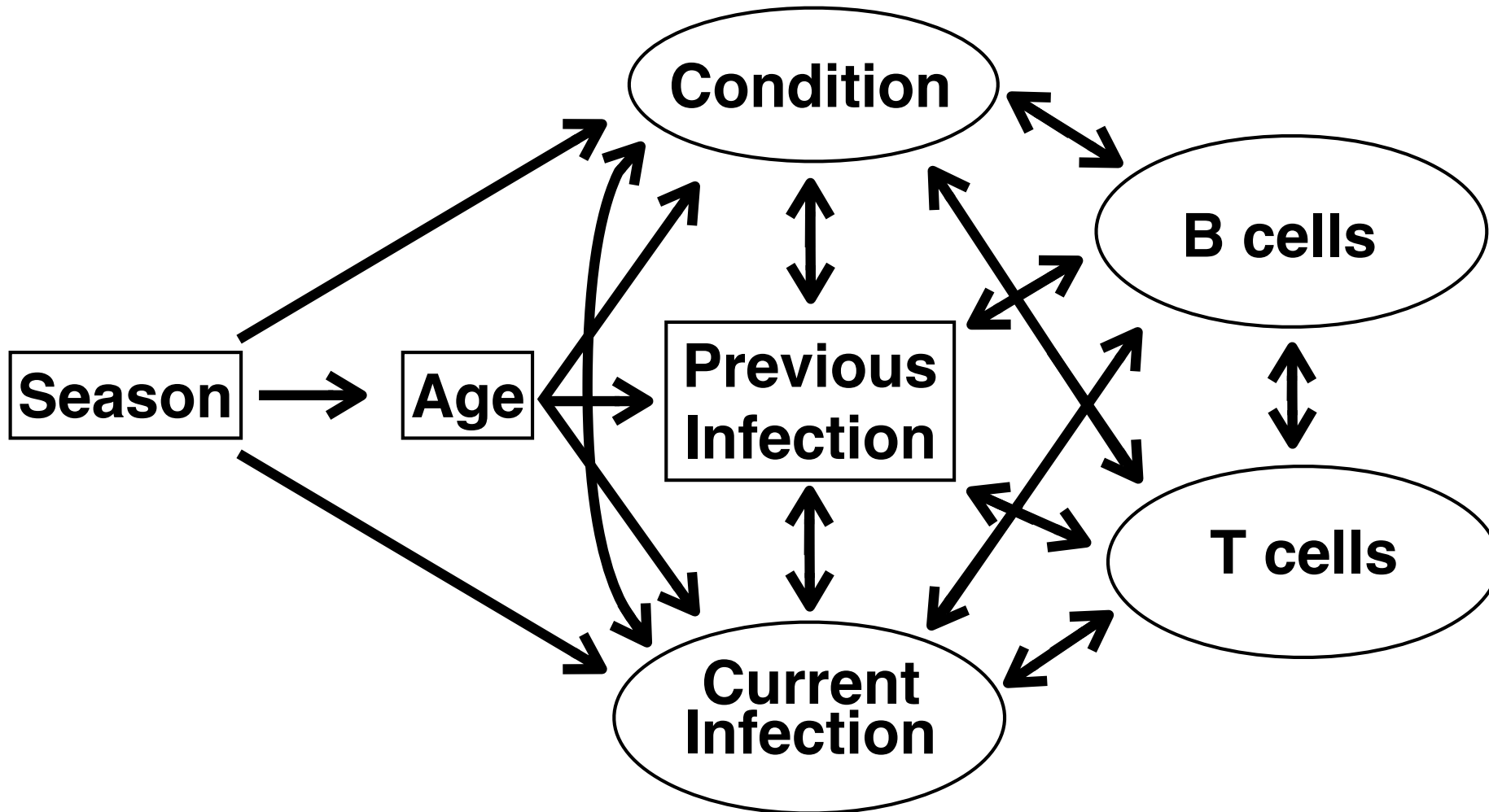

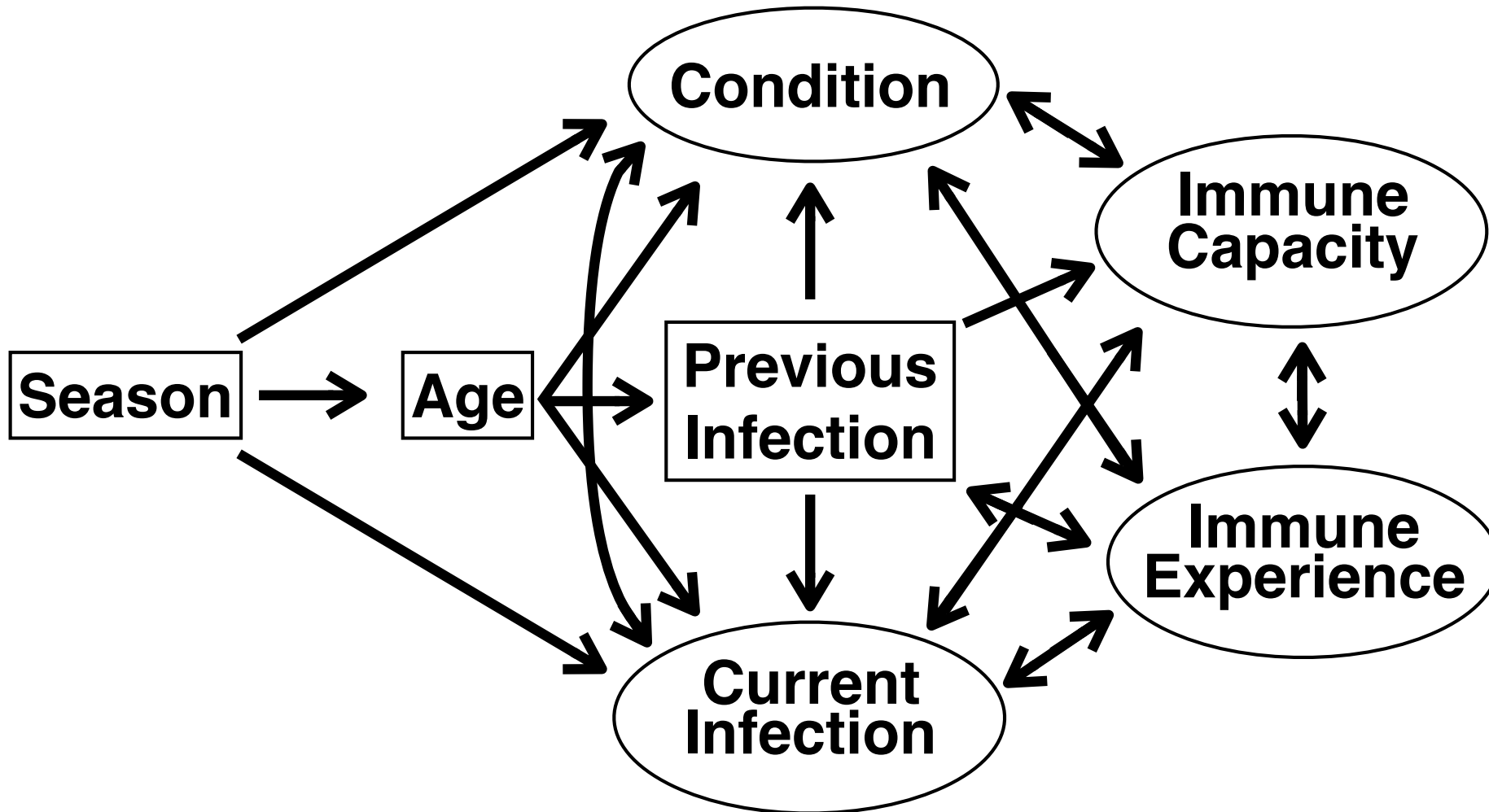

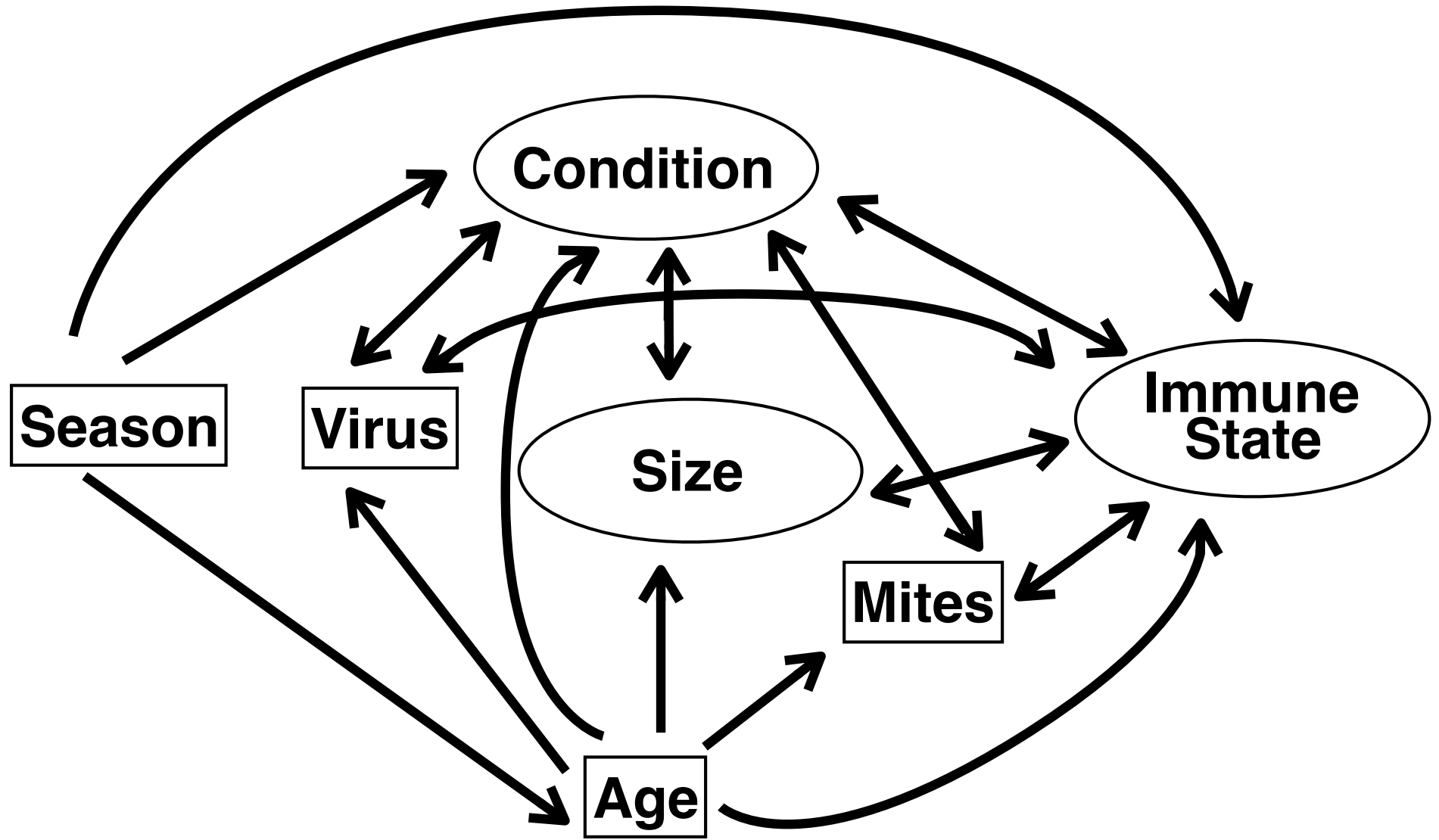

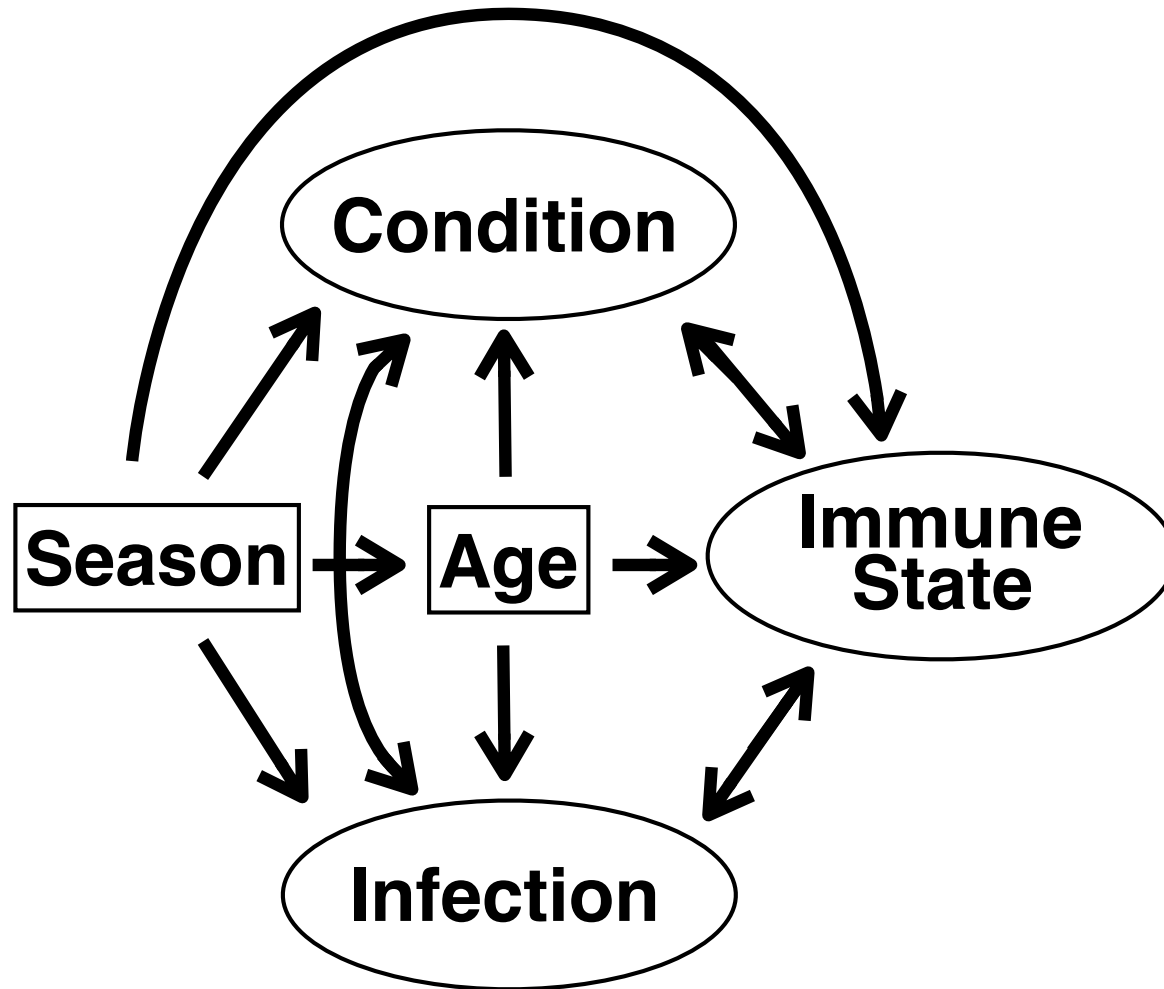

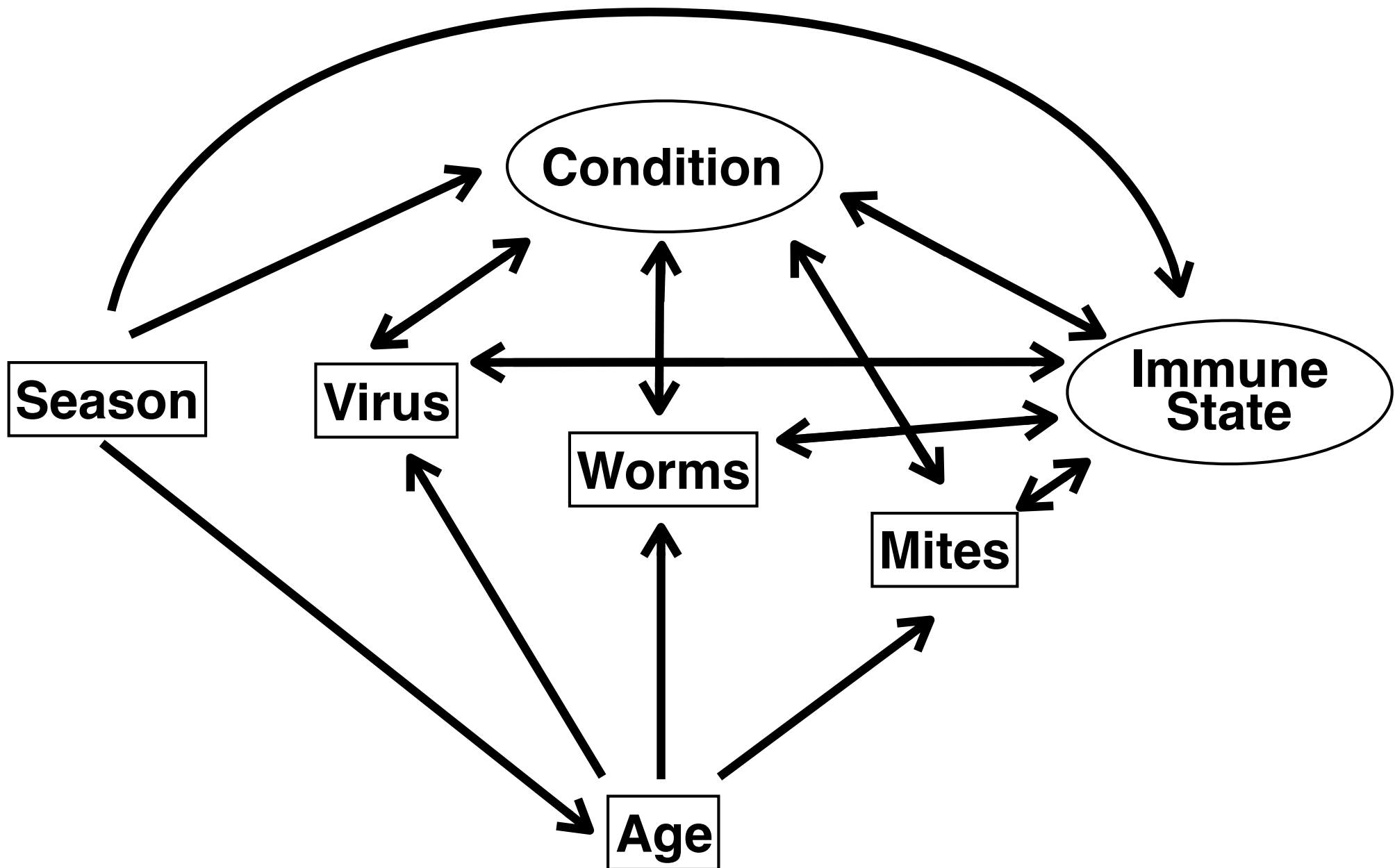

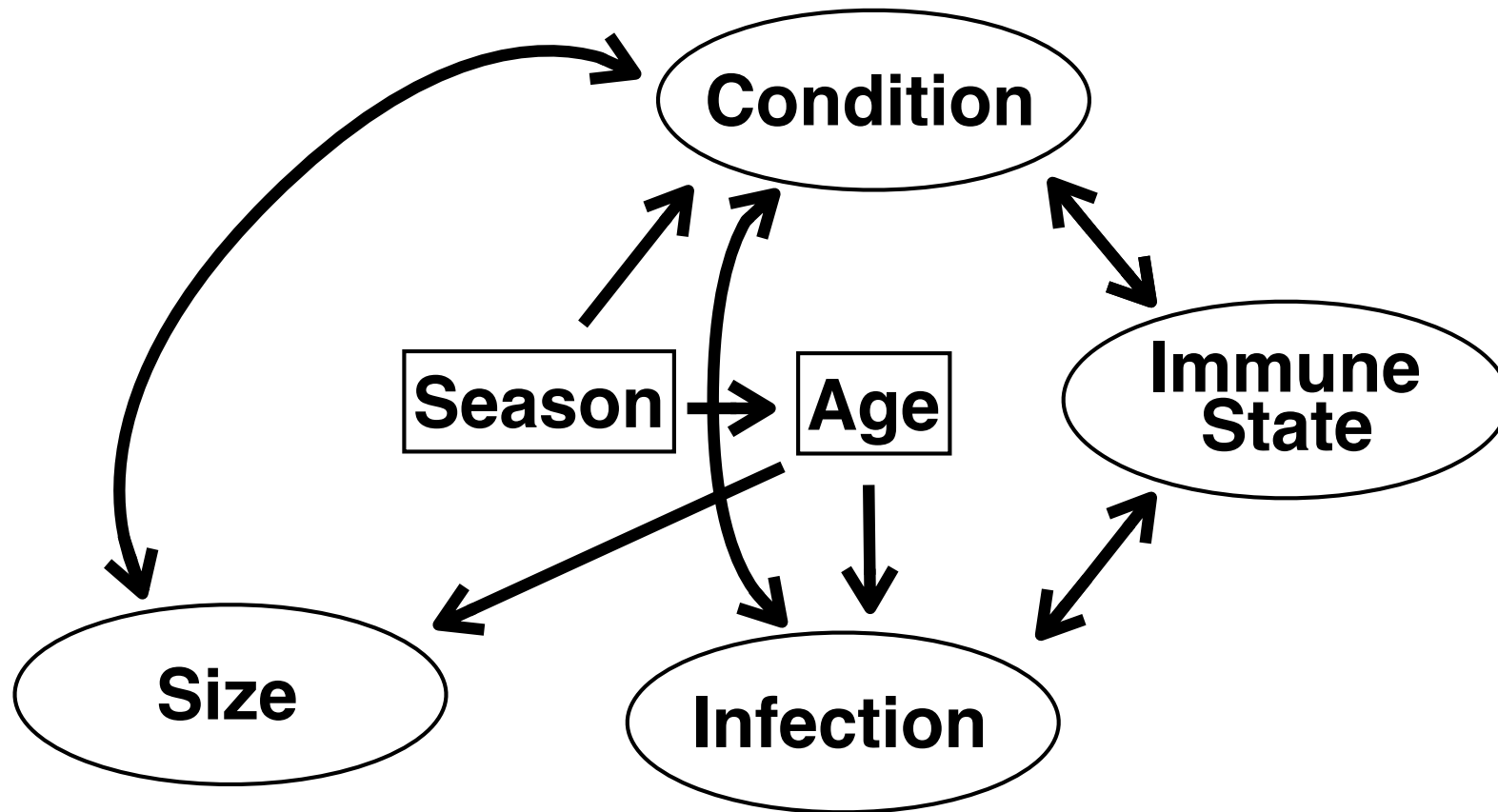

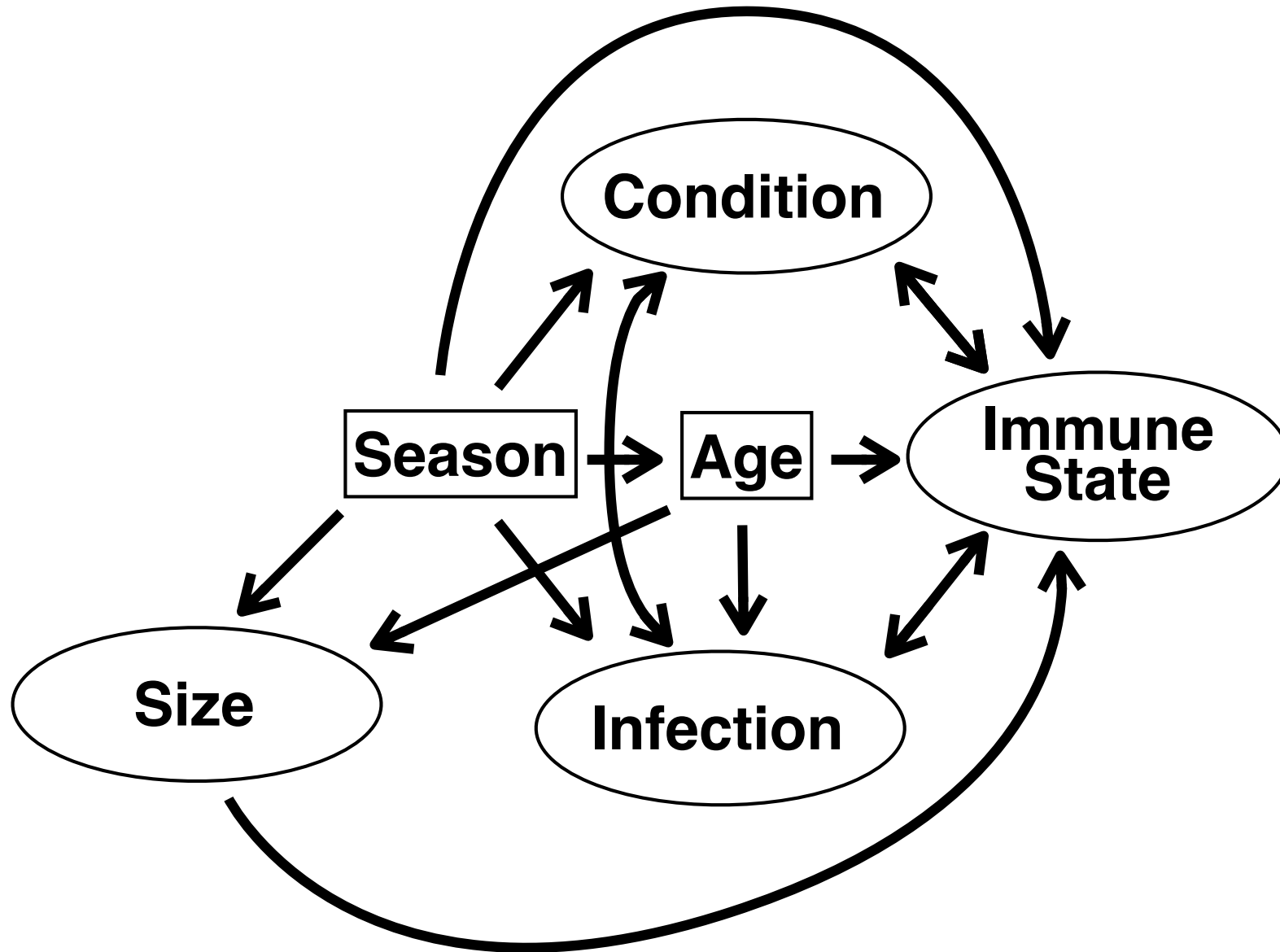

Supplement: S3 Fig — Class 1–3 structural equation models, where latent variables are shown as circles and observed variables are shown as boxes. Individual numbered models refer to S4 Table. (PDF) [file pbio.2003538.s009.pdf]

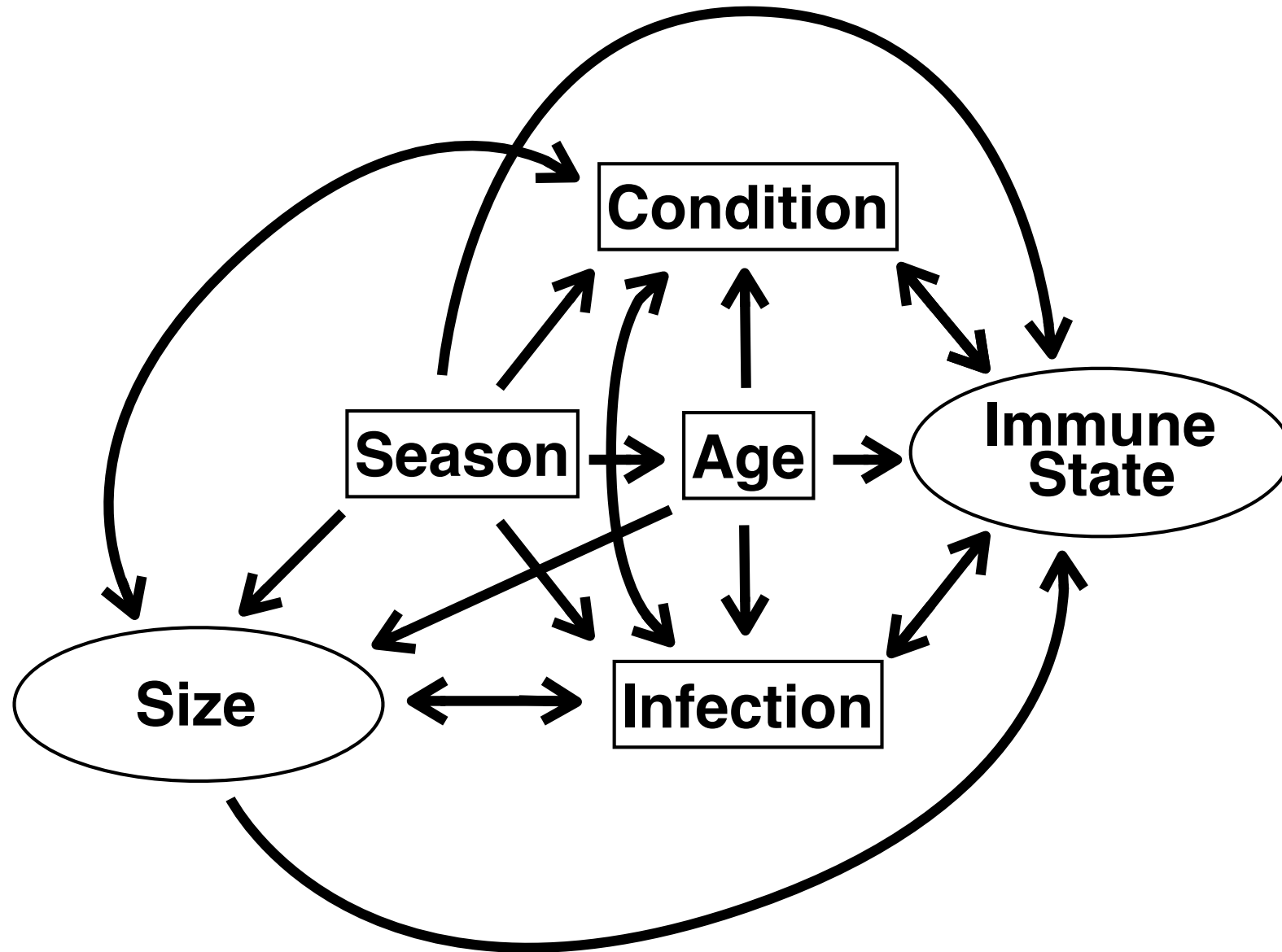

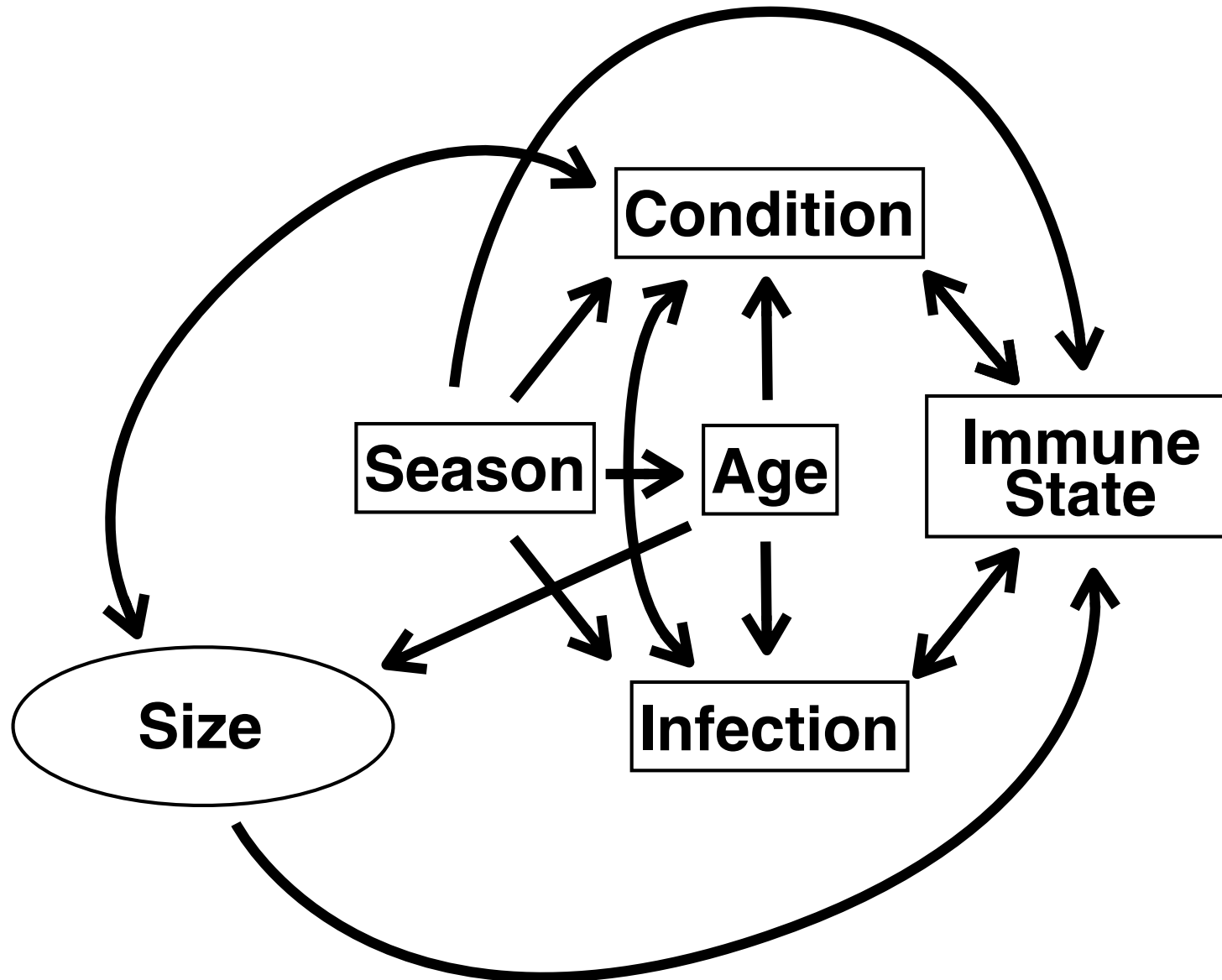

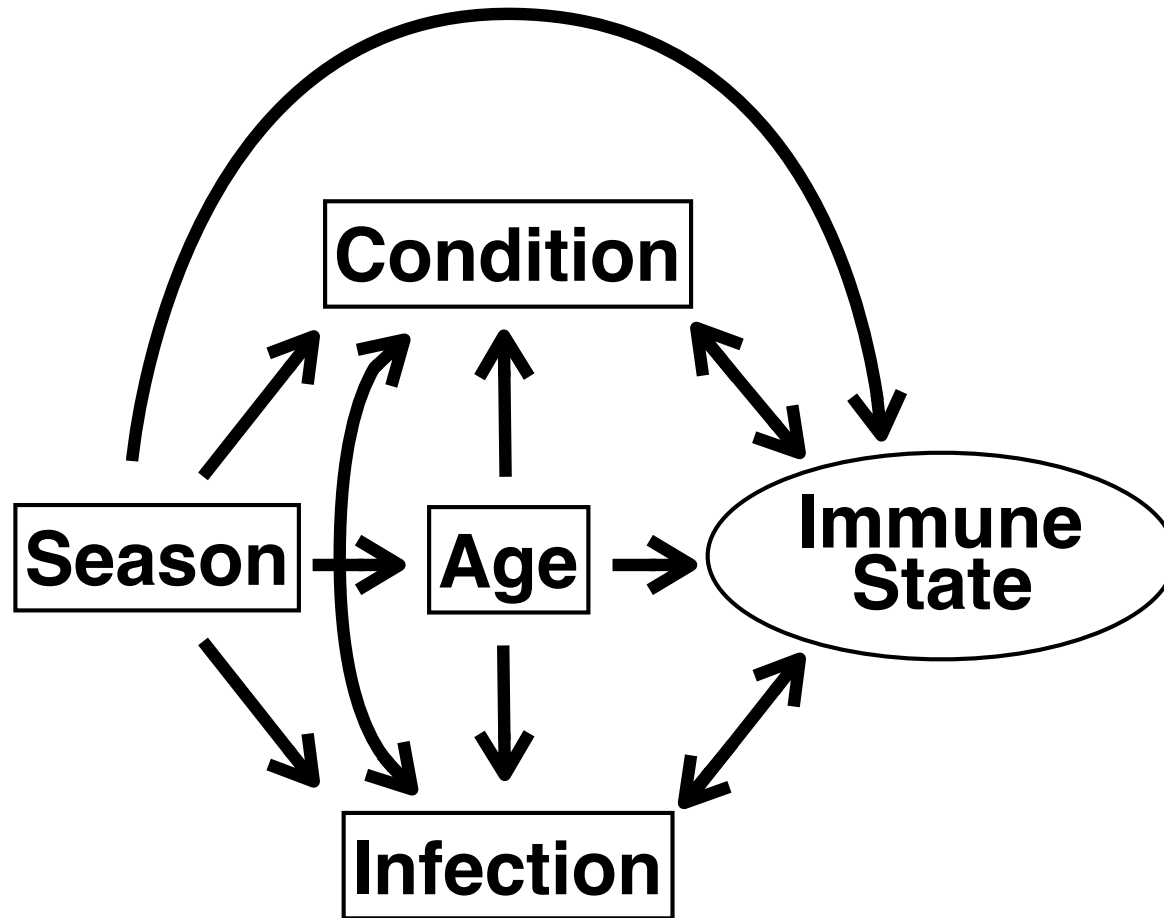

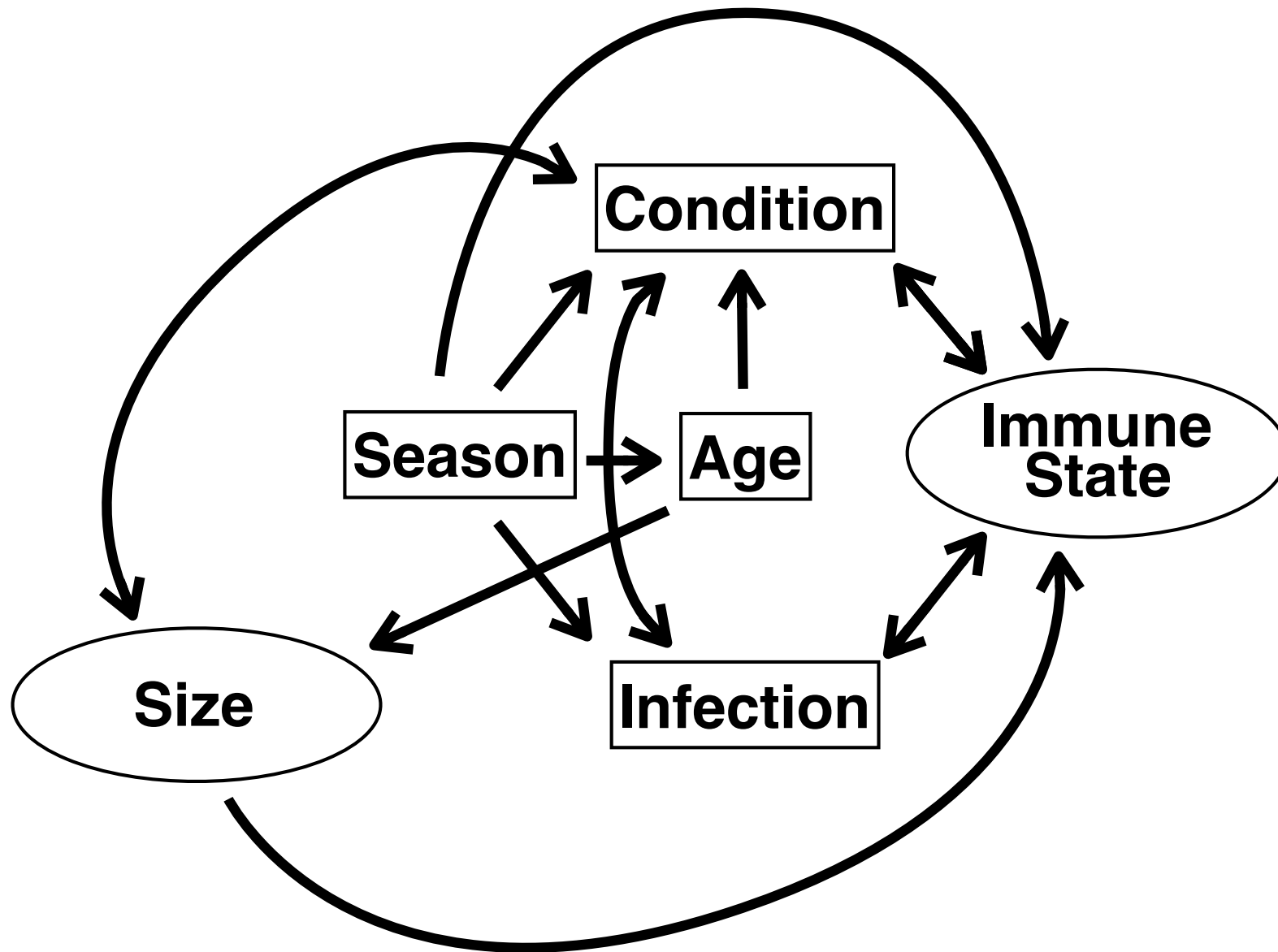

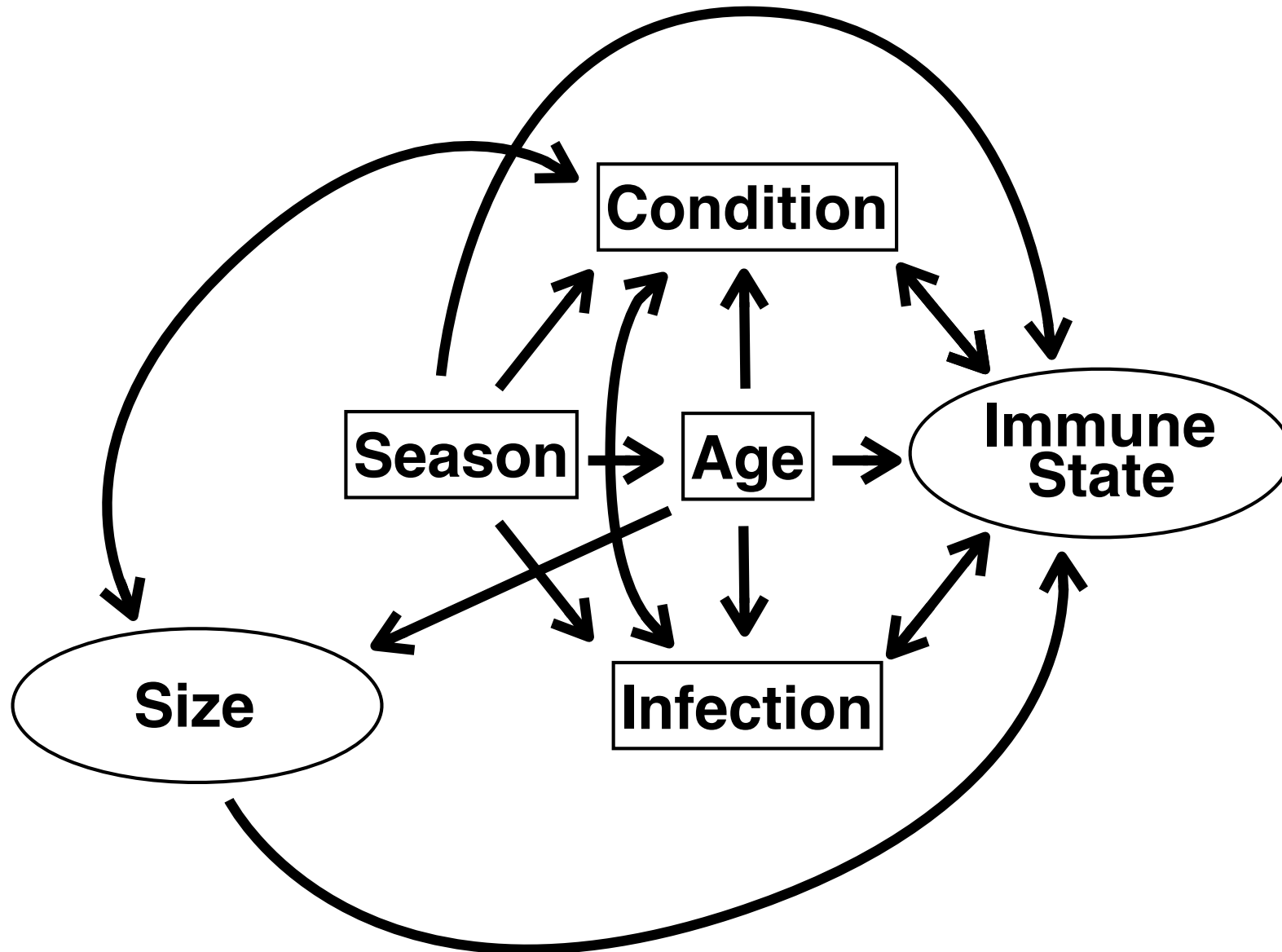

Supplement: S4 Fig — Class 4 structural equation models, where latent variables are shown as circles and observed variables are shown as boxes. Individual numbered models refer to S5 Table. (PDF) [file pbio.2003538.s010.pdf]

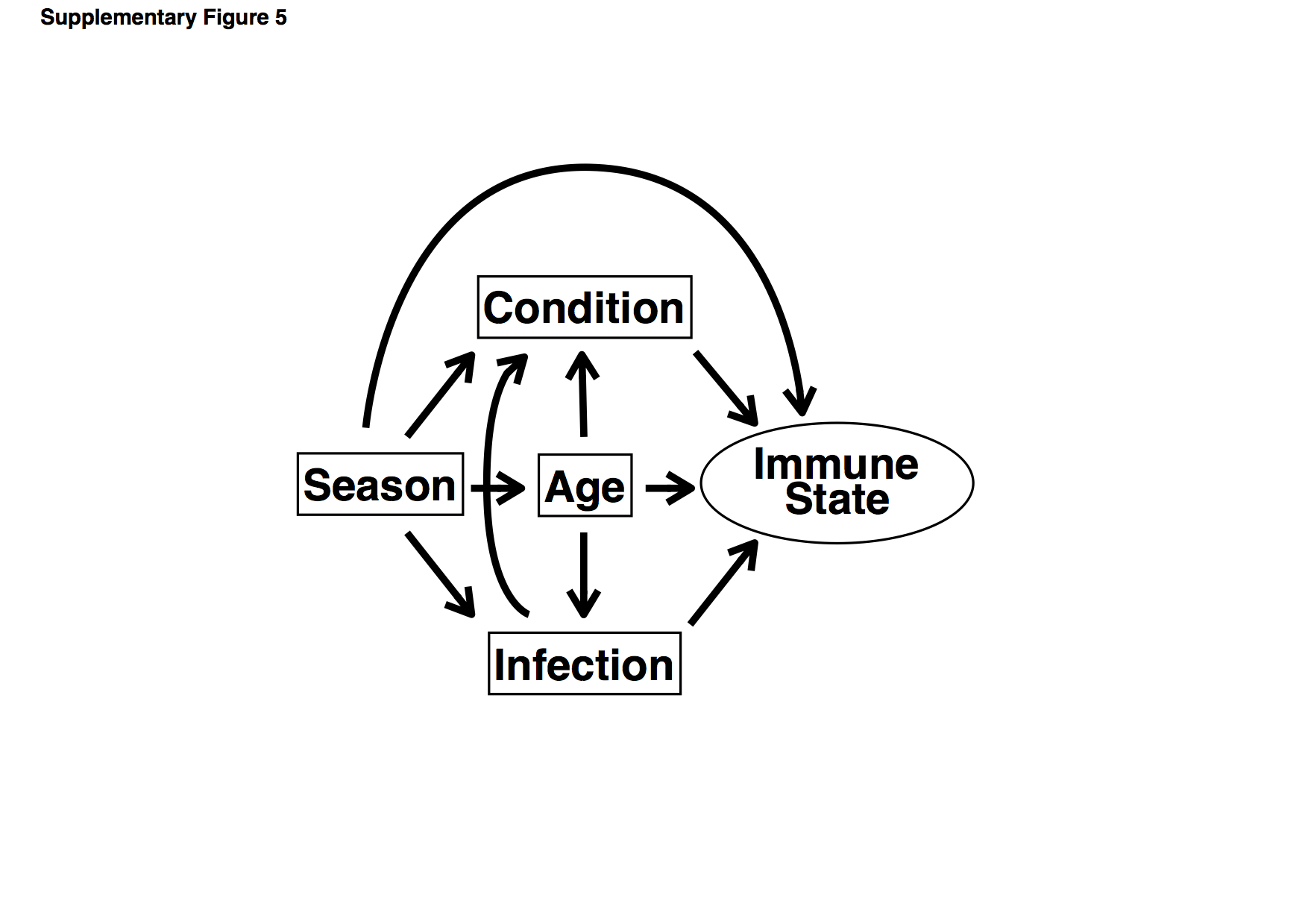

Supplement: S5 Fig — Full structural equation modelling (SEM) causal diagram, where the latent variable immune state can be adaptive cellular, innate cellular, or adaptive humoral immune state with season (measured as day length), body condition (measured as the scaled mass index), age in weeks, and infection with 7 microbial infections. (TIFF) [file pbio.2003538.s011.tiff]

**Female**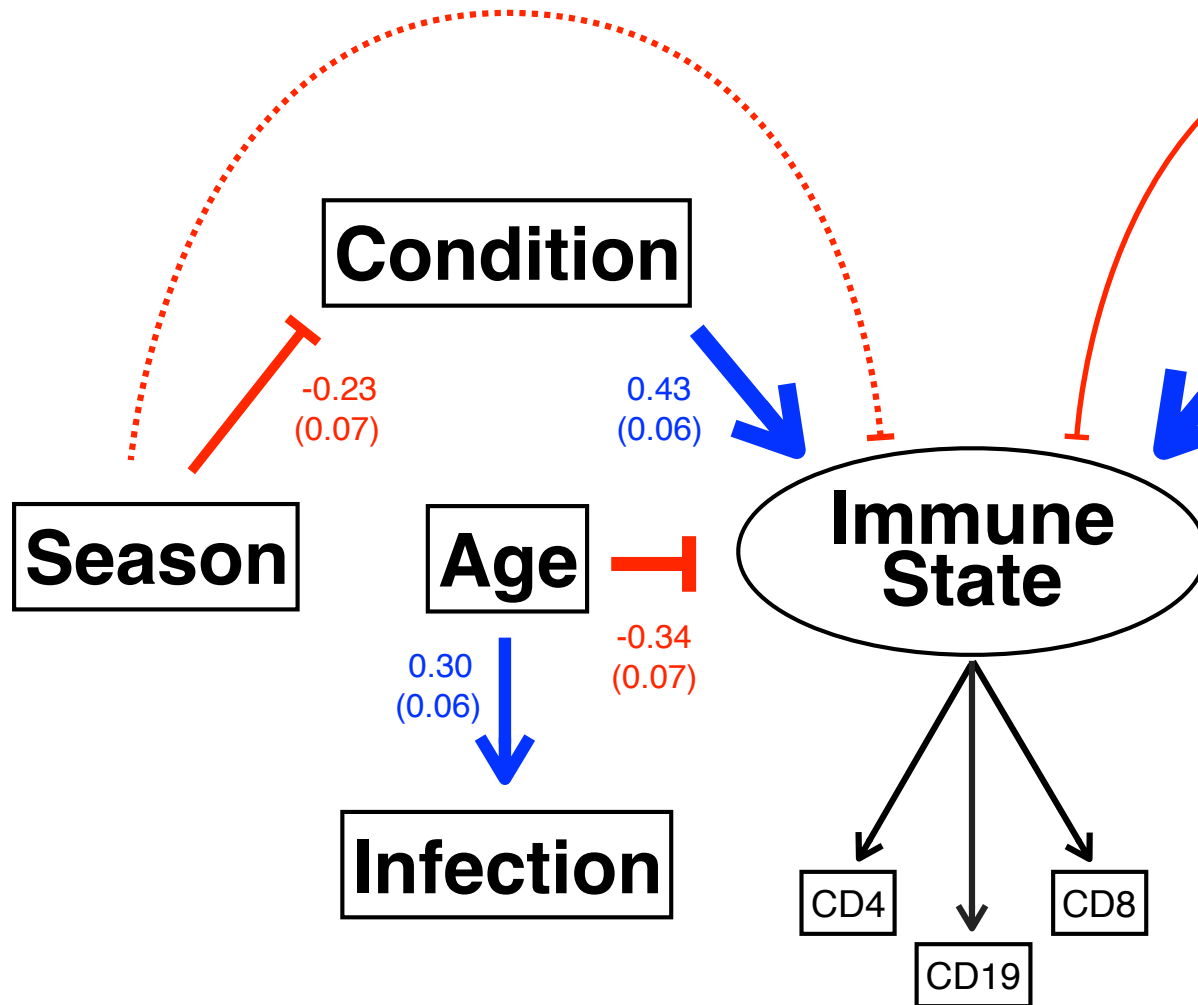**Male**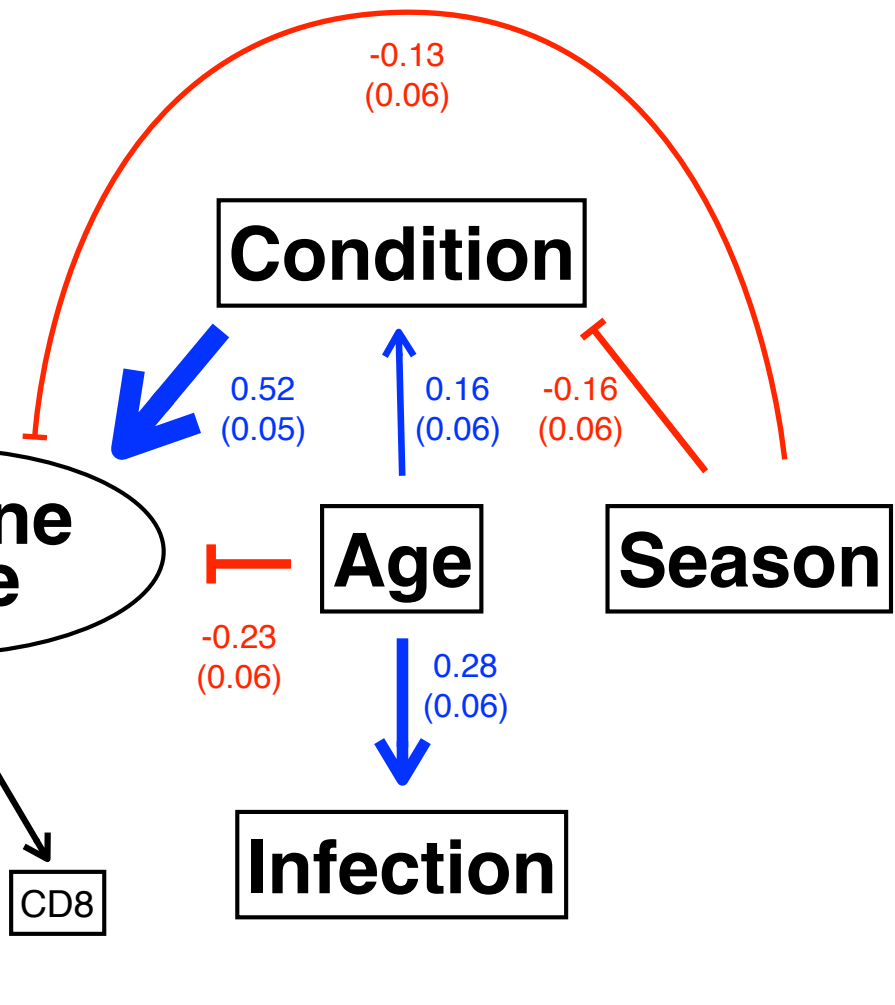

**Female****Male**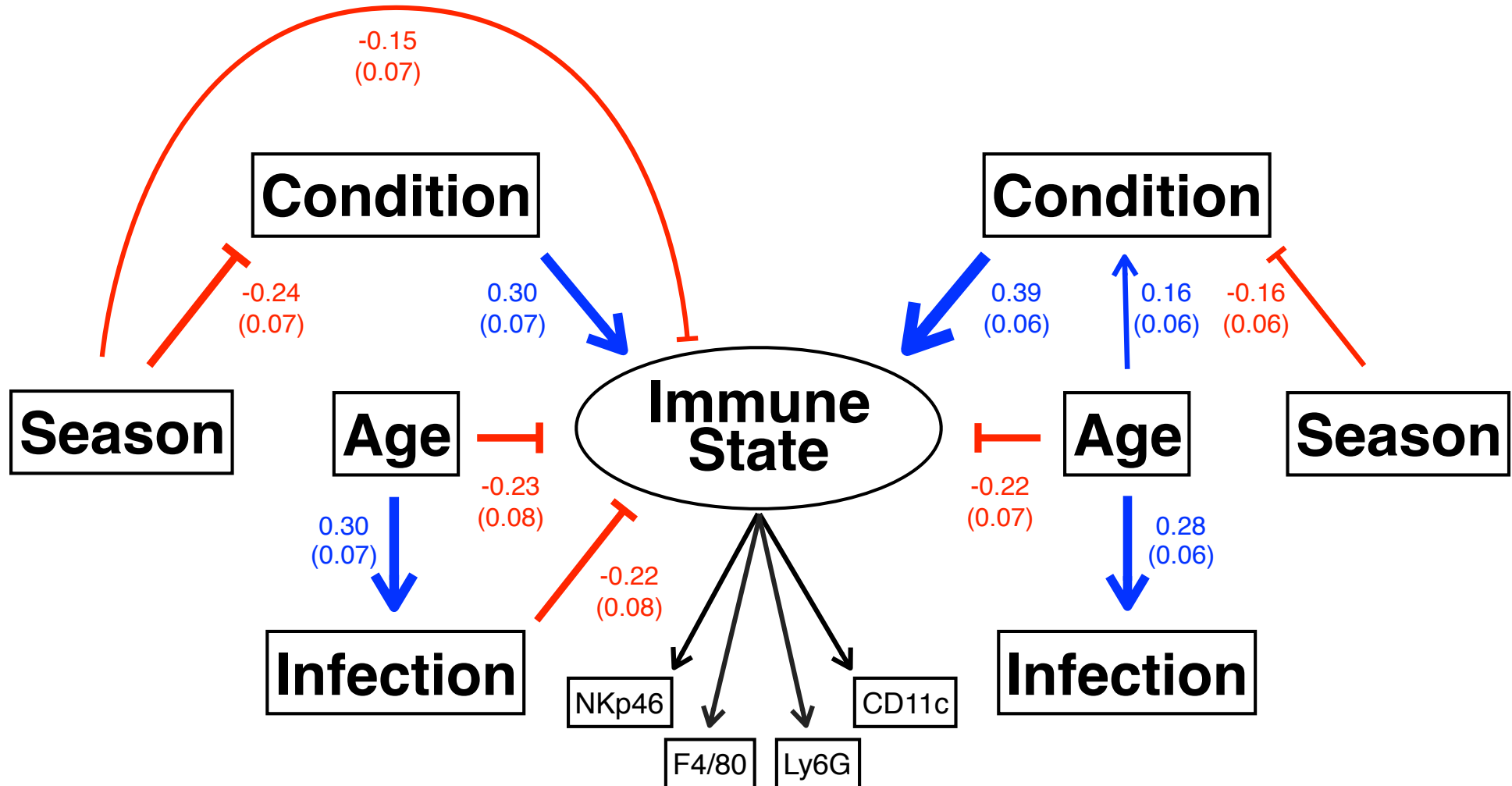

# Female

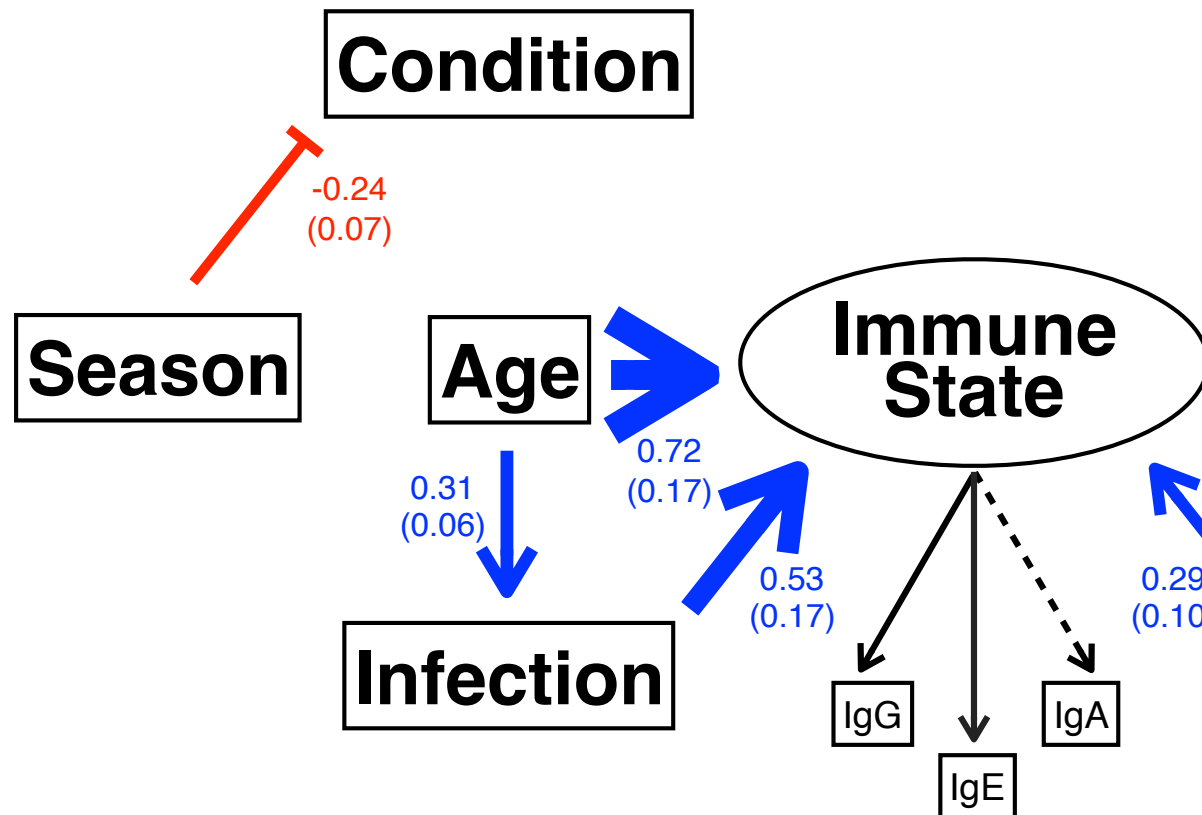

# Male

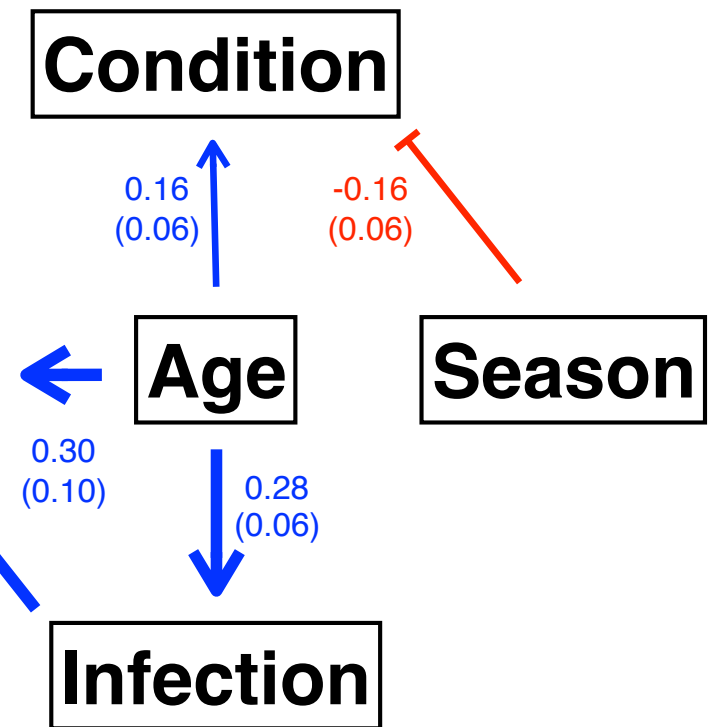

Supplement: S6 Fig — The principal drivers of immune state in wild mice. How (A) adaptive cellular, (B) innate cellular, and (C) adaptive humoral immune state is affected by Season (measured as day length), Body Condition (measured as the scaled mass index), Age in weeks, and Infection with 7 microbial infections, where blue arrows show positive effects, red blunt-ended lines show negative effects, and line thickness indicates the size of the covariance, which is shown (with the SE in parentheses) for mice from all sites; marginally nonsignificant results are shown by thin dotted lines. All estimates, SE, and p-values are shown in S6 Table. In (A), for females root mean square error of approximation (RMSEA) = 0.075 (0.026–0.124), comparative fit index (CFI) = 0.98, standardized root mean square residual (SRMR) = 0.017, χ2 = 17.61, df = 8, p = 0.024, for males RMSEA = 0.015 (0.0–0.077), CFI = 0.999, SRMR = 0.017, χ2 = 8.42, df = 8, p = 0.39; (B) for females RMSEA = 0.112 (0.08–0.146), CFI = 0.92, SRMR = 0.047, χ2 = 50.98, df = 14, p < 0.0001, which is not a significantly good fit, for males RMSEA = 0.067 (0.032–0.10), CFI = 0.972, SRMR = 0.028, χ2 = 24.43, df = 14, p = 0.009, which is not a significantly good fit; (C) for females RMSEA = 0.103 (0.06–0.149), CFI = 0.83, SRMR = 0.059, χ2 = 25.89, df = 8, p = 0.0011, with warnings concerning the latent variable immune state, for males RMSEA = 0.052 (0.0–0.099), CFI = 0.93, SRMR = 0.036, χ2 = 13.41, df = 8, p = 0.098; for immunoglobulin (Ig) A and the latent variable of immune state, the dotted line indicates that this is significant in females but nonsignificant in males, as shown in S6 Table. (PDF) [file pbio.2003538.s012.pdf]
