# Supplementary material for: The ecology of immune state in a wild mammal, Mus musculus domesticus
Source: PLoS Biol. 2018 Apr 13;16(4):e2003538. doi: 10.1371/journal.pbio.2003538 (PMC5919074; doi:10.1371/journal.pbio.2003538)
Supplement: S1 Table — (A) Mouse sample sites and (B) the intersite distances in km. (DOCX) [file pbio.2003538.s013.docx]

**Supplementary Table 1.** (A) Mouse sample sites and (B) the inter-site distances in km.

**(A)**

| **Site code** | **OS grid reference** | **Site description** | **Number of mice** |
| --- | --- | --- | --- |
| HW | ST 506 671 | Mixed arable and beef farm | 181 |
| PH | ST 532 595 | Dairy farm | 66 |
| JB | ST 595 652 | Stables and livery | 36 |
| BM | ST 523 686 | Grain merchant and mill | 35 |
| GL | SO 786 087 | Beef farm | 33 |
| SK | SM 737 050 | Island, bird observatory | 33 |
| WF | SO 882 004 | Dairy farm and cheese producer | 18 |
| LU | TQ 290 812 | London Underground | 18 |
| ST | ST 524 688 | Beef and dairy farm | 15 |
| PF | ST 554 710 | Dairy farm | 10 |
| WT | SO 818 058 | Dairy farm | 8 |
| SP | ST 665 670 | Dairy farm | 7 |

**(B)**

| **PH** | **JB** | **BM** | **GL** | **SK** | **WF** | **LU** | **ST** | **PF** | **WT** | **SP** |  |
| --- | --- | --- | --- | --- | --- | --- | --- | --- | --- | --- | --- |
| 8 | 9.1 | 2.3 | 50 | 181 | 50 | 180 | 2.4 | 6 | 50 | 7.9 | **HW** |
|  | 8.6 | 9.1 | 55 | 185 | 54 | 178 | 9.4 | 12 | 54.5 | 16 | **PH** |
|  |  | 7.9 | 47 | 190 | 45 | 171 | 8 | 7.2 | 46 | 15 | **JB** |
|  |  |  | 48 | 182 | 48 | 178 | 0.2 | 3.8 | 47 | 7.6 | **BM** |
|  |  |  |  | 205 | 13 | 154 | 48 | 44 | 4.3 | 46 | **GL** |
|  |  |  |  |  | 214 | 357 | 182 | 184 | 208 | 176 | **SK** |
|  |  |  |  |  |  | 143 | 48 | 44 | 8.2 | 48 | **WF** |
|  |  |  |  |  |  |  | 178 | 175 | 150 | 183 | **LU** |
|  |  |  |  |  |  |  |  | 3.6 | 47 | 7.5 | **ST** |
|  |  |  |  |  |  |  |  |  | 44 | 8.6 | **PF** |
|  |  |  |  |  |  |  |  |  |  | 47 | **WT** |
