# Supplementary material for: The ecology of immune state in a wild mammal, Mus musculus domesticus
Source: PLoS Biol. 2018 Apr 13;16(4):e2003538. doi: 10.1371/journal.pbio.2003538 (PMC5919074; doi:10.1371/journal.pbio.2003538)
Supplement: S2 Table — Immunological distance shown both within and among sample sites, shown as the mean ± 1 SE. There is no correlation between the within-site immunological distance and the number of mice at that site (r = −0.043, p = 0.89, n = 12). (DOCX) [file pbio.2003538.s014.docx]

**Supplementary Table 2.** Immunological distance shown both within and between sample sites, shown as the mean ± 1 SE. The is no correlation between the within-site immunological distance and the number of mice at that site (*r* = -0.043, *P* = 0.89, n = 12).

| **BM** | 3.9 ± 0.52 |  |  |  |  |  |  |  |  |  |  |  |
| --- | --- | --- | --- | --- | --- | --- | --- | --- | --- | --- | --- | --- |
| **GL** | 4.21 ± 0.36 | 4.9 ± 0.48 |  |  |  |  |  |  |  |  |  |  |
| **HW** | 4.78 ± 0.25 | 5.24 ± 0.21 | 5.68 ± 0.20 |  |  |  |  |  |  |  |  |  |
| **JB** | 4.56 ± 0.27 | 5.11 ± 0.26 | 5.75 ± 0.18 | 5.57 ± 0.27 |  |  |  |  |  |  |  |  |
| **LU** | 3.19 ± 0.39 | 3.96 ± 0.49 | 4.64 ± 0.34 | 4.25 ± 0.33 | 3.0 ± 0 .70 |  |  |  |  |  |  |  |
| **PF** | 2.99 ± 0.38 | 3.74 ± 0.48 | 4.76 ± 0.33 | 3.73 ± 0.26 | 2.55 ± 0.33 | 1.20 ± 0.14 |  |  |  |  |  |  |
| **PH** | 3.04 ± 0.15 | 3.76 ± 0.17 | 4.66 ± 0.12 | 3.76 ± 0.10 | 2.37 ± 0.12 | 1.50 ± 0.05 | 1.43 ± 0.01 |  |  |  |  |  |
| **SK** | 5.69 ± 0.34 | 6.50 ± 0.37 | 7.14 ± 0.24 | 7.14 ± 0.28 | 5.55 ± 0.46 | 5.28 ± 0.40 | 6.39 ± 0.17 | 4.33 ± 0.34 |  |  |  |  |
| **SP** | 3.42 ± 0.58 | 4.08 ± 0.57 | 4.99 ± 0.44 | 3.93 ± 0.34 | 2.75 ± 0.39 | 1.65 ± 0.21 | 1.20 ± 0.07 | 8.25 ± 0.65 | 0.43 ± 0.13 |  |  |  |
| **ST** | 6.35 ± 0.99 | 6.98 ± 0.83 | 7.62 ± 0.55 | 7.40 ± 0.68 | 6.19 ± 1.42 | 5.60 ± 1.22 | 6.21 ± 0.53 | 7.02 ± 0.52 | 7.13 ± 2.18 | 9.67 ± 2.33 |  |  |
| **WF** | 4.23 ± 0.58 | 4.98 ± 0.64 | 5.70 ± 0.44 | 5.33 ± 0.47 | 3.83 ± 0.77 | 3.48 ± 0.56 | 3.80 ± 0.27 | 5.51 ± 0.42 | 4.52 ± 1.18 | 6.70 ± 1.38 | 5.74 ± 1.53 |  |
| **WT** | 3.91 ± 0.51 | 4.62 ± 0.61 | 5.36 ± 0.43 | 4.87 ± 0.41 | 3.41 ± 0.63 | 2.83 ± 0.45 | 2.81 ± 0.14 | 6.66 ± 0.62 | 2.88 ± 0.34 | 6.91 ± 1.74 | 4.62 ±1.12 | 5.23 ± 1.64 |
|  | **BM** | **GL** | **HW** | **JB** | **LU** | **PF** | **PH** | **SK** | **SP** | **ST** | **WF** | **WT** |
