# Supplementary material for: The ecology of immune state in a wild mammal, Mus musculus domesticus
Source: PLoS Biol. 2018 Apr 13;16(4):e2003538. doi: 10.1371/journal.pbio.2003538 (PMC5919074; doi:10.1371/journal.pbio.2003538)
Supplement: S3 Table — Fst values shown among all sample sites. The local population genetic structure is not driven by geographical distance (S1 Table). Mantel test for correlation between immunological distance and distance and log(distance + 1), respectively: r = 0.192, p = 0.264, and r = 0.082, p = 0.339. (DOCX) [file pbio.2003538.s015.docx]

**Supplementary Table 3.** F_st_ values shown among all sample sites. The local population genetic structure is not driven by geographical distance (**Supplementary Table 1**). Mantel test for correlation between immunological distance and distance and log(distance+1), respectively: *r* = 0.192, *P* = 0.264 and *r* = 0.082, *P* = 0.339.

| **GL** | 0.35 |  |  |  |  |  |  |  |  |  |  |
| --- | --- | --- | --- | --- | --- | --- | --- | --- | --- | --- | --- |
| **HW** | 0.47 | 0.45 |  |  |  |  |  |  |  |  |  |
| **JB** | 0.46 | 0.43 | 0.52 |  |  |  |  |  |  |  |  |
| **LU** | 0.31 | 0.27 | 0.45 | 0.42 |  |  |  |  |  |  |  |
| **PF** | 0.42 | 0.40 | 0.49 | 0.53 | 0.35 |  |  |  |  |  |  |
| **PH** | 0.46 | 0.42 | 0.50 | 0.51 | 0.41 | 0.52 |  |  |  |  |  |
| **SK** | 0.56 | 0.57 | 0.60 | 0.66 | 0.56 | 0.68 | 0.63 |  |  |  |  |
| **SP** | 0.45 | 0.44 | 0.55 | 0.56 | 0.38 | 0.57 | 0.55 | 0.75 |  |  |  |
| **ST** | 0.23 | 0.26 | 0.38 | 0.40 | 0.22 | 0.32 | 0.41 | 0.54 | 0.37 |  |  |
| **WF** | 0.40 | 0.36 | 0.47 | 0.50 | 0.35 | 0.48 | 0.48 | 0.65 | 0.54 | 0.33 |  |
| **WT** | 0.38 | 0.36 | 0.49 | 0.51 | 0.32 | 0.48 | 0.50 | 0.68 | 0.57 | 0.30 | 0.45 |
|  | **BM** | **GL** | **HW** | **JB** | **LU** | **PF** | **PH** | **SK** | **SP** | **ST** | **WF** |
