# Supplementary material for: The ecology of immune state in a wild mammal, Mus musculus domesticus
Source: PLoS Biol. 2018 Apr 13;16(4):e2003538. doi: 10.1371/journal.pbio.2003538 (PMC5919074; doi:10.1371/journal.pbio.2003538)
Supplement: S4 Table — A summary of selected class 1–3 structural equation models showing the latent variables and observed variables used. L is a latent variable, and O is an observed variable. 1Previous Infection is the number of microbial infections and so an observed variable. Relevant structural equation modelling (SEM) diagrams are shown in S3 Fig. (DOCX) [file pbio.2003538.s016.docx]

**Supplementary Table 4.** A summary of selected class 1-3 Structural Equation Models showing the latent variables and observed variables used. *L* is a latent variable, *O* is an observed variable. ^1^Previous Infection is the number of microbial infections, and so an observed variable. Relevant SEM diagrams are shown in **Supplementary Fig. 3.**

| **Model** | **Age** | **Size** | **Season** | **Condition** | **Infcetion** | | | | | | **Immune State** | | | | | | | | | |
| --- | --- | --- | --- | --- | --- | --- | --- | --- | --- | --- | --- | --- | --- | --- | --- | --- | --- | --- | --- | --- |
|  |  |  |  |  | **Previous Infection** | **Current Infection** | **Infection** | **Mites** | **Worms** | **Microbial Infections** | **Activated Immune State** | **Adaptive Immunity** | | **Innate Immunity** | **Immune Experience** | **Immune Capacity** | **Immune State** | **IgG** | **T cells** | **B cells** |
| *L/O* | *O* | *L* | *O* | *L* | *L* | *L* | *L* | *O* | *O* | *O* | *L* | *L* | *L* | | *L* | *L* | *L* | *O* | *L* | *L* |
| **1.10** | x | x |  |  | x | x |  |  |  |  | x |  |  | |  |  |  |  |  |  |
| **1.49** | x | x |  | x | x | x |  |  |  |  |  | x | x | |  |  |  |  |  |  |
| **1.66** | x |  |  | x |  |  |  |  |  | x |  | x | x | |  |  |  | x |  |  |
| **2.2** | x |  | x | x |  |  | x |  |  |  |  |  |  | | x | x |  |  |  |  |
| **2.4** | x |  | x | x |  |  | x |  |  |  |  |  |  | | x |  |  |  |  |  |
| **2.5** | x |  | x | x |  |  | x |  |  |  |  |  |  | |  | x |  |  |  |  |
| **2.14** | x |  | x | x | x^1^ | x |  |  |  | ^1^ |  |  |  | |  |  |  |  | x | x |
| **2.19** | x |  | x | x | x^1^ | x |  |  |  | ^1^ |  |  |  | | x | x |  |  |  |  |
| **3.1** | x | x | x | x |  |  |  | x |  | x |  |  |  | |  |  | x |  |  |  |
| **3.7** | x |  | x | x |  |  | x |  |  |  |  |  |  | |  |  | x |  |  |  |
| **3.10** | x |  | x | x |  |  |  | x | x | x |  |  |  | |  |  | x |  |  |  |
| **3.11** | x | x | x | x |  |  | x |  |  |  |  |  |  | |  |  | x |  |  |  |
| **3.12** | x | x | x | x |  |  | x |  |  |  |  |  |  | |  |  | x |  |  |  |
