# Supplementary material for: The ecology of immune state in a wild mammal, Mus musculus domesticus
Source: PLoS Biol. 2018 Apr 13;16(4):e2003538. doi: 10.1371/journal.pbio.2003538 (PMC5919074; doi:10.1371/journal.pbio.2003538)
Supplement: S5 Table — A summary of the class 4 structural equation models presented in the sequence in which they were tested, with intervening rows showing the model modifications that preceded each new model. In all models, Age was an observed variable of either eye lens mass (Lens mass) or Age in weeks calculated from eye lens mass as described in the main text; Size was a latent variable consisting of a range of different measures, as shown; Condition was an observed variable of scaled mass index (SMI), the concentration of leptin, or the mass of abdominal fat / body length ratio; Immune State was either a latent variable consisting of the shown immunological parameters, as absolute numbers or percentages of the relevant cells, and concentrations of the different immunoglobulin classes, or a single observed variable as shown. In all models, Season was an observed variable of day length but is not shown in the table. Models were used with data from male and female mice separately. Relevant structural equation modelling (SEM) diagrams are shown in S4 Fig. The goodness of fit to the data for each model was assessed by the root mean square error of approximation (RMSEA), the comparative fit index (CFI), the standardized root mean square residual (SRMR), and the chi-square test of model fit (χ2), where RMSEA and SRMR values less than 0.05 were accepted as a good fit of the model to the data; however, RMSEA values greater than 0.05 were accepted where the lower 90% confidence limit was 0.000. CFI values greater than 0.95 were accepted as a very good fit, and anything greater than 0.80 was considered as acceptable. Nonsignificance (p > 0.05) for the χ2 goodness-of-fit test was used to indicate an acceptable fit of the model to the data. The software also generated warnings concerning component data of the model, and this information was used to refine the model design. (DOCX) [file pbio.2003538.s017.docx]

**Supplementary Table 5**. A summary of the class 4 Structural Equation Models presented in the sequence in which they were tested, with intervening rows showing the model modifications that preceded each new model. In all models, Age was an observed variable of either eye lens mass (Lens mass) or Age in weeks calculated from eye lens mass as described in the main text; Size was a latent variable consisting of a range of different measures, as shown; Condition was an observed variable of SMI, the concentration of leptin, or the mass of abdominal fat / body length ratio; Immune State was either a latent variable consisting of the shown immunological parameters, as absolute numbers or percentages of the relevant cells, and concentrations of the different immunoglobulin classes, or a single observed variable as shown. In all models, Season was an observed variable of day length, but is not shown in the table. Models were used with data from male and female mice separately. Relevant SEM diagrams are shown in **Supplementary Fig. 4.** The goodness of fit to the data for each model was assessed by the Root Mean Square Error of Approximation (RMSEA), the Comparative Fit Index (CFI), the Standardized Root Mean Square Residual (SRMR), and the chi-square test of model fit (χ^2^) where RMSEA and SRMR values less than 0.05 were accepted as a good fit of the model to the data, however, RMSEA values greater than 0.05 were accepted where the lower 90% confidence limit was 0.000. CFI values greater than 0.95 were accepted as a very good fit, and anything greater then 0.80 was considered as acceptable. Non-significance (*P* > 0.05) for the χ^2^ goodness of fit test was used to indicate an acceptable fit of the model to the data. The software also generated warnings concerning component data of the model, and this information was used to refine the model design.

| **Model** | **Age** | **Size** | **Condition** | **Infection** | **Immune State** | **RMSEA** | **CFI** | **SRMR** | **Χ^2^**  **(df; *P value)*** | **Warnings** |
| --- | --- | --- | --- | --- | --- | --- | --- | --- | --- | --- |
| **4.1** | Lens mass | Mass processed, body length, skull width | SMI | Microbe | CD4, CD8, CD19, NKp46, Ly6G, CD11c, F480, IgG, IgE, IgA | 0.176 | 0.730 | 0.176 | 380  (109; <0.0001) | NkP46, mass processed |
| Removed NKp46 | | | | | | | | | | |
| **4.2** | Lens mass | Mass processed, body length, skull width | SMI | Microbe | CD4, CD8, CD19, Ly6G, CD11c, F480, IgG, IgE, IgA | 0.188 | 0.716 | 0.201 | 358  (94; <0.0001) | CD8, mass processed |
| Removed CD8 | | | | | | | | | | |
| **4.3** | Lens mass | Mass processed, body length, skull width | SMI | Microbe | CD4, CD19, Ly6G, CD11c, F480, IgG, IgE, IgA | 0.141 | 0.834 | 0.164 | 207  (80; <0.0001) | Ly6G, mass processed |
| Removed Ly6G | | | | | | | | | | |
| **4.4** | Lens mass | Mass processed, body length, skull width | SMI | Microbe | CD4, CD19, CD11c, F480, IgG, IgE, IgA | 0.137 | 0.859 | 0.171 | 167  (67; <0.0001) | F480, mass processed |
| Removed F480 | | | | | | | | | | |
| **4.5** | Lens mass | Mass processed, body length, skull width | SMI | Microbe | CD4, CD19, CD11c, IgG, IgE, IgA | 0.138 | 0.869 | 0.145 | 139  (55; <0.0001) | Mass processed |
| Replaced mass processed with mass trapped | | | | | | | | | | |
| **4.6** | Lens mass | Mass trapped, body length, skull width | SMI | Microbe | CD4, CD19, CD11c, IgG, IgE, IgA | 0.107 | 0.891 | 0.087 | 105  (78; <0.0001) | Mass trapped |
| Replaced mass trapped with kidney mass | | | | | | | | | | |
| **4.7** | Lens mass | Kidney mass, body length, skull width | SMI | Microbe | CD4, CD19, CD11c, IgG, IgE, IgA | 0.080 | 0.916 | 0.116 | 83.5  (55; 0.0079) | SMI |
| Replaced body length with tail length | | | | | | | | | | |
| **4.8** | Lens mass | Kidney mass, tail length, skull width | SMI | Microbe | CD4, CD19, CD11c, IgG, IgE, IgA | - | - | - | - | No convergence |
| Replaced tail length with heart and lung mass | | | | | | | | | | |
| **4.9** | Lens mass | Kidney mass, heart and lung mass, skull width | SMI | Microbe | CD4, CD19, CD11c, IgG, IgE, IgA | 0.078 | 0.906 | 0.110 | 81.7  (55; 0.011) | No warnings |
| Removed antibodies | | | | | | | | | | |
| **4.10** | Lens mass | Kidney mass, heart and lung mass, skull width | SMI | Microbe | CD4, CD19, CD11c | 0.040 | 0.986 | 0.088 | 28.19  (25; 0.30) | Immune state |
| Added CD8 back into model | | | | | | | | | | |
| **4.11** | Lens mass | Kidney mass, heart and lung mass, skull width | SMI | Microbe | CD4, CD19, CD11c, CD8 | 0.060 | 0.965 | 0.083 | 43.68  (34; 0.12) | Immune state |
| Added NKp46 back into model | | | | | | | | | | |
| **4.12** | Lens mass | Kidney mass, heart and lung mass, skull width | SMI | Microbe | CD4, CD19, CD11c, CD8, NKp46 | 0.087 | 0.924 | 0.104 | 70.5  (44; 0.0068) | Immune state |
| Added Ly6G back into model | | | | | | | | | | |
| **4.13** | Lens mass | Kidney mass, heart and lung mass, skull width | SMI | Microbe | CD4, CD19, CD11c, CD8, NKp46, Ly6G | 0.077 | 0.933 | 0.106 | 81.2  (55; 0.012) | Immune state |
| Removed all cells except CD4 | | | | | | | | | | |
| **4.14** | Lens mass | Kidney mass, heart and lung mass, skull width | SMI | Microbe | CD4 | 0.000 | 1.000 | 0.063 | 10.7  (11; 0.47) | CD4 |
| Replaced CD4 with CD8 | | | | | | | | | | |
| **4.15** | Lens mass | Kidney mass, heart and lung mass, skull width | SMI | Microbe | CD8 | 0.000 | 1.000 | 0.065 | 9.9  (11; 0.54) | CD8 |
| Replaced CD8 with CD19 | | | | | | | | | | |
| **4.16** | Lens mass | Kidney mass, heart and lung mass, skull width | SMI | Microbe | CD19 | 0.037 | 0.993 | 0.081 | 12.21  (11; 0.35) | No warnings |
| Replaced CD19 with CD11c | | | | | | | | | | |
| **4.17** | Lens mass | Kidney mass, heart and lung mass, skull width | SMI | Microbe | CD11c | 0.048 | 0.988 | 0.065 | 13.06  (11; 0.29) | No warnings |
| Replaced CD11c with NKp46 | | | | | | | | | | |
| **4.18** | Lens mass | Kidney mass, heart and lung mass, skull width | SMI | Microbe | NKp46 | 0.041 | 0.992 | 0.061 | 12.47  (11; 0.33) | No warnings |
| Replaced NKp46 with F480 | | | | | | | | | | |
| **4.19** | Lens mass | Kidney mass, heart and lung mass, skull width | SMI | Microbe | F480 | 0.049 | 0.988 | 0.067 | 13.11  (11; 0.29) | No warnings |
| Replaced NKp46 with Ly6G | | | | | | | | | | |
| **4.20** | Lens mass | Kidney mass, heart and lung mass, skull width | SMI | Microbe | Ly6G | 0.022 | 0.998 | 0.064 | 11.42  (11; 0.41) | No warnings |
| Used all 3 antibodies for Immune State | | | | | | | | | | |
| **4.21** | Lens mass | Kidney mass, heart and lung mass, skull width | SMI | Microbe | IgG, IgE, IgA | 0.032 | 0.990 | 0.072 | 27.1  (25; 0.35) | Immune state |
| Removed IgE and IgA | | | | | | | | | | |
| **4.22** | Lens mass | Kidney mass, heart and lung mass, skull width | SMI | Microbe | IgG | 0.000 | 1.000 | 0.054 | 8.07  (11; 0.71) | No warnings |
| Replaced IgG with IgA | | | | | | | | | | |
| **4.23** | Lens mass | Kidney mass, heart and lung mass, skull width | SMI | Microbe | IgA | 0.000 | 1.000 | 0.054 | 10.22  (11; 0.51) | No warnings |
| Replaced IgA with IgE | | | | | | | | | | |
| **4.24** | Lens mass | Kidney mass, heart and lung mass, skull width | SMI | Microbe | IgE | 0.000 | 1.000 | 0.056 | 10.40  (11; 0.50) | No warnings |
| Removed size variables, and repeated model 4.9, which had the greatest number of immune variables in a model that ran without warnings. | | | | | | | | | | |
| **4.25** | Lens mass | - | SMI | Microbe | CD4, CD19, CD11c, IgG, IgE, IgA | 0.095 | 0.906 | 0.095 | 49.95  (29; 0.0091) | No warnings |
| Replaced SMI with leptin | | | | | | | | | | |
| **4.26** | Lens mass | - | Leptin | Microbe | CD4, CD19, CD11c, IgG, IgE, IgA | 0.137 | 0.733 | 0.107 | 72.3  (29; <0.0001) | No warnings |
| Added Size latent variable back in, thus a repeat of model 4.9 but with Leptin instead of SMI | | | | | | | | | | |
| **4.27** | Lens mass | Kidney mass, heart and lung mass, skull width | Leptin | Microbe | CD4, CD19, CD11c, IgG, IgE, IgA | 0.172 | 0.575 | 0.138 | 185  (55; <0.0001) | No warnings |
| Replaced lens mass with calculated age | | | | | | | | | | |
| **4.28** | Age | Kidney mass, heart and lung mass, skull width | Leptin | Microbe | CD4, CD19, CD11c, IgG, IgE, IgA | 0.079 | 0.897 | 0.112 | 82.5  (55; 0.0096) | No warnings |
| Replaced counts of immune cells with proportions, and age with Lens mass | | | | | | | | | | |
| **4.29** | Lens mass | Kidney mass, heart and lung mass, skull width | Leptin | Microbe | %CD4, %CD19, %CD11c, IgG, IgE, IgA | 0.036 | 0.976 | 0.100 | 59.5  (54; 0.28) | Immune state |
| Repeated model 4.9 but removed the model interaction between Season and Lens mass | | | | | | | | | | |
| **4.30** | Lens mass | Kidney mass, heart and lung mass, skull width | SMI | Microbe | CD4, CD19, CD11c, IgG, IgE, IgA | 0.086 | 0.899 | 0.123 | 85.22  (54; 0.0043) | No warnings |
| Replaced SMI with abdominal fat mass divided by body length, and reintroduced the model interaction between Season and Lens mass | | | | | | | | | | |
| **4.31** | Lens mass | Kidney mass, heart and lung mass, skull width | Ab. fat / length | Microbe | CD4, CD19, CD11c, IgG, IgE, IgA | 0.085 | 0.875 | 0.107 | 85.1  (54; 0.0044) | No warnings |
