# Supplementary material for: The ecology of immune state in a wild mammal, Mus musculus domesticus
Source: PLoS Biol. 2018 Apr 13;16(4):e2003538. doi: 10.1371/journal.pbio.2003538 (PMC5919074; doi:10.1371/journal.pbio.2003538)
Supplement: S7 Table — The estimated standardised covariances (Estimate), their standard error (SE), and 2-tailed p-values (with p < 0.05 shown in bold) for structural equation models of adaptive cellular, innate cellular, and adaptive humoral immune state for female and male mice from site HW, as shown in Fig 5. Marginally nonsignificant results are marked with *. (DOCX) [file pbio.2003538.s019.docx]

**Supplementary Table 7.** The estimated standardised covariances (Estimate), their standard error (S.E.), and two-tailed *P* values (with *P <* 0.05 shown in bold) for Structural Equation Models of Adaptive Cellular, Innate Cellular, and Adaptive Humoral immune state for female and male mice from site HW, as shown in **Fig. 5**. Marginally non-significant results are marked with *.

|  | **Estimate** | **S.E.** | ***P*** |
| --- | --- | --- | --- |
|  |  |  |  |
| **Female, Adaptive Cellular** | | | |
| *Immune State:* |  |  |  |
| **CD4** | **0.978** | **0.019** | **<0.0001** |
| **CD8** | **0.952** | **0.022** | **<0.0001** |
| **CD19** | **0.696** | **0.080** | **<0.0001** |
| *Immune State:* |  |  |  |
| **Season** | **-0.338** | **0.116** | **0.004** |
| **Age** | **-0.520** | **0.093** | **<0.0001** |
| **Condition** | **0.726** | **0.101** | **<0.0001** |
| **Infection** | **-0.233** | **0.110** | **0.034** |
| *Infection:* |  |  |  |
| **Age** | **0.209** | **0.103** | **0.042** |
| **Season** | **-0.352** | **0.104** | **0.001** |
| *Condition:* |  |  |  |
| **Age** | **0.440** | **0.096** | **<0.0001** |
| Season | -0.001 | 0.111 | 0.993 |
| Infection | -0.097 | 0.117 | 0.408 |
| *Age:* |  |  |  |
| Season | -0.195 | 0.107 | 0.068* |
|  |  |  |  |
| **Male, Adaptive Cellular** | | | |
| *Immune State:* |  |  |  |
| **CD4** | **0.999** | **0.017** | **<0.0001** |
| **CD8** | **0.899** | **0.028** | **<0.0001** |
| **CD19** | **0.856** | **0.037** | **<0.0001** |
| *Immune State:* |  |  |  |
| **Season** | **-0.189** | **0.092** | **0.041** |
| **Age** | **-0.301** | **0.095** | **0.002** |
| **Condition** | **0.617** | **0.076** | **<0.0001** |
| Infection | -0.046 | 0.113 | 0.683 |
| *Infection:* |  |  |  |
| Age | 0.157 | 0.108 | 0.145 |
| Season | -0.177 | 0.110 | 0.106 |
| *Condition:* |  |  |  |
| Age | 0.193 | 0.099 | 0.051* |
| Season | -0.164 | 0.100 | 0.099 |
| Infection | 0.000 | 0.110 | 1.000 |
| *Age:* |  |  |  |
| **Season** | **-0.245** | **0.094** | **0.009** |
|  |  |  |  |
| **Female, Innate Cellular** | | | |
| *Immune State:* |  |  |  |
| **NKp46** | **0.877** | **0.038** | **<0.0001** |
| **F4/80** | **0.648** | **0.090** | **<0.0001** |
| **Ly6G** | **0.979** | **0.022** | **<0.0001** |
| **CD11c** | **0.865** | **0.039** | **<0.0001** |
| *Immune State:* |  |  |  |
| **Season** | **-0.337** | **0.119** | **0.005** |
| **Age** | **-0.412** | **0.106** | **<0.0001** |
| **Condition** | **0.522** | **0.114** | **<0.0001** |
| **Infection** | **-0.288** | **0.132** | **0.029** |
| *Infection:* |  |  |  |
| **Age** | **0.212** | **0.103** | **0.040** |
| **Season** | **-0.349** | **0.104** | **0.001** |
| *Condition:* |  |  |  |
| **Age** | **0.439** | **0.096** | **<0.0001** |
| Season | -0.001 | 0.110 | 0.994 |
| Infection | -0.098 | 0.117 | 0.404 |
| *Age:* |  |  |  |
| Season | -0.196 | 0.107 | 0.067* |
|  |  |  |  |
| **Male, Innate Cellular** | | | |
| *Immune State:* |  |  |  |
| **NKp46** | **0.940** | **0.025** | **<0.0001** |
| **F4/80** | **0.799** | **0.055** | **<0.0001** |
| **Ly6G** | **0.867** | **0.044** | **<0.0001** |
| **CD11c** | **0.878** | **0.038** | **<0.0001** |
| *Immune State:* |  |  |  |
| Season | -0.185 | 0.100 | 0.064* |
| **Age** | **-0.326** | **0.099** | **0.001** |
| **Condition** | **0.513** | **0.091** | **<0.0001** |
| Infection | -0.181 | 0.127 | 0.154 |
| *Infection:* |  |  |  |
| Age | 0.168 | 0.107 | 0.116 |
| Season | -0.174 | 0.109 | 0.112 |
| *Condition:* |  |  |  |
| Age | 0.194 | 0.099 | 0.050* |
| Season | -0.165 | 0.099 | 0.098 |
| Infection | -0.001 | 0.109 | 0.994 |
| *Age:* |  |  |  |
| **Season** | **-0.243** | **0.095** | **0.010** |
|  |  |  |  |
| **Female, Adaptive Humoral** | | | |
| *Immune State:* |  |  |  |
| **IgG** | **0.503** | **0.117** | **<0.0001** |
| **IgE** | **0.356** | **0.117** | **0.002** |
| **IgA** | **0.594** | **0.114** | **<0.0001** |
| *Immune State:* |  |  |  |
| Season | -0.142 | 0.152 | 0.350 |
| **Age** | **0.662** | **0.165** | **<0.0001** |
| Condition | 0.228 | 0.149 | 0.127 |
| **Infection** | **0.414** | **0.165** | **0.012** |
| *Infection:* |  |  |  |
| **Age** | **0.209** | **0.102** | **0.041** |
| **Season** | **-0.363** | **0.102** | **<0.0001** |
| *Condition:* |  |  |  |
| **Age** | **0.437** | **0.096** | **<0.0001** |
| Season | 0.001 | 0.111 | 0.993 |
| Infection | -0.089 | 0.118 | 0.449 |
| *Age:* |  |  |  |
| Season | -0.196 | 0.107 | 0.067* |
|  |  |  |  |
| **Male, Adaptive Humoral** | | | |
| *Immune State:* |  |  |  |
| **IgG** | **0.353** | **0.160** | **0.027** |
| **IgE** | **0.520** | **0.199** | **0.009** |
| IgA | 0.210 | 0.195 | 0.282 |
| *Immune State:* |  |  |  |
| Season | 0.166 | 0.167 | 0.321 |
| **Age** | **0.633** | **0.241** | **0.009** |
| Condition | -0.0221 | 0.168 | 0.188 |
| Infection | 0.393 | 0.205 | 0.055* |
| *Infection:* |  |  |  |
| Age | 0.171 | 0.107 | 0.109 |
| Season | -0.168 | 0.109 | 0.125 |
| *Condition:* |  |  |  |
| **Age** | **0.195** | **0.099** | **0.048** |
| Season | -0.166 | 0.099 | 0.095 |
| Infection | -0.066 | 0.109 | 0.957 |
| *Age on:* |  |  |  |
| **Season** | **-0.242** | **0.095** | **0.011** |
