# Supplementary material for: The ecology of immune state in a wild mammal, Mus musculus domesticus
Source: PLoS Biol. 2018 Apr 13;16(4):e2003538. doi: 10.1371/journal.pbio.2003538 (PMC5919074; doi:10.1371/journal.pbio.2003538)
Supplement: S1 Text — Extended methods for structural equation modelling. (DOCX) [file pbio.2003538.s020.docx]

**Supplementary Text 1.** Extended methods for Structural Equation Modelling.

To analyse and understand our data we constructed and tested a large number of different Structural Equation Models (SEM). We developed 5 basic classes of SEMs and for each there were multiple individual variants. In all we constructed and tested 230 SEMs. Here we describe the rationale, components and structure of these SEMs, and the results that were obtained from them. We used the results obtained from one model to develop and refine new models. This process provides the basis for the development of the final model that we present in the main text.

Overall, our favoured initial approach was to use latent variables where reasonable because this would use many of the multiple measures available in our data set. We used basic biological reasoning in determining the structure of these models, but we discuss this further below. During our iterative development of models, the general trend was to (i) use fewer latent variables, (ii) use separate models for male and female mice, and (iii) move from analysing the data from all 12 samples sites together, to analysing a single sample site. A selection of class 1-3 models and their respective latent and observed variables are shown in **Supplementary Table 4** and **Supplementary Fig. 3.** All class 4 models and their latent and observed variables are shown in **Supplementary Table 5** and **Supplementary Fig. 4**.

**Class 1-3 models.**

We used biological reasoning in constructing and developing different models. During this process we were particularly mindful of the potential sequential temporal effects of different variables during an animal’s life, particularly concerning Immune State and Infection. By way of example, in model 2.19, Previous Infection had unidirectional effects on Current Infection and on Condition, but there were bidirectional effects between Condition and Current Infection. In model 3.11, there were unidirectional effects of Age on Size, and on Infection, but bidirectional effects among Condition, Immune State, and Infection. During the model development we also explored a range of different observed and latent variables which are described below. Very few class 1-3 models converged, or when they did they were not significant. During analysis of class 3 models we explored analysing data for male and female mice separately, finding that this facilitated progress, and so we persisted with this approach.

***Age*.** In all models this was an observed variable of age in weeks, estimated from eye lens mass, as described in the main text.

***Size*.** Models included Size as a latent variable consisting of a range of different measures, including, (i) body length, tail length, mass when killed; (ii) body length, tail length, skull length; (iii) body length, skull length, skull width, mass when caught; (iv) body length, skull length, skull width, mass when caught, mass of liver, kidney heart and lungs; (v) body length, mass when caught. Using these Size latent variables we sought to account for all size effects within these populations, but especially the effects of size on measures of immune state and on body condition.

***Season***. In class 2 and 3 models, Season was an observed variable of the day length when the mouse was caught.

***Condition***. Models included Condition as a latent variable consisting of (i) mass of abdominal fat, concentration of haemoglobin; (ii) mass of abdominal fat, concentration of haemoglobin, concentration of leptin; (iii) mass of abdominal fat, concentration of haemoglobin, concentration of leptin, body mass / body length ratio; (iv) mass of abdominal fat / body length ratio, concentration of haemoglobin, concentration of leptin, body mass / body length ratio; (v) concentration of leptin, concentration of haemoglobin, mass of abdominal fat / body length ratio, body mass / body length ratio; (vi) mass of abdominal fat, concentration of haemoglobin, concentration of leptin, body mass / body length ratio; (vii) concentration of haemoglobin, concentration of leptin, abdominal fat mass / body length ratio; (viii) concentration of leptin, concentration of haemoglobin, scaled mass index (SMI). The rationale for using these factors in these latent variables was to include factors that have previously been used as measures of condition, together with other data we had available that may additionally represent an aspect of condition.

***Infection***. Models used both latent and observed variables. The latent variables consisted of (i) Previous Infection, consisting of IgG concentration and the number of microbial infections; (ii) Current Infection, consisting of (a) number of splenocytes, number of worms, number of mites, concentration of IgA, concentration of IgE and %Ly6G+ SSCHi (described further under Immune State, below), or (b) number of worms, number of mites; (iii) Infection, consisting of number of worms, number of microbial infections, number of mites; observed variables consisted of the number of (iv) mites, (v) microbial infections, (vi) worms. The rationale for (i) and (ii) was that previous infections and current infections would have different effects on measures of immune state. Latterly we refined latent variables (i) and (ii) by removing the immunological measures, because this was a confounding path of logic with respect to the latent variable of Immune State (below). In (iii) we further simplified our approach to infection, using a latent variable that contained all the direct, relevant infection data only, and also treated these as separate observed variables ((iv) – (vi), above).

***Immune State*.** Models used a range of different latent variables (i) Activated Immune State consisting of number of cells with markers for CD11a+CD8+, CD11a+49d+CD8+, CD62L-CD44+CD8+, CD11a+CD4+, CD11a+CD49d-CD4+, CD62L-CD44+CD4+, CD62L+CD44-CD8+, CD62L+CD44-CD4+, MFI of MHC-II+CD19+, F4/80+CD3-, CD11c+CD3-, Ly49D-G2-H-NKp46+, Ly49D-G2+H-NKp46+; (ii) Innate Immunity consisting of number of cells with markers for F4/80+CD3-, CD11c+CD3-, Ly6G+SSCHi, the concentration of serum amyloid P component (SAP), haptoglobin, alpha-1 antitrypsin (AAT) [1]; (iii) Adaptive Immunity, consisting of number of cells with markers for CD11a+CD8+, CD11a+49d+ of CD8+, CD62L-CD44+CD8+, CD11a+CD4+, CD11a+CD49d-CD4+, CD62L-CD44+CD4+, MFI of MHC-II+CD19+; (iv) Immune Experience consisting of number of cells with markers for (a) CD11a+CD8+, CD11a+CD49+CD8+, CD11a-KLRG1-CD8+, CD11a+KLRG1-CD4+, CD11a+CD4+, CD11a+CD49d-CD4+, KLRG1+CD4+, CD11a-KLRG1+CD4+, CD11a-KLRG1+CD8+, the concentration of IgG, IgE, IgA, or (b) number of cells with markers for CD11a+CD8+, CD11a+CD49+CD8+, CD11a-KLRG1-CD8+, CD11a+KLRG1-CD4+, CD11a+CD4+, CD11a+CD49d-CD4+, KLRG1+CD4+, CD11a-KLRG1+CD4+, CD11a-KLRG1+CD8+, CD62L-CD44-CD4+, CD62L-CD44-CD8+, CD62L+CD44-CD4+, GL7+CD19+, GL7+MHC-II+CD19+, MFI of PNA+CD19+, PNA+CD19+ and IgD-CD38+CD19+, the concentration of IgG, IgE, IgA; (v) Immune Capacity, consisting of (a) number of cells with markers for Ly49H+NKp46+, Ly49D+NKp46+, Ly49D+G2-H+NKp46+, Ly49D-G2-H+NKp46+, Ly49D-G2-H-NKp46+, Ly49D-G2+H-NKp46+, CD11a-CD49d+CD8+, CD11a-CD49d+CD4+, CD49d+CD4+, CD62L-CD44-CD4+, CD62L-CD44-CD8+, CD62L+CD44-CD4+ or (b) number of cells with markers for Ly49H+NKp46+, Ly49D+NKp46+, Ly49D+G2-H+NKp46+, Ly49D-G2-H+NKp46+, Ly49D-G2-H-NKp46+, Ly49D-G2+H-NKp46+, CD11a-CD49d+CD8+, CD11a-CD49d+CD4+, CD49d+CD4+, Ly6G+CD3-, CD11c+CD3-, F4/80+CD3-; (vi) T cells consisting of number of cells with markers for CD11a+ of CD8+, CD11a+ CD49+ of CD8+, CD11a- KLRG1- of CD8+, CD11a+ KLRG1- of CD4+, CD11a+ of CD4+, CD11a+ CD49d- of CD4+, KLRG1+CD4+, CD11a- KLRG1+ CD4+, CD11a- KLRG1+ of CD8+, CD62L- CD44- of CD4, CD62L- CD44- of CD8, CD62L+ CD44- CD4+; (vii) B cells, consisting of number of cells with markers for GL7+ of CD19+, GL7+MHC-II+CD19+, MFI of PNA+CD19+, PNA+CD19+ and IgD-CD38+CD19+; (viii) Immune State consisting (a) of number of cells with markers for CD4+, CD8+, CD19+, F4/80+, Ly6G+, CD11c+, NKp46+, and the concentration of IgG, IgE, IgA, or (b) the number of CD4+ cells, and the concentration of IgE, IgE, IgA, or (c) the number of CD4+, CD19+, CD11c+ cells, and the concentration of IgG, IgE, IgA, or (d) the concentration of IgG, IgE, IgA.

The rationale of (i) was to attempt to capture how current and previous infection, along with size and age, affect the current status of individuals’ immune state by using only immune measures that captured an activated immune state. In using (ii) and (iii) the rationale was that adaptive and innate immunity might be differently affected by animals' state. Analogously in (vi) and (vii) we considered whether the T and B cell compartments of the immune system might be differently affected by animals’ state. In using (iv) and (v) we sought to separate measures of immune state that reflected past immunological state from measures that might represent future state, recognising that these may be differently affected by animals’ state. In (viii) we combined all of the principal immune measures. Further derivatives of approach (viii) were used in class 4 and 5 models.

In class 1 models, concerning latent variables (i), (ii) and (iii) we used flow cytometry defined cell populations [1], with our understanding of likely function of these cell populations in mice, to generate these latent variables, as above. In class 2 models, concerning latent variables (iv), (v), (vi) and (vii) we used a Principal Component Analysis (PCA) of the flow cytometry data [1] to identify cell populations to be included in these latent variables, in all cases using one population to represent the relevant principal component, as described above. As discussed above (see Size section), class 1, 2 and 3 models used a latent variable of Size and so the number of each class of immune cells was not scaled.

**Class 4 and 5 models.**

Thirty one class 4 models used the observed and latent variables shown in **Supplementary Table 5** with the structures shown in **Supplementary Fig. 4**. Of these, 15 ran without warnings, 3 models (4.22-4.24) were close to significant by 4 of the 4 model fit criteria, 9 by 3 of the 4 criteria and 3 by 2 of the 4 criteria. The structure of these models largely derive from models (2.4, 3.7, 3.11, 3.12; **Supplementary Fig. 3**), above. The class 4 model structures consist of two broad forms that principally differed by the presence or absence of the Size latent variable; specifically models 4.25 and 4.26 did not use this latent variable. We used many of the models which included Size to explore the inclusion of different immune parameters for the latent variable Immune State. During analyses of class 4 models we also explored using data for mice from all our sample sites, or from different sample sites separately (where sufficient data were available). This showed that analysis of site HW, or of all sites together were tractable approaches, which we therefore continued. Overall, in view of the results we obtained we favoured using model structure 4.25 **(Supplementary Fig. 5)** as the structure for our class 5 models due to it being the most inclusive, and parsimonious, model design that largely ran without warnings..

Class 5 models did not contain a Size latent or observed variable, and therefore we accounted for size in two ways in these models. Firstly, we accounted for size in our measures of cellular immune state by scaling the number of cells using the method used in calculating SMI. Secondly, by using the observed variable of SMI for Condition which implicitly accounts for size, and has been especially developed for use as a condition measure. The class 5 models explored seven different Immune State latent variables; specifically, (i) all immune state, specifically the scaled number of CD4+, CD8+, CD19+, CD11c+, NKp46+, Ly6G+, F4/80+ cells, and the concentration of IgG, IgE, IgA; (ii) humoral immune state, consisting of the scaled number of CD19+ cells, and the concentration of IgG, IgE, IgA; (iii) antibodies, consisting of the concentration of IgG, IgE, IgA; (iv) cellular immune state, consisting of the scaled number of CD4+, CD8+, CD19+, CD11c+, NKp46+, Ly6G+, F4/80+ cells; (v) adaptive cellular state consisting of the scaled number of CD4+, CD8+, CD19+ cells; (vi) innate cellular state consisting of the scaled number of CD11c+, NKp46+, Ly6G+, F4/80+ cells; (vii) adaptive cellular and humoral immune state, consisting of the scaled number of CD4+, CD8+, CD19^+^ cells, and the concentration of IgG, IgE, IgA. We also explored the effect of six different Condition observed variables, specifically, SMI, Body Mass Index (BMI), the concentration of leptin, the concentration of haemoglobin, the mass of abdominal fat, and a Scaled Fat Index (where the mass of abdominal fat was scaled analogously as for SMI). Based up on the results of this analysis our favoured approach, and what we present in the main text uses 3 models where Immune State was separately the latent variables (iii), (v), and (vi), and where SMI was the observed variable of Condition. We found that this approach gave the best cohesion of sound biological reasoning and the most robust models.
